# Identification and expression of candidate chemosensory receptors in the white-spotted flower chafer, *Protaetia brevitarsis*

**DOI:** 10.1038/s41598-019-38896-x

**Published:** 2019-03-04

**Authors:** Hongmin Liu, Xiaofang Zhang, Chunqin Liu, Yongqiang Liu, Xiangdong Mei, Tao Zhang

**Affiliations:** 1 Agriculture College, Xinyang Agriculture and Forestry University, Xinyang, 464000 P. R. China; 2 Institute of Plant Protection, Hebei Academy of Agriculture and Forestry Sciences/Integrated Pest Management Center of Hebei Province/Key Laboratory of IPM on Crops in Northern Region of North China, Ministry of Agriculture, Baoding, 071000 P. R. China; 3 Cangzhou Technical College, Cangzhou, 061001 P. R. China; 4 State Key Laboratory for Biology of Plant Diseases and Insect Pests, Institute of Plant Protection, Chinese Academy of Agricultural Sciences, Beijing, 100193 P. R. China

## Abstract

Accurate detection and recognition of chemical signals play extremely important roles for insects in their survival and reproduction. Chemosensory receptors, including odorant receptors (ORs), ionotropic receptors (IRs) and gustatory receptors (GRs), are involved in detection of volatile signals. In the present study, we aimed to identify candidate chemosensory receptors, and RNA-seq technology was employed to sequence the antennal transcriptome of *Protaetia brevitarsis* (Coleoptera: Scarabaeidae: Cetoniinae), a native agricultural and horticultural pest in East-Asia. According to the sequence similarity analysis, we identified 72 PbreORs, 11 PbreGRs and eight PbreIRs. Among PbreORs, PbreOR2, PbreOR33 and PbreOR53 were preliminarily classified into pheromone receptors. Further qRT-PCR analysis indicated that 11 PbreORs were specifically expressed in the antennae of male *P. brevitarsis*, whereas 23 PbreORs were specifically expressed in the female antennae. Our results laid a solid foundation for further functional elucidations of insect chemoreceptors, which could be used as the potential targets of pest management.

## Introduction

A sophisticated olfactory system is crucially important for survival and reproduction of most insects^1^. In insect antennae, the main olfactory organs in odorant recognition, there are several types of olfactory proteins, which are directly involved in the procedures of detection, recognition or degradation of peripheral odorants. These olfactory proteins include odorant-binding proteins (OBPs)^2,3^, chemosensory proteins (CSPs)^4,5^, odorant receptors (ORs), ionotropic receptors (IRs)^6^, gustatory receptors (GRs)^7^, sensory neuron membrane proteins (SNMPs)^8^ and odorant-degrading enzymes (ODEs)^9^. During the past decade, a convincing model for odorant detection of insects has been developed based on the in-depth studies of the function, structure and mechanism of olfactory proteins^1^.

Insect chemoreceptors (ORs, IRs and GRs), which are localized on the membrane surface of olfactory receptor neurons (ORNs), are activated by the odorants, generating electrical signals to the brain^10,11^. ORs, composed of approximate 400 amino acids (aa), contain seven transmembrane domains which can form binding sites for odorants^12^. Generally, ORs of insect include at least two major groups: pheromone receptors (PRs) associated with signal transduction of pheromone compounds, and general ORs which are considered to recognize host plant volatiles^13^. GRs mainly exist in the gustatory organs like mouthparts^7^. However, some of GRs are also identified from insect antennae, which are thought to participate in olfactory recognition^14^. IRs belong to another variant subfamily of ionotropic glutamate receptors (iGluRs)^15^. The study of insect chemoreceptors has become a potential way to develop novel pest control strategies.

The development of next-generation sequencing (NGS) technology has dramatically increased the screening efficiency of functional genes. In recent years, the entomological research also benefits from the improvement of NGS^16^. NGS has been widely used in identification of olfactory-related genes in insects. Up to now, olfactory-related genes of many insects in Coleoptera have been systematically studied, including species in Tenebrionidae, Curculionidae, Cerambycidae, Bothrideridae, Bostrychidae, Chrysomelidae and Scarabaeidae^17–31^. Like other insects, all coleopteran species, which are studied in odorant recognition, have three types of chemoreceptors (ORs, IRs and GRs). The white-spotted flower chafer, *Protaetia brevitarsis* (Coleoptera: Scarabaeidae: Cetoniinae), also published as *P. brevitaris* or *Potosia brevitarsis* in previous reports, is a native species in East-Asia, including Korean Peninsula, Japan, Thailand and all over of China^32–36^. The larvae of *P. brevitarsis* have not been documented as an underground pest, because their grubs are saprophytic, inhabiting in decaying plant tissue or fermented animal manure. However, *P. brevitarsis* adults are destructive to the flowers and fruits of corn, grape, peach, apple and pear, and they are considered as an agricultural and horticultural pest^32^. In China, the control of *P. brevitarsis* still relies on environmental-unfriendly agrochemicals, inefficient artificial-catching and trapping adults with mixture of sugar and acetic acid. Therefore, novel control strategies, such as chemical communication-based trapping and killing, have been urgently developed against *P. brevitarsis*. So far, the molecular mechanisms underlying olfactory recognition in *P. brevitarsis* remain largely unexplored, and even no information on its genome sequence is available.

To identify chemoreceptors of *P. brevitarsis*, we sequenced and analyzed antennal transcriptome of adult females and males using Illumina sequencing technology, and the expression levels of identified chemoreceptors at the mRNA level were determined using quantitative real-time PCR (qRT-PCR). Collectively, our findings could help better understand the mechanisms of olfactory recognition in *P. brevitarsis*, and provide a solid foundation for further study on relationship between olfactory-related genes and semiochemicals.

## Results

### Sequence analysis and unigene assembly

With Illumina HiSeq. 4000 platform, Solexa sequencing yielded a total of 21,055,915 and 22,384,914 clean reads for the antennal transcriptomes of females and males, respectively. Using the TRINITY (v2.4.0) *de novo* assembly program^16^, 70,267 unigenes were assembled with a mean length of 762.42 bp (N50 = 1,416 bp) (Table S1). The size distribution of unigenes revealed that 12,569 unigenes (17.89%) were longer than 500 bp and 13,067 unigenes (18.59%) were longer than 1,000 bp (Table S1). The raw reads of *P. brevitarsis* were deposited into the NCBI SRA database (accession number: SRR7057558 and SRR7057559).

### Identification of ORs

All putative OR genes were identified by a keyword search from the BLASTx annotation. In the present study, 72 ORs were identified from the antennal transcriptome of *P. brevitarsis*, which were named with a four-letter code (the first letter of the genus name followed by the first three letters of the species name) + OR + number based on the length of open reading frames (ORFs) (Table S2). Among all identified PbreORs, 42 PbreORs possessed complete ORFs, which were long gene models with encoded peptides ranging from 290 aa to 437 aa (Table S2). PbreOrco, sharing an aa sequence identity of ~92% with AcorOrco, was identified as a highly conserved co-receptor. However, the majority of other putative PbreORs (61 in 71 ORs) shared low identities (27~49%) with published coleopteran ORs in NCBI database (Table S2).

**Figure 1:**
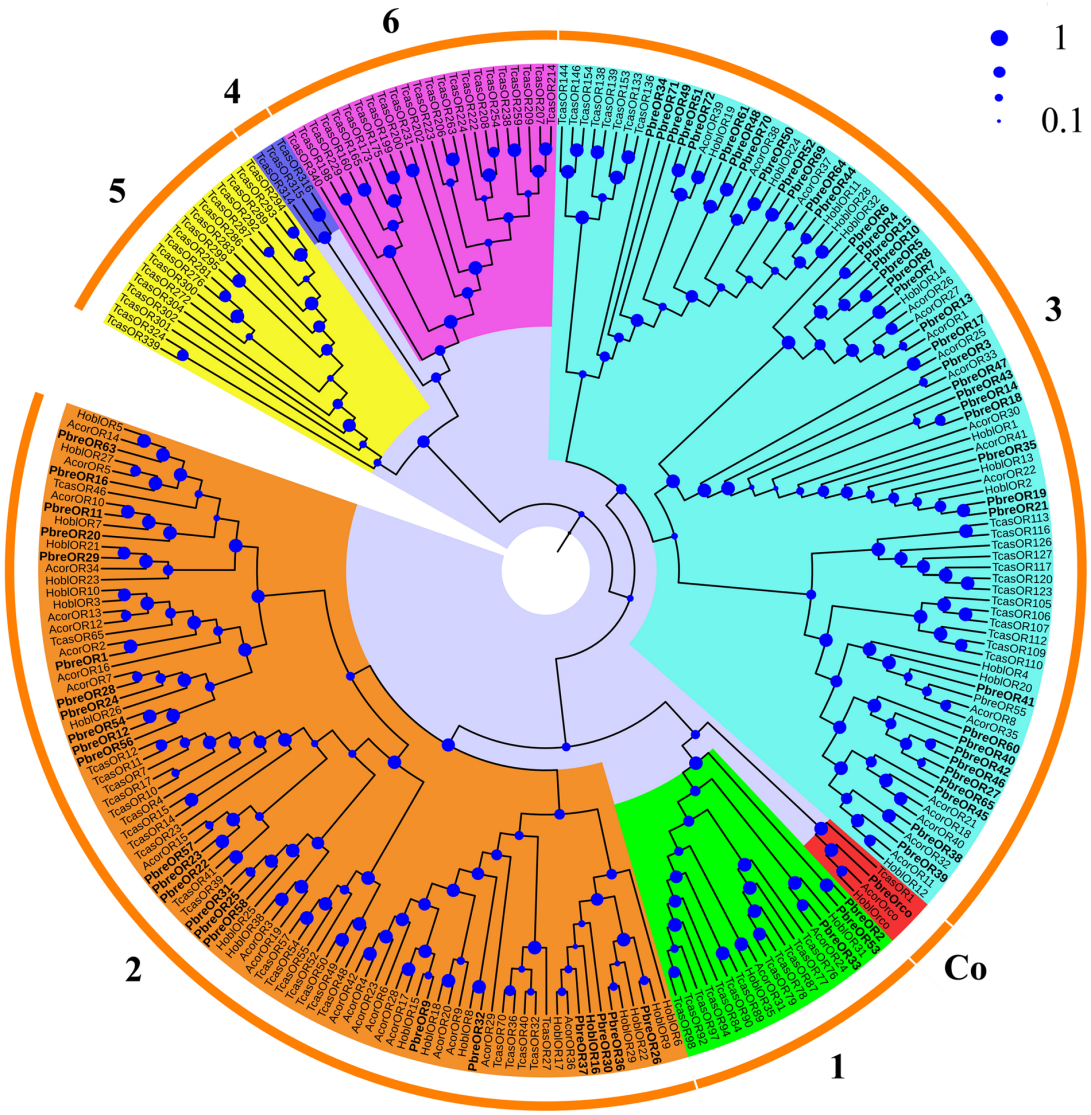
Neighbor-joining tree of candidate OR proteins from *Protaetia brevitarsis*, *Anomala corpulenta*, *Holotrichia oblita* and *T. castaneum*. The OR sub-families **1–7** are highlighted in different color shadings. PbreORs are labeled in bold. The size of blue spot at the nodes represents the bootstrap value.

To confirm the BLASTx annotation, a phylogenetic tree analysis was performed based on a neighbor-joining method. The phylogenetic tree, reconstructed with ORs from coleopteran species (*P. brevitarsis*, *Anomala corpulenta*, *Holotrichia oblita* and *Tribolium castaneum*), revealed that the candidate PbreORs were highly divergent among different coleopteran species (Fig. 1). As expected, PbreOrco, which was identified as a number of Orco family, was clustered with AcorOrco, HoblOrco and TcasOR1 (Orco) with high bootstrapping value (>98). All identified PbreORs were clustered into six subfamilies (Fig. 1), and the lineage subgroups were consistent with previous coleopteran studies^16,24,37,38^. The majority of putative PbreORs belonged to subfamily **3** and subfamily **2**, consisting of 40 (PbreOR3-8, 10, 13–15, 17–19, 21, 27, 34–35, 38–52, 60–61, 64–65, 69–72) and 23 PbreORs (PbreOR1, 9, 11–12, 16, 20, 22–26, 28–32, 36–37, 54, 56–58, 63), respectively. According to the previous reports about *A. corpulenta* and *H. oblita*^24,38^, members of subfamily **1** in coleopteran phylogenetic tree were classified as PRs. For *P. brevitarsis*, PbreOR2, PbreOR33 and PbreOR53 were grouped into subfamily **1**, indicating that they might play a role in recognizing pheromone compounds. From the phylogenetic tree of ORs, we also concluded that most PbreORs were clustered with ORs of *A. corpulenta* and *H. oblita* rather than those of *T. castaneum*, which was consistent with the closer genetic relationship among *P. brevitarsis*, *A. corpulenta* and *H. oblita*. However, no PbreOR was grouped into subfamilies **4–6**, so did *A. corpulenta*. The sequences of the 72 putative PbreORs were listed in the supplementary file.

**Figure 2:**
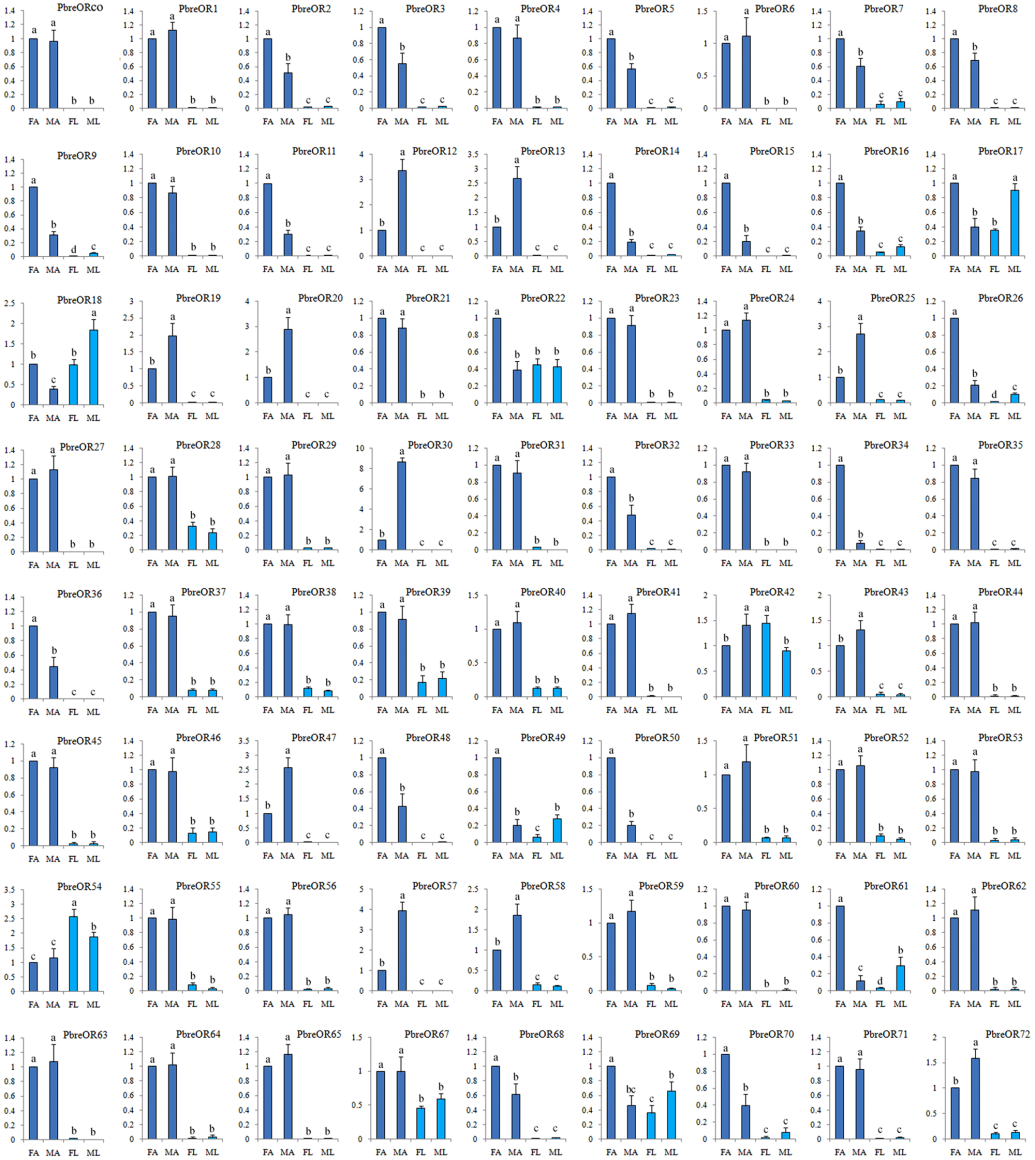
Transcript levels of *Protaetia brevitarsis* ORs in different antennae measured by qRT-PCR. FA: female antennae; MA: male antennae; FL: female legs; ML: male legs. DAPDH2 was used as an internal control. The different letters above each bar indicate significant differences (P < 0.05).

Based upon the transcriptom analysis, PbreOrco, as a co-receptor, was most abundant in *P. brevitarsis* antennae with an FPKM (fragments per kilobase of exon model per million mapped reads) value of 59.47 and 58.33 for females and males, respectively. However, other putative PbreORs showed low expression levels (FPKM values < 25.66). qRT-PCR was conducted to analyze the expression levels of all PbreOR genes in the antennae and legs of male and female beetles. The results indicated that 11 OR genes (PbreOR12-13, PbreOR19-20, PbreOR25, PbreOR30, PbreOR43, PbreOR47, PbreOR57-58 and PbreOR72) were significantly expressed in antennae of male beetles compared with those in females. Meanwhile, 23 OR genes (PbreOR2, PbreOR3, PbreOR5, PbreOR7-9, PbreOR11, PbreOR14-18, PbreOR22, PbreOR26, PbreOR32, PbreOR34, PbreOR36, PbreOR48-50, PbreOR61 and PbreOR68-70) showed female-specific expressions (Fig. 2). Additionally, eight OR genes (PbreOR17-18, PbreOR22, PbreOR42, PbreOR49, PbreOR54, PbreOR63 and PbreOR69) were highly expressed in the legs.

### Candidate GRs

Our bioinformatic analysis identified 11 candidate PbreGR-encoding transcripts from antennal transcriptome of *P. brevitarsis*, and they were numbered according to the length of ORF (Table S3). Among all identified PbreGRs, a complete ORF was identified in PbreGR1, PbreGR3 and PbreGR5, while other PbreGRs were annotated as partial sequences. The NCBI BLASTx results showed that PbreGR1, PbreGR2, PbreGR4 and PbreGR10 shared high aa identities (>68%) with GRs of *A. corpulenta*, a pest in the same superfamily.

**Figure 3:**
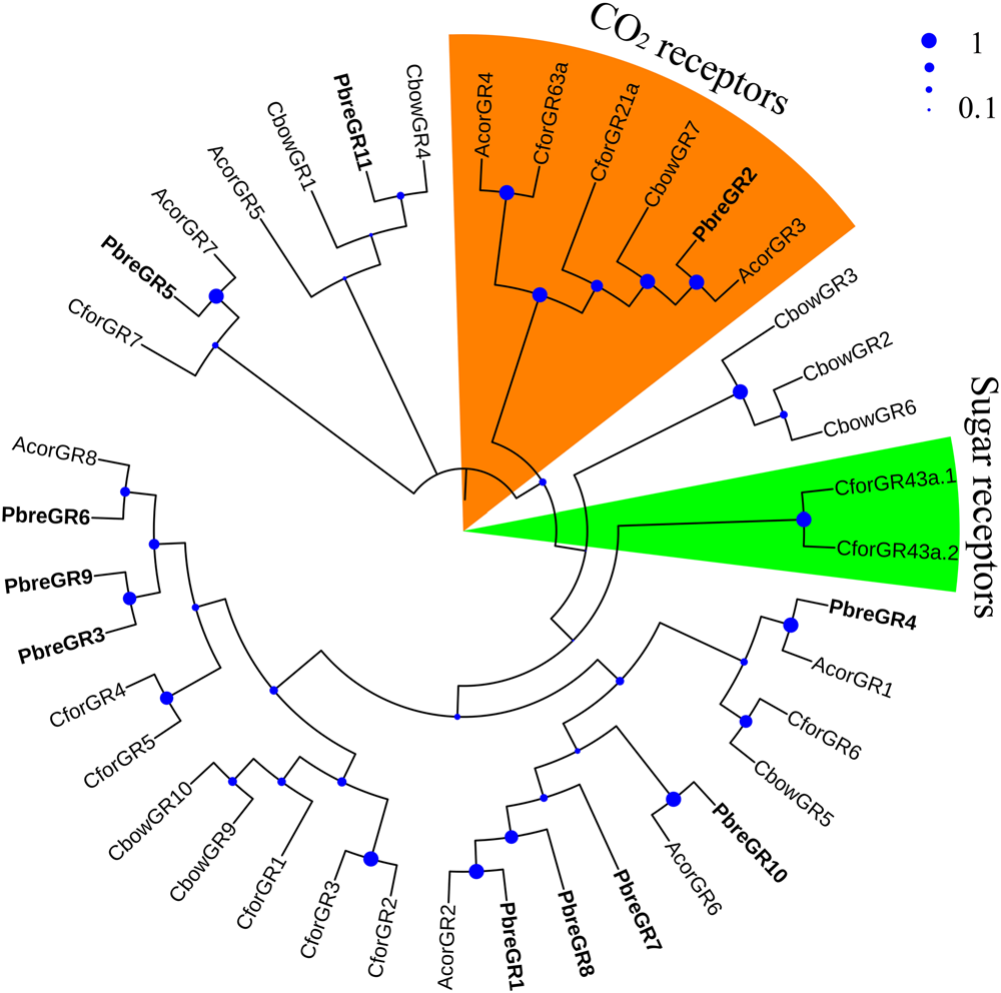
Neighbor-joining tree of candidate GR proteins from Protaetia brevitarsis, Anomala corpulenta, Cylas formicarius and Colaphellus bowringi.

The phylogenetic tree analysis indicated that the majority of PbreGRs were clustered with AcorGRs (Fig. 3). Especially, PbreGR1, PbreGR10, PbreGR4, PbreGR2 and PbreGR5 were clustered together with AcorGR2, AcorGR6, AcorGR1, AcorGR3 and AcorGR7 respectively, with the highest bootstrapp value (=100). The phylogenetic tree also showed that PbreGR2 was clustered into CO_2_ receptor subfamily, while no sugar receptor was found in antennae of *P. brevitarsis*.

**Figure 4:**
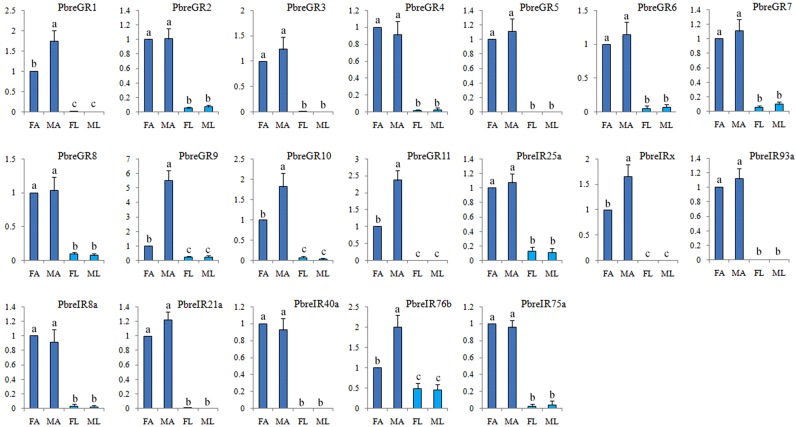
Transcript levels of *Protaetia brevitarsis* GRs and IRs in antennae measured by qRT-PCR. FA: female antennae; MA: male antennae; FL: female legs; ML: male legs. GAPDH2 was used as an internal control. The different letters above each bar indicate significant differences (P < 0.05).

All identified PbreGRs presented relatively low expression levels (FPKM < 18.72). The subsequent qRT-PCR analysis indicated that nearly all PbreGRs were highly expressed in the antennae compared with the legs. The analysis of sex differences showed that four PbreGRs (PbreGR1 and PbreGR9-11) displayed significant male antenna-specific expressions (Fig. 4).

### Candidate IRs

**Figure 5:**
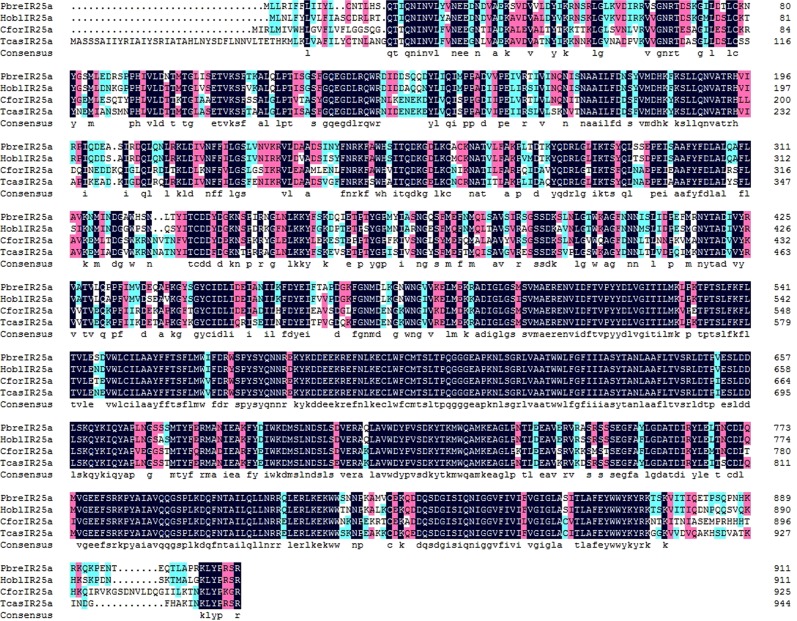
Amino acid multiple alignment of the putative PbreIR25a with *Cylas formicarius, Holotrichia oblita* and *Tribolium castaneum*. Blue shadings indicate the same sequence among species, while pink and green shading indicate 75% and 50% identity, respectively.

**Figure 6:**
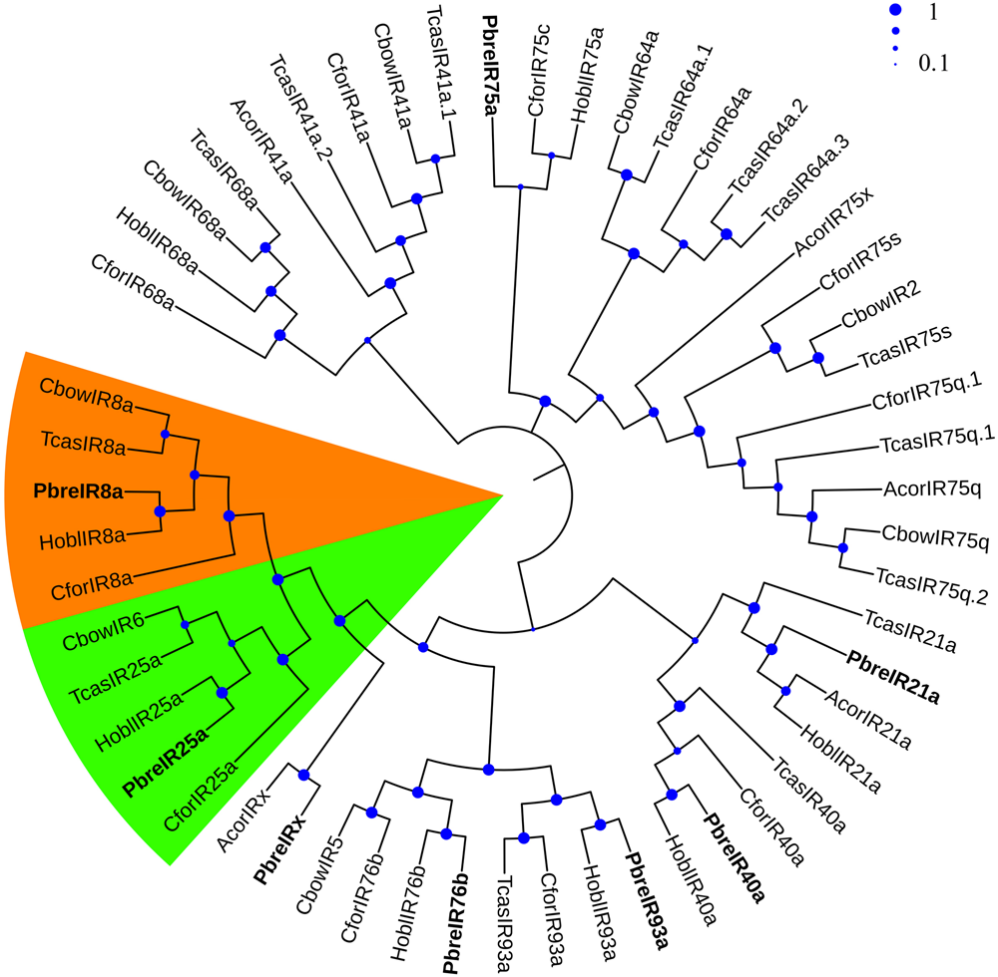
Neighbor-joining tree of candidate IR proteins from Protaetia brevitarsis, Anomala corpulenta, Cylas formicarius, Colaphellus bowringi, Holotrichia oblita and Tribolium castaneum.

A total of eight candidate PbreIRs were identified from the antennal transcriptome analysis. Of these IRs, seven PbreIRs had full-length ORFs with lengths ranging from 433 to 912 aa (Table S4). Multiple alignment analysis showed that the similarity of IR25a was 80.56% in *P. brevitarsis*, *T. castaneum*, *H. oblita* and *Cylas formicarius* (Fig. 5). According to the neighbor-joining tree of IRs from six coleopterans, we found that PbreIR25a, PbreIR93a, PbreIR8a, PbreIR21a, PbreIR40a, PbreIR76b and PbreIR75a were classified into IR25a, IR93a, IR8a, IR21a, IR40a, IR76b and IR75a lineages with high bootstrap values, respectively (Fig. 6). Meanwhile, the function of PbreIRx, which was clustered with AcorIRx (AKC58590.1), still remained unclassified or unknown. However, no orthologs for IR41a, IR68a, IR75s and IR75q were identified from *P. brevitarsis* antennae. The qRT-PCR analysis indicated that PbreIRx and PbreIR76b were significantly expressed in the male antennae compared with females (Fig. 4). Additionally, we found that PbreIR25a and PbreIR76b were expressed in male and female legs.

## Discussion

During the past decade, the molecular basis of chemoreception in various non-model organisms has been widely explored due to the development of NGS technology^1,39^. In the present study, we generated and analyzed the antennal transcriptome of *P. brevitarsis* using NGS technology. A total of 91 putative chemoreceptors encoding members of ORs, GRs, and IRs were identified. Identification of olfactory-related genes could provide insights into the molecular mechanisms of olfaction in *P. brevitarsis* and further study on potential molecular targets for pest control.

Chemoreceptors, functioning as heteromeric ion channels, are defined as key parts in insect olfactory system. However, compared with Lepidoptera, the understanding of chemosensory receptors in Coleoptera remains largely unexplored. To the best of our knowledge, the chemoreceptors have been systematically studied and reported from only ~20 species in coleopteran species, including those from Scarabaeidae, Cerambycidae, Chrysomelidae, Scolytidae, Curculionidae, Tenebrionidae and Brentidae (Table S5). Of these species, *A. corpulenta* and *H. oblita* have closer genetic relationship with *P. brevitarsis*, which belong to the superfamily of Scarabaeidae. The phylogenetic trees of ORs, GRs and IRs also confirmed their genetic homology.

In this study, we identified 72 candidate PbreORs from antennae of *P. brevitarsis*. The number of PbreORs was more than that of most coleopteran species, except for *T. castaneum* (341). Compared with the species in superfamily of Scarabaeidae, the number of PbreOR was significantly greater than that of *A. corpulenta* (43) and *H. oblita* (44) (Table S5). The difference in number of ORs might be attributed to the sequencing method or depth, or the process of sample preparation. Another reason, which caused the differences in the number of ORs expressed in antennae, could be the ecological/evolutionary differences across different species (Table S5).

From the 20 species, of which ORs have been reported, we selected *T. castaneum*, *A. corpulenta* and *H. oblita* to reconstruct the phylogenetic tree of ORs. *T. castaneum* has a relatively large olfactory capacity with 341 ORs^37^, and it is frequently used in reconstruction of phylogenetic tree. During the studies of ORs in *A. corpulenta* and *H. oblita*, the TcasORs are also used to reconstruct phylogenetic tree and employed as grouping basis of AcorORs and HoblORs^24,38^. In the present study, the subfamily of PbreORs was classified according to their cluster with ORs from *A. corpulenta* and *H. oblita*, the allied species of *P. brevitarsis*. Besides the Orco group, ORs of several coleopteran species (e.g. *Anoplophora glabripennis*, *Phyllotreta striolata*, *D. valens* and *Cylas formicarius*) have been classified into seven subfamilies^18,22,29,40^. However, ORs of some coleopteran species are not distributed in all seven subgroups. For example, ORs of *D. ponderosae* and *Rhynchophorus ferrugineus* are only classified into subfamilies **1**, **2** and **7**^17,41^. Moreover, no ORs of *Colaphellus bowringi*, *P. striolata* and *Brontispa longissima* are classified into subfamilies **4**, **5** and **6**^19,23,40^. In *P. brevitarsis*, except for PbreOrco, all PbreORs were classified into groups **1**, **2** and **3** according to the report about *A. corpulenta*, a species in Scarabaeidae. In fact, from *H. oblita*, another species in the same superfamily, several HoblORs (HoblOR28, 32, 40, 36, 43, 34, 24, 29, 16, 39, 22 and 9) are sorted into subgroups **4–7**^38^. However, the classification of AcorORs, which were clustered together with these HoblORs, was inconsistent with previous report^24^. Considering that most of these HoblORs had shorter length (<207aa) and non-full length status, we preferred to reference the results of *T. castaneum* and *A. corpulenta*.

Compared FPKM values with the qRT-PCR data, 66 of 72 PbreORs showed a general consistency across the two methods. Most of ORs in coleopteran species show similar trends of the expression in antennae of female and male^24,38^. In *P. brevitarsis*, six PbreORs (PbreOR2, PbreOR5, PbreOR7, PbreOR18, PbreOR19 and PbreOR59) showed significant differences between FPKM values and the qRT-PCR data. Such discrepancy could be attributed to the low expression values in the RNA-Seq set, or low sequencing depth. Considering the RNA-seq we conducted was non-replicated, qRT-PCR data might be more reliabe.

qRT-PCR analysis clearly revealed that most PbreOR genes were specifically expressed in antennae (Fig. 2). We also noticed that several PbreORs (PbreOR17-18, PbreOR22, PbreOR42, PbreOR49, PbreOR54, PbreOR63 and PbreOR69) were expressed in male or female legs, similar to the findings in *A. corpulenta*^24^. Unlike *T. castaneum*, in which Orco is simultaneously expressed in legs and other tissues^42^, the expression of PbreOrco was significantly antennae-specific (Fig. 2). In another coleopteran species, *A. chinensis*, several AchiORs are highly expressed in non-antennal tissues (AchiOR49 in the maxillary palps and female bodies)^43^. These non-antennae-specifically expressed ORs might play undiscovered functions.

GR family, which is usually abundant in the gustatory organs of insects^44^, functions in perceiving CO_2_, sugar, and other nutrients. In previous reports, GRs are also identified from antennae of coleopteran species (Table S5). According to the analysis of phylogenetic tree, we found that PbreGR2 was clustered into CO_2_ receptor subfamily (Fig. 3). Considering that the larvae of *P. brevitarsis* are coprophilous and saprophytic^34,35^, PbreGR2 was deduced to play a crucial function in locating oviposition sites by detecting CO_2_. Interestingly, no PbreGR was clustered with sugar receptors (CforGR43a.1 and CforGR 43a.2)^18^, though *P. brevitarsis* adults were fed on high-sugar fruits. We inferred that sugar receptors should be highly abundant in gustatory organs of adult *P. brevitarsis*.

As a conserved family of chemosensory receptors, IRs were also detected from coleopteran antennae (Table S5). We identified eight putative PbreIRs in the present study. Of these PbreIRs, PbreIR25a and PbreIR8a, like Orco to ORs, were speculated to function as co-receptors, and they were co-expressed along with other IRs^23^. The male-biased expressions of PbreIRx and PbreIR76b indicated that they might participate in the perception of sex pheromones.

Besides chemoreceptors, we also identified several OBPs, CSPs and ODEs from antennae of *P. brevitarsis* (unpublished data). These olfactory-related proteins also played crucial roles in olfactory recognition.

## Materials and Methods

### Insects and RNA extraction

*P. brevitarsis* larvae were reared on fermented wheat straw under constant conditions (28 ± 1 °C, 60 ± 5% RH). Newly-emerged adults were sorted by sex and fed with fresh apple. Antennae were excised from ~7-day-old unmated adults, immediately frozen in liquid nitrogen, and stored at −80 °C prior to further analysis.

The frozen antennae were ground to a fine powder by a pestle and mortar pre-chilled with liquid nitrogen. Total RNA was extracted from 100 antennae for each sex using the RNeasy Plus Mini Kit (Qiagen Co., Germany). Degradation and purity of RNA were determined by NanoPhotometer® spectrophotometer (IMPLEN, USA) and 1.0% agarose gels, respectively. RNA concentration was measured by a Qubit® RNA Assay Kit in a Qubit® 2.0 Fluorometer (Life Technologies, USA). The RNA integrity was assessed by the Bioanalyzer 2100 System (Agilent Technologies, USA).

### Construction of cDNA library and sequencing

The cDNA libraries of *P. brevitarsis* antennae were constructed following our previously reported procedure^45^, including purification of mRNA from 5 μg of total RNA, fragmentation of mRNA, generation of the first-strand cDNA, synthesis of the second-strand cDNA, cDNA end-repair and adenylation at the 3′ end, adapter ligation, cDNA fragment enrichment, PCR purification and amplification. Subsequently, the cDNA library was sequenced on Illumina HiSeq™ 4000 using 125 bp paired-end in a single run based on sequencing-by-synthesis method. Sequencing analysis was performed by Beijing Biomake Company (Beijing, China).

### Sequencing and assembly

After cluster generation on a cBot Cluster Generation System, the paired-end sequencing was performed on an Illumina Hiseq. 4000 platform. Subsequently, the raw reads were processed to generate clean reads. The reads with adapters were eliminated, the reads with >10% unknown bases (N) were discarded, and the low-quality sequences with Phred Quality Score Q < 20 bases were removed. The qualified reads were applied to generate non-redundant unigenes using short reads assembling program-Trinity (v2.4.0) with min_kmer_cov set to 2 by default and all other parameters set default values^16^. TransDecoder software (v5.0.0) (http://sourceforge.net/projects/transdecoder/) was used to predict whether the unigene sequences and their corresponding aa sequences were complete.

### Identification of *P. brevitarsis* chemoreceptors and phylogenetic analysis

The functions of assembled unigenes were annotated by searching these sequences against the Nr, Nt, KOG, KEGG, GO, Swiss-Prot and Pfam database using BlASTX program (E-value < 10^−5^)^45^. The sequences previously annotated as OR, GR and IR were manually verified by homology searches using BLAST (http://blast.ncbi.nlm.nih.gov/blast.cgi). To determine the expressions of OR, GR and IR genes, the reads for each chemoreceptor gene was converted to FPKM using RNA-Seq by Expectation-Maximization (RSEM v1.3.0, http://deweylab.github.io/RSEM/)^46^. FPKM values were calculated by the formula as follows:

$${\rm{FPKM}}=\frac{{\rm{cDNA}}\,{\rm{Fragments}}}{{\rm{Mapped}}\,{\rm{Fragments}}\,({\rm{Millions}})\times {\rm{Transcript}}\,{\rm{Length}}\,({\rm{kb}})}$$

The transmembrane domains of candidate ORs, GRs and IRs were predicted using the TMHMM server (v2.0, http://www.cbs.dtu.dk/services/TMHMM/). The alignments of candidate chemoreceptor genes were completed using ClustalX software (v2.1), and then the phylogenetic tree for the analysis of chemoreceptors was reconstructed by neighbor-joining method using MEGA5.0. Then, 1,000 bootstrap iterations were used to assess the reliability of nodes in phylogenetic tree^47^. In addition, Poisson correction method was applied to determine the evolutionary distances^48^. All non-intact sequences, with lengths shorter than the normal, were verified by multiple alignments to ensure that they were not part of other annotated chemosensory receptors.

### qRT-PCR

To quantify the expression levels of putative chemoreceptor genes in antennal transcriptomes of females and males, qRT-PCR was conducted on an ABI 7500 Fast Detection System using the SMART^TM^PCR cDNA synthesis kit (Clontech, Mountain View, USA). Total RNA was extracted from *P. brevitarsis* antennae and reversely transcribed into first-strand cDNA (500 ng RNA was inputted for each cDNA reaction), and then the newly synthesized cDNA was used as a template of qRT-PCR. In our pre-experiment, GAPDH2 performed better in terms of expression stability thanβ-actin in different tissues of *P. brevitarsis*. Therefore, GAPDH2, identified from the antennal transcriptome of *P. brevitarsis*, was selected as the reference gene to normalize the target gene expression and correct the variation between samples. Forward and reverse primers of GAPDH2 were as followed: 5′–GGCTTTTCGAGACGAACCGT–3′ and 5′– GCGGCTAAAGCTGTCGGAAA–3′. Gene-specific primers were designed using the Primer Premier (v 5.0). The primer efficiencies, evaluated by standard curve analysis, ranged from 93.8% to 107.1%. PCR reaction was conducted according to our previous report^45^. Briefly, PCR system consisted of 10.0 μL of 2 × SYBR Green PCR Master Mix, 0.4 μL of each primer, 2.0 μL of sample cDNA (100 ng·μL^−1^) and 7.2 μL sterilized ultrapure water. After an initial denaturation step at 95 °C for 3 min, amplifications were carried out with 40 cycles at a melting temperature of 95 °C for 10 s and an annealing temperature of 60 °C for 30 s. Before qRT-PCR analysis, a preliminary experiment was carried out using 10 random sequences to confirm that PCR products were our targets via sequencing the amplicons. Three technical replicates were performed for all qRT-PCR reactions. Relative expression levels of candidate chemoreceptor genes were determined using the comparative 2^−ΔΔCt^ method^49–52^. All data were normalized to the endogenous GAPDH2 level from the same individual samples. Three biological replicates were conducted in all tests.

Statistical analyses of gene expression among sexes and tissues were performed using one-way analysis of variance (ANOVA) followed by the Tukey’s test. The least significant significance was set at P < 0.05. SPSS 19.0 software was used in all statistical analyses.

## Conclusions

In order to understand the mechanism underlying olfactory recognition, we identified 91 putative chemosensory receptors from antennae of *P. brevitarsis*, including 72 PbreORs, 11 PbreGRs and eight PbreIRs. Our findings reinforced the knowledge on insect physiology and the molecular mechanisms of insect chemoreception. Moreover, our current data provided insights into the development of environmental-friendly pest control strategies.

## Supplementary information

Supplementary information

## Data Availability

The raw reads in this paper were available from the NCBI SRA database (accession number: SRR7057558 and SRR7057559).
