# Supplementary material for: Identification and expression of candidate chemosensory receptors in the white-spotted flower chafer, Protaetia brevitarsis
Source: Sci Rep. 2019 Mar 4;9:3339. doi: 10.1038/s41598-019-38896-x (PMC6399352; doi:10.1038/s41598-019-38896-x)
Supplement: Supplementary file 1 — Supplementary information [file 41598_2019_38896_MOESM1_ESM.pdf]

Identification and expression of candidate chemosensory receptors in the white-spotted flower chafer, *Protaetia brevitarsis*

Hongmin Liu[1,+], Xiaofang Zhang[2,+], Chunqin Liu[3], Yongqiang Liu[4], Xiangdong Mei[4], Tao Zhang[2,*]

1 College of Agronomy, Xinyang Agriculture and Forestry University, Xinyang, 464006, P. R. China

2 Institute of Plant Protection, Hebei Academy of Agriculture and Forestry Sciences/Integrated Pest Management Center of Hebei Province/Key Laboratory of IPM on Crops in Northern Region of North China, Ministry of Agriculture, Baoding, 071000, P. R. China

3 Cangzhou Technical College, Cangzhou, 061001, P. R. China

4 State Key Laboratory for Biology of Plant Diseases and Insect Pests, Institute of Plant Protection, Chinese Academy of Agricultural Sciences, Beijing 100193, P. R. China

[+] These authors contributed equally to this work.

* cauzht@163.com

Supplementary Table S1. An overview of the sequencing and assembly process

Length Range	Transcript	Unigene
200-300	27,168(25.72%)	24,461(34.81%)
300-500	25,814(24.44%)	20,170(28.70%)
500-1000	20,763(19.66%)	12,569(17.89%)
1000-2000	16,081(15.22%)	7,064(10.05%)
2000+	15,798(14.96%)	6,003(8.54%)
Total Number	105,624	70,267
Total length	110,479,623	53,572,904
N50 Length	2,050	1,416
Mean Length	1045.97	762.43

Supplementary Table S2. The Blastx matches of *Protaetia brevitarsis* candidate OR genes and their expressions in antennae

Gene ID	Gene name	Accession number	Full length	ORF (aa)	[1]TMD	Blastx annotation (Reference/Name/Species)	Score	*E*-value	Identity (%)	FPKM values		[2]Exp in male Ant
										Female	Male	
c68694.graph_c1	ORco	MH324899	N	288	4	AKC58535.1 | odorant co-receptor [*Anomala corpulenta*]	556	0.0	92	59.47	58.33	0.94
c62761.graph_c0	OR1	MH324834	N	454	7	AKC58537.1 | odorant receptor 2 [*Anomala corpulenta*]	450	6e-153	56	2.56	4.04	1.16
c68689.graph_c0	OR2	MH324835	Y	437	6	EFA05710.2 | odorant receptor 73 [*Tribolium castaneum*]	280	3e-84	38	20.14	19.7	0.53
c64214.graph_c0	OR3	MH324836	Y	431	5	AKC58557.1 | odorant receptor 22 [*Anomala corpulenta*]	455	1e-155	54	2.75	1.82	0.58
c64457.graph_c0	OR4	MH324837	Y	423	3	AKC58561.1 | odorant receptor 26 [*Anomala corpulenta]*	278	2e-86	37	4.82	2.79	0.83
c59458.graph_c0	OR5	MH324838	N	422	7	AKC58561.1 | odorant receptor 26 [*Anomala corpulenta*]	257	4e-78	35	1.74	2.43	0.56
c64985.graph_c0	OR6	MH324839	Y	422	2	AKC58561.1 | odorant receptor 26 [*Anomala corpulenta]*	251	9e-76	34	5.98	8.15	1.13
c61220.graph_c0	OR7	MH324840	Y	421	6	AKC58536.1 | odorant receptor 1 [*Anomala corpulenta*]	308	4e-98	38	1.89	2.3	0.60
c67157.graph_c0	OR8	MH324841	Y	420	6	AKC58536.1 | odorant receptor 1 [*Anomala corpulenta*]	244	4e-73	34	2.56	1.97	0.71
c61288.graph_c0	OR9	MH324842	Y	417	5	AKC58552.1 | odorant receptor 17 [*Anomala corpulenta*]	361	3e-119	45	3.04	1.93	0.32
c26789.graph_c0	OR10	MH324843	Y	416	5	AKC58561.1 | odorant receptor 26 [*Anomala corpulenta*]	270	3e-83	35	0.72	0.59	0.82
c57916.graph_c0	OR11	MH324844	N	416	6	AKC58545.1 | odorant receptor 10 [*Anomala corpulenta*]	496	3e-172	59	11.76	6.25	0.31
c66644.graph_c0	OR12	MH324845	Y	415	6	AKC58542.1 | odorant receptor 7 [*Anomala corpulenta*]	194	2e-54	30	8.96	40.22	3.34
c56664.graph_c0	OR13	MH324846	N	414	6	AKC58536.1 | odorant receptor 1 [*Anomala corpulenta*]	351	6e-115	39	1.3	3.28	2.65
c61018.graph_c0	OR14	MH324847	Y	414	7	AKC58557.1 | odorant receptor 22 [*Anomala corpulenta*]	454	1e-155	58	3.35	1.79	0.19
c67790.graph_c0	OR15	MH324848	N	413	4	AKC58536.1 | odorant receptor 1 [*Anomala corpulenta*]	275	2e-85	34	5.13	3.22	0.20
c64128.graph_c0	OR16	MH324849	Y	411	7	AKC58540.1 | odorant receptor 5 [*Anomala corpulenta*]	507	1e-176	60	4.59	3.47	0.34
c68297.graph_c0	OR17	MH324850	Y	411	4	AKC58560.1 | odorant receptor 25 [*Anomala corpulenta*]	398	1e-133	50	9.71	5.75	0.40
c59340.graph_c0	OR18	MH324851	Y	410	5	AKC58565.1 | odorant receptor 30 [*Anomala corpulenta*]	396	7e-133	48	1.09	3.22	0.38
c67207.graph_c0	OR19	MH324852	Y	410	4	AKC58557.1 | odorant receptor 22 [*Anomala corpulenta*]	625	0.0	72	5.9	5.4	1.99
c56159.graph_c0	OR20	MH324853	Y	409	4	AKC58545.1 | odorant receptor 10 [*Anomala corpulenta*]	330	8e-107	44	11.56	43.56	2.83
c61293.graph_c0	OR21	MH324854	Y	409	4	AKC58557.1 | odorant receptor 22 [*Anomala corpulenta*]	589	0.0	69	3.35	3.92	0.88
c37091.graph_c0	OR22	MH324855	Y	407	7	AKC58550.1 | odorant receptor 15 [*Anomala corpulenta*]	259	3e-79	33	0.34	.096	0.39
c58248.graph_c0	OR23	MH324856	Y	405	7	AKC58550.1 | odorant receptor 15 [*Anomala corpulenta*]	272	1e-84	35	3.29	2.85	0.89
c64529.graph_c0	OR24	MH324857	Y	405	6	AKC58542.1 | odorant receptor 7 [*Anomala corpulenta*]	326	1e-105	43	1.96	2.41	1.16
c57199.graph_c0	OR25	MH324858	Y	403	4	AJO62225.1 | olfactory receptor OR6 [*Tenebrio molitor*]	152	7e-39	29	0.47	1.41	2.73
c61940.graph_c0	OR26	MH324859	Y	403	7	AOG12953.1 | odorant receptor [*Eogystia hippophaecolus*]	108	2e-22	28	25.66	13.8	0.20
c60401.graph_c0	OR27	MH324860	Y	402	5	AKC58546.1 | odorant receptor 11 [*Anomala corpulenta*]	193	3e-54	39	2.75	3.03	1.14
c53451.graph_c0	OR28	MH324861	Y	401	7	AKC58542.1 | odorant receptor	348	4e-114	46	0.89	1.05	1.01

						7 [Anomala corpulenta]						
c61757.graph_c0	OR29	MH324862	Y	401	7	AKC58569.1 | odorant receptor 34 [*Anomala corpulenta*]	359	2e-119	49	3.67	4.38	1.01
c58428.graph_c0	OR30	MH324863	Y	399	6	APC94235.1 | odorant receptor 8 [*Pyrrhalta maculicollis*]	219	4e-64	34	0.75	3.53	8.64
c58360.graph_c0	OR31	MH324864	N	397	6	AKC58538.1 | odorant receptor 3 [*Anomala corpulenta*]	145	5e-36	30	3.24	2.83	0.88
c62170.graph_c0	OR32	MH324865	N	396	7	APC94315.1 | odorant receptor 8 [*Pyrrhalta aenescens*]	261	2e-80	37	7.61	4.52	0.45
c61817.graph_c0	OR33	MH324866	N	395	4	AKC58559.1 | odorant receptor 24 [*Anomala corpulenta*]	287	7e-91	39	6.47	6.48	0.91
c62818.graph_c0	OR34	MH324867	Y	394	5	AKC58573.1 | odorant receptor 38 [*Anomala corpulenta*]	119	1e-27	30	3.97	0.77	0.08
c58563.graph_c0	OR35	MH324868	N	392	4	AKC58557.1 | odorant receptor 22 [*Anomala corpulenta*]	503	4e-175	61	3.59	2.19	0.82
c59782.graph_c0	OR36	MH324869	N	392	7	AKC58571.1 | odorant receptor 36 [*Anomala corpulenta*]	123	6e-30	35	19.41	6.25	0.42
c65136.graph_c0	OR37	MH324870	Y	391	4	ALR72552.1 | odorant receptor OR7 [*Colaphellus bowringi*]	153	1e-36	28	2.74	2.29	0.97
c60035.graph_c0	OR38	MH324871	Y	383	6	AKC58546.1 | odorant receptor 11 [*Anomala corpulenta*]	239	3e-72	35	2.21	2.34	1.00
c52338.graph_c0	OR39	MH324872	Y	382	6	AKC58546.1 | odorant receptor 11 [*Anomala corpulenta*]	323	3e-105	43	1.13	0.76	0.91
c56969.graph_c0	OR40	MH324873	N	382	6	AKC58546.1 | odorant receptor 11 [*Anomala corpulenta*]	188	2e-52	30	2.35	2.91	1.10
c63529.graph_c0	OR41	MH324874	Y	381	6	AKC58543.1 | odorant receptor 8 [*Anomala corpulenta*]	376	1e-125	49	14.81	15.6	1.14
c58778.graph_c0	OR42	MH324875	Y	380	6	AKC58546.1 | odorant receptor 11 [*Anomala corpulenta*]	184	8e-51	32	2.75	5.55	1.41
c50321.graph_c0	OR43	MH324876	N	379	7	AKC58557.1 | odorant receptor 22 [*Anomala corpulenta*]	394	3e-132	51	0.42	1.64	1.32
c66216.graph_c0	OR44	MH324877	Y	377	6	AKC58572.1 | odorant receptor 37 [*Anomala corpulenta*]	207	1e-61	42	3.85	3.19	1.01
c61415.graph_c0	OR45	MH324878	N	376	5	AKC58556.1 | odorant receptor 21 [*Anomala corpulenta*]	219	1e-64	39	6.48	5.88	0.93
c63064.graph_c0	OR46	MH324879	N	376	7	AKC58546.1 | odorant receptor 11 [*Anomala corpulenta*]	183	8e-51	30	5.23	6.65	0.98
c62470.graph_c0	OR47	MH324880	Y	374	5	AKC58557.1 | odorant receptor 22 [*Anomala corpulenta*]	355	2e-117	48	5.32	13.77	2.56
c64270.graph_c0	OR48	MH324881	Y	373	7	AKC58572.1 | odorant receptor 37 [*Anomala corpulenta*]	173	2e-48	35	14.3	1.95	0.40
c66718.graph_c0	OR49	MH324882	N	373	3	AKC58572.1 | odorant receptor 37 [*Anomala corpulenta*]	158	9e-43	35	13.13	2.84	0.20
c66626.graph_c0	OR50	MH324883	Y	371	6	AKC58573.1 | odorant receptor 38 [*Anomala corpulenta*]	224	3e-68	45	16.38	4.24	0.20
c58159.graph_c0	OR51	MH324884	N	370	4	AKC58574.1 | odorant receptor 39 [*Anomala corpulenta*]	246	5e-77	49	1.2	1.3	1.21
c53105.graph_c0	OR52	MH324885	N	366	6	AKC58573 | odorant receptor 38 [*Anomala corpulenta*]	182	4e-52	40	1.79	1.31	1.04
c63434.graph_c0	OR53	MH324886	N	364	6	EFA05710.2 | odorant receptor 73 [*Tribolium castaneum*]	229	7e-66	37	6.11	6.53	0.97
c61280.graph_c0	OR54	MH324887	N	356	6	AKC58542.1 | odorant receptor 7 [*Anomala corpulenta*]	142	2e-35	27	2.69	5.11	1.20
c66300.graph_c0	OR55	MH324888	N	353	4	AKC58543.1 | odorant receptor 8 [*Anomala corpulenta*]	377	2e-126	53	7.09	7.2	0.99
c64771.graph_c0	OR56	MH324889	N	349	5	AKC58547.1 | odorant receptor 12 [*Anomala corpulenta*]	166	6e-44	31	7.37	7.34	1.03
c67873.graph_c1	OR57	MH324890	N	349	6	AKC58550.1 | odorant receptor 15 [*Anomala corpulenta*]	211	2e-61	33	8.97	37.7	3.92
c60655.graph_c0	OR58	MH324891	N	344	6	ARO76449.1 | odorant receptor 44 [*Conogethes punctiferalis*]	84.7	1e-14	22	1.62	3.5	1.81
c55069.graph_c0	OR59	MH324892	N	341	5	AIR07412.1 | odorant receptor 21a [*Aedes albopictus*]	228	7e-70	62	1.34	3.69	1.22
c61932.graph_c0	OR60	MH324893	N	341	6	AKC58546.1 | odorant receptor 11 [*Anomala corpulenta*]	113	7e-25	33	3.07	3.32	1.96
c64992.graph_c0	OR61	MH324894	N	333	4	AKC58572.1 | odorant receptor 37 [*Anomala corpulenta*]	177	2e-50	34	7.94	1.37	0.11

c63184.graph_c0	OR62	MH324895	N	329	5	XP_017777946.1 | odorant receptor 30a-like [*Nicrophorus vespilloides*]	151	4e-39	33	4.56	3.19	1.09
c60530.graph_c0	OR63	MH324896	N	316	6	AKC58549.1 | odorant receptor 14 [*Anomala corpulenta*]	456	4e-158	69	8.85	7.11	1.18
c63350.graph_c1	OR64	MH324897	N	316	6	AKC58572.1 | odorant receptor 37 [*Anomala corpulenta*]	176	2e-50	41	3.47	3.95	1.01
c65645.graph_c0	OR65	MH324898	N	290	4	AKC58546.1 | odorant receptor 11 [*Anomala corpulenta*]	199	3e-58	38	7.28	8.03	1.17
c58483.graph_c0	OR67	MH324900	N	262	3	AKC58537.1 | odorant receptor 2 [*Anomala corpulenta*]	114	1e-25	33	1.17	1.81	1.00
c64264.graph_c0	OR68	MH324901	N	245	4	AKC58564.1 | odorant receptor 29 [*Anomala corpulenta*]	130	4e-32	31	7.73	1.86	0.61
c54545.graph_c0	OR69	MH324902	N	238	4	AKC58573.1 | odorant receptor 38 [*Anomala corpulenta*]	169	1e-48	39	4.02	1.98	0.45
c63892.graph_c0	OR70	MH324903	N	211	3	AKC58572.1 | odorant receptor 37 [*Anomala corpulenta*]	144	3e-39	38	6.68	0.73	0.39
c56257.graph_c0	OR71	MH324904	N	203	4	AKC58572.1 | odorant receptor 37 [*Anomala corpulenta*]	90.1	2e-18	30	0.66	0.31	0.98
c93841.graph_c0	OR72	MH324905	N	182	1	AKC58574.1 | odorant receptor 39 [*Anomala corpulenta*]	168	4e-49	47	0.15	0.29	1.59

[1]TMD-transmembrane domains

[2] Exp in male Ant- relatively expression levels in male antennae compared with that in female antennae, tested in qRT-PCR using the comparative $2^{-\Delta\Delta Ct}$ method.

Supplementary Table S3. The Blastx matches of *Protaetia brevitarsis* candidate GR genes.

Gene ID	Gene name	Accession number	Full length	ORF (aa)	TMD*	Blastx annotation (Reference/Name/Species)	Score	*E*-value	Identity (%)	FPKM values	
										Female	Male
c65699.graph_c0	GR1	MH324906	Y	440	4	AKC58579.1 | gustatory receptor 2 [*Anomala corpulenta*]	566	0.0	68	18.72	13.08
c58251.graph_c0	GR2	MH324907	N	439	6	AKC58580.2 | gustatory receptor 3 [*Anomala corpulenta*]	783	0.0	83	1.29	1.35
c62976.graph_c1	GR3	MH324908	Y	403	7	XP_017776024.1 | putative gustatory receptor 28b [*Nicrophorus vespilloides*]	142	1e-34	29	8.06	9.94
c60730.graph_c0	GR4	MH324909	N	396	7	AKC58578.1 | gustatory receptor 1 [*Anomala corpulenta*]	510	2e-179	77	2.22	0.78
c59477.graph_c0	GR5	MH324910	Y	249	4	XP_021926063.1 | gustatory receptor for sugar taste 64f-like isoform X2 [*Zootermopsis nevadensis*]	112	3e-25	32	4.24	4.61
c44608.graph_c1	GR6	MH324911	N	216	3	AKC58585.1 | gustatory receptor 8 [*Anomala corpulenta*]	72.8	5e-12	31	0.58	0.33
c65250.graph_c0	GR7	MH324912	N	190	3	ALG36126.1 | gustatory receptor 2 [*Sclerodermus sp.* MQW-2015]	91.3	3e-19	38	2.83	2.61
c2730.graph_c0	GR8	MH324913	N	148	3	AKC58579.1 | gustatory receptor 2 [*Anomala corpulenta*]	82.8	1e-15	32	0.37	0.24
c79372.graph_c0	GR9	MH324914	N	131	2	CAL23187.2 | gustatory receptor candidate 54 [*Tribolium castaneum*]	49.7	2e-04	37	0	0.48
c36602.graph_c0	GR10	MH324915	N	124	1	AKC58583.1 | gustatory receptor 6 [*Anomala corpulenta*]	168	2e-50	73	0.22	0.42
c115661.graph_c0	GR11	MH324916	N	111	2	NP_001137601.1| gustatory receptor [*Tribolium castaneum*]	68.6	3e-11	44	0	0.32

*TMD-transmembrane domains

Supplementary Table S4. The Blastx matches of *Protaetia brevitarsis* candidate IR genes.

Gene ID	Gene name	Accession number	Full length	ORF (aa)	TMD*	Blastx annotation (Reference/Name/Species)	Score	*E*-value	Identity (%)	FPKM values	
										Female	Male
c66084.graph_c0	IR25a	MH324917	Y	912	3	AVH87315.1 | ionotropic receptor 27 [*Holotrichia parallela*]	1670	0.0	87	48.04	47.37
c66301.graph_c0	IRx	MH324918	Y	897	3	AUF73087.1 | ionotropic receptor [*Anoplophora chinensis*]	846	0.0	50	1.53	3.55
c55215.graph_c0	IR93a	MH324919	Y	852	4	AVH87302.1 | ionotropic receptor 14 [*Holotrichia parallela*]	1153	0.0	67	108.55	106.02
c64765.graph_c0	IR8a	MH324920	Y	756	2	AVH87289.1 | ionotropic receptor 1 [*Holotrichia parallela*]	1116	0.0	72	33.21	31.98
c57624.graph_c0	IR21a	MH324921	N	722	4	AKC58586.1 | chemosensory ionotropic receptor 21a [*Anomala corpulenta*]	793	0.0	58	223.06	196.78
c61541.graph_c0	IR40a	MH324922	Y	706	3	XP_023310509.1 | ionotropic receptor 40a [*Anoplophora glabripennis*]	764	0.0	57	4.81	3.91
c56110.graph_c0	IR76b	MH324923	Y	540	4	AVH87308.1 | ionotropic receptor 20 [*Holotrichia parallela*]	827	0.0	72	63.78	57.08
c64795.graph_c0	IR75a	MH324925	Y	433	5	AVH87296.1 | ionotropic receptor 8 [*Holotrichia parallela*]	155	8e-42	43	12.97	11.87

*TMD-transmembrane domains

Supplementary Table S5. The number of chemoreceptors in coleopteran species identified from transcriptome analysis.

Species	Family	ORs	GRs	IRs	References
Anomala corpulenta	Scarabaeidae	43	8	5	24
Holotrichia oblita	Scarabaeidae	44	-	9	50
Saperda populnea	Cerambycidae	56	-	24	51
Anoplophora glabripennis	Cerambycidae	37	11	-	52
Anoplophora chinensis	Cerambycidae	53	17	4	43
Megacyllene caryae	Cerambycidae	57	-	-	28
Monochamus alternatus	Cerambycidae	10	2	8	30
Brontispa longissima	Chrysomelidae	48	-	19	19
Phyllotreta striolata	Chrysomelidae	73	36	49	40
Colaphellus bowringi	Chrysomelidae	43	10	9	23
Ambrostoma quadriimpressum	Chrysomelidae	34	-	20	31
Tomicus yunnanensis	Scolytidae	9	8	3	27
Ips typographus	Scolytidae	43	7	6	17
Dendroctonus ponderosae	Scolytidae	49	2	15	17
Dendroctonus valens	Scolytinae	22	4	3	22
Rhynchophorus ferrugineus	Curculionidae	76	15	10	41
Tenebrio molitor	Tenebrionidae	20	-	6	25
Cylas formicarius	Brentidae	54	15	11	18
Rhyzopertha dominica	Bostrichidae	6	-	8	21
Protaetia. brevitarsis	Scarabaeidae	72	11	8	This study

Supplementary Table S6. Primers used for fluorescence quantitative real-time PCR.

Name	Forward primer (5'-3')	Reverse primer (3'-5')
PbreOrco	ATCTTCGGCAATCGGCTCATCG	CGGATATCGACATGGCCTTCTGAC
PbreOR1	TGTCCCAGACCGCAGGAATA	AGTAATACCGCCCCTGTCGT
PbreOR2	TGGAGCACGCTATATCGCAACTG	AGCAAGCTGATCACCACTGAAGTC
PbreOR3	AAGCATCTGCTGTTGGCCTGAC	GCAAGAGCCTCTATGGTTGTGGTG
PbreOR4	GCGAGGATGCTGTTTCTGCT	GCCGGAAGCACATCAGTCAA
PbreOR5	AAGTATTCGTTGGTCCTCGCTCAG	CTGGAACACCTCCGCCAATGTATC
PbreOR6	GCCATCAGAGTTGTCGGTAGCG	GCCTTATCACTGCGTCCTCTTGAC
PbreOR7	TCAACTGGTCAAAGACGCCG	ATTGTGCAGTGCAATTGGCG
PbreOR8	CCTACGTATCAGATGCTGCCTTCG	TCATGATGACTGGCCGTTGACTAC
PbreOR9	CACCTCTACCGCCTTCTTGTTCC	ATCGGTTCTGTTAGGCGTTCTTCC
PbreOR10	CTATCTCAGCGATTGGAGCACCTG	CGCATCACCATCAAGAATGACACG
PbreOR11	ACGTGCGTTGACCGAAGATGG	TGGTACACAGAGCTTGAACGAAGG
PbreOR12	AGGTGAACAGAGGCGGTGATATTG	AAGTTACGGCAGCGGTGAATAGG
PbreOR13	GAGTACCTGCGTGTCCGAAGC	TCTGCGACCTGGTCATTATCATGC
PbreOR14	CGTTGGTGACGATTTGCTGC	ACCGGCTGGTCCGAAGATAA
PbreOR15	CTTCTGCATCAATTGCGCTGGTC	TCTGATGGTGTTCCGCACATATCC
PbreOR16	TTTGGTTTCCTCGTTGCCGA	ACTGGTCGATGACCACTTGC
PbreOR17	GAGATGTTCACCTTCGCCGTAGC	AATCGTCAGAATCCTGCCAGTTGC
PbreOR18	TAGCCGATGCGATCTATGAATGCG	ACCGCAGTCAAGCATATCTTCCTC
PbreOR19	ACGTTCGGAATAGAGGCATGGTG	GCATGATGACGAACTGCACAGC
PbreOR20	ATATGCCCATCGGCAACGTC	CAACGCTGTATCGCACTGGA
PbreOR21	ATGCTGATGTATGCTGCTGCTCAC	GGCTAGGCTTAGGTGACCACTCTC
PbreOR22	ATACGACACGCCAAGAAGTGGTTG	TCATCGCCTACCGGATACCAACC
PbreOR23	TGTTCGGGTATTGGCAACCA	AGGTGCGTTACTGGCGAATG
PbreOR24	TGTGCGGTCAGCCACATATT	ACGGTAGTCATCCTGCCCAA
PbreOR25	GTTATTCGCCGGTGCCTGCTAG	TCGCCAATGCTTCATCCAACTCG
PbreOR26	TGGGCATTTGCTTGCGACAT	CCAGACTCCTGCATCCCACT
PbreOR27	CAGTCGGAGAGTGTTGGTGATGC	ACAGAACGTCGGCAGATTAAGCAC

PbreOR28	TGGGCCTTACCTACGGCATAA	CAGCAGTAAGCACCACTGGC
PbreOR29	ACATGGCGACTTATCGGCTT	TAGGCACTGACGTAGCCACA
PbreOR30	GACCTCTTGCGATAACCGCC	TTAGCCAGGCGTTGCAGAAG
PbreOR31	TCGCTACGCTGATGACACAACTAC	ACCAGGCCAGTCCATTGAATAAGC
PbreOR32	AGAGTGGCGGTATCGGTCAA	ACCGCCAGGTAGGAATACGA
PbreOR33	GTTACAGATCCGGCAGGCAC	GATTGCCGGCAGTTGTTTCG
PbreOR34	TCGCTTCTGCTTGCAGTGAGTTAC	GACTGCTTGTGAGAATTGCGACAC
PbreOR35	ATGGAGGGCTTGGTGAGGAT	AGAGAGGTCAGTGCGATGGA
PbreOR36	GTACGCTACGGGAATGTGCT	GCCTGGTTCCCTCAGCAAAG
PbreOR37	TTGCAGTCGTTCCTGTACCATTGG	CTCGACGCCGTCATCACTATCAAC
PbreOR38	TTGCCAATGACTTGCCTGCT	CTGAGTGGCCAGCGCAATTA
PbreOR39	AGCTTGCTACCTTGGTAGTTACGC	TCATCGTGAACGTGAACCTCTGC
PbreOR40	TGCCGAGTGCCTGGTACGAC	TGCCGAGTGCCTGGTACGAC
PbreOR41	GAGACCGCTGCTCCGTCATTC	GCCTGAAGTACCACGTAGAAGCC
PbreOR42	AGGCCTGTTTAGTCCTGCGA	CCCTGAGAAACAGCAGCACC
PbreOR43	AGCGTGGCTGCAACCTTCAAC	CTGGTCTCAGTATCGCTTGTGCTG
PbreOR44	GGCAACGTTCTTCAGCGAGT	CGACTCACCAGTGACCACGA
PbreOR45	AGGCTGAAGATGAGGCGTCGATAG	GCCGCATCTGCTACACTTGAAGTC
PbreOR46	TGTGGAGGATACAGATTCGATGCG	CGTATATCGCACGGCGTGTACC
PbreOR47	TGACTTGGAGACAGCGTTGC	TGGCAGCTTGGCTGTAATCG
PbreOR48	TGGTGATGCTTGCAGACGTAGTTC	GCACGAACGCATCCATAACAACAG
PbreOR49	TGTGGTTCTGGTCTTCTTGCTGAG	GTCATCACGACCGCTGGTACAAG
PbreOR50	AATCATGGGCGAACGGATGC	CTTCGCGTAATCAGCGCCTA
PbreOR51	CGATGCGTTATTCTTGGCGATCTG	TCACCAACAGCAGCTTCGTAGATC
PbreOR52	ACTGCATGCACTGGCATCTT	TGCTATCATTTCAGCCGGGA
PbreOR53	GATTCCGTGCGGAGTCATAGTGC	CGACCACCAACACCGCCATG
PbreOR54	GAAGGAGACGATAAGGACGAAGGC	CTGTTCTGCCAGCTCCAATACTCG
PbreOR55	AGCAAGAATATGTCCTCGCATCCG	GCTCTTATTCGTTGCTGCCATGTG
PbreOR56	CGAAGTCACTTTGCAAGCCG	TGCGAGCTGTGACGAGAATG
PbreOR57	GCGTCGATCTTCACCTGGACATTG	GCACCGGCTATTGGCAGTGATC

PbreOR58	TGGCTGCAGCGATAAACACA	TATTACTCCGTGCGCTCCCA
PbreOR59	GTGCTTGTCGTCCTGGTAGG	CCTCCCTAGCTACACCCCAG
PbreOR60	CTTCGTGCGTGAAGCATCACAATC	AAGATGGCGTGGAACCAGTTGC
PbreOR61	AGGCTAAGCATTTCTGGCGT	ATCAACCCTACCGCCATTGT
PbreOR62	TTGGAGCGTTTCGCTTCAGG	CTCCATGCAGCCGTGGAATA
PbreOR63	AGTGGCTGGCATTTGGGTTC	AGTCATGCCCATGTTGACGTT
PbreOR64	CGCATGGAAGTGCTGAGAGG	CCCATGACTGCCATGTAGGC
PbreOR65	TCGTCGCGTCCTTATGCACATTG	TGCGCCGATGCCAAGTAATACG
PbreOR67	TTACTGAAGCGTCACTTGGAACCG	AGCGTACAGTCCTCTCCAGCAG
PbreOR68	TGAGCCGCAGATGGAAGAGT	CCCACGCGCTTCTATCCAAC
PbreOR69	TTCCCGGCTGAAATGGTTGC	ATGATTGGTGCTGCGACCTG
PbreOR70	TTTGTATCAGTCGCCGGCAT	GGGCCTCTGACATTTGACCAT
PbreOR71	CGTAAAGCTGTCTGGAGCCG	GTGACAGCAGTACCAGAGCAC
PbreOR72	ACCTTGCTGGTGGTATTCACGATC	AGGCATCATGCGTCATTACAGTCG
PbreIR25a	ATCCCAAAGCGATGGTGTGC	TGGGTTGCGATGGAGTCTCT
PbreIRx	AACGAGAGCTTAGCGAGGGT	CCCATCGCATCCAGTTGCTT
PbreIR93a	TAATACGTGGCTGTGCCTGC	ACAAACCACCTCTCGCACTG
PbreIR8a	ACAGCGAGGTGAGACAGACT	CGCTCCAACGATACTGAGCC
PbreIR21a	ATTCCTATGATTGCGCCGCC	AGGTCAACCAGACCGTCCAA
PbreIR40a	ACATTAGCGGACAGTGGTGC	CCGGCCAAACCTTCCAATGA
PbreIR76b	GCCTATCGCCAGTGTGTCAG	TTCCAACGCGGCTTCTATGG
PbreIR75a	TTTAGCGGACTTACGGAAAGCA	TCTTGTCGCTGGTTCAGTGT
PbreGR1	AACCAAACAGCGTCCTCTGC	ATTTCGACGGCACCAAGGTC
PbreGR2	ACAACGCTTTGGAATCGGCT	CCGCTGTACGCTTTGCCTAA
PbreGR3	GGTCACTGATGGCGTTGTGT	CTTCACGCAGTTCCCTGTCA
PbreGR4	AGTTTCGGCGTCATGTTGCT	CCGCACAGTTCCTTCACGTT
PbreGR5	TGGTACATGGGCACATTCGTT	CTGACCTGTCTAGCTGCACG
PbreGR6	CGTTTGAAGCCCGTAGGACC	AGAGGCATCGCTTCTGGTCT
PbreGR7	TGCCGTCGGTTGAAAGACTA	TCCTGACGGATCCCTGATTGT
PbreGR8	AAGCGTTTTCGCTTCAAGTGTT	GCAAGTGTGACAGTTTCCTTGA

PbreGR9	GTGCACTGGACTGGAAAGCA	TCTTCCGTGGTATCATCGGCT
PbreGR10	CACGACAAATTGGAATTCACAGCA	CGCCTTCATGACAGCGTATCT
PbreGR11	CGCGCTAGTGCTAGTGGAAT	GCTGCACTTTCATCAAACCGT
GAPDH	GGCTTTTCGAGACGAACCGT	GCGGCTAAAGCTGTCGGAAA

Sequences of ORs from coleopteran species used to reconstruct phylogenetic tree

>PbreOrco
AMSGVPYYISLVYQIYYVGFSMLHSNLLDSLFCSWLIFACEQLQHLKEIMKPLMELSATLDTYVPKSADLF
RAPSAASRDNLIDNDYNQRNEEATMKGFYTTSQEMGVTYRSGNIQDFSGGIGPNGLTKKQELMVRSAIK
YWVERHKHVVRLVTAIGDAYGIALLLHMLTSTITLTLLAYQATKIDGVNKYALTVLGYLIYALAQVFHFC
IFGNRLIEESSSVMEAAYSCHWYDGSEEAKTFVQIVCQQCQKAMSISGAKFFTISLDLFASVLGATVTYFM
VLVQLK
>PbreOR1
SSQRVLKDYFKVQILFFKMIGVNLENSDNSFKSRVYFVYSQVILILTVYLFFITECVDLIGKWGDIDNMTFN
LCYLLSQTAGICKIVAIMLRREKVKKMLKILESDYFLPNYDRGGITEYRIIKEAVRQINVQTYFLNTIVCLIV
ANRGFFAGFDKGYFEPTDDFSGNGTVKHYQLLPYTTWFPFDTNITPFYEIAFIYQIAGALMGGLVIGTSDSF
ICGLMVHVKAQLRILKNNLKNHVKKAIREKEINTDINKQVPNITLYDTKKLLPEVQALIKHYLGECIIHHQ
KIIHLAERVESDFSYIMLIQFLTSLGLLCLSMFQISNNNVKSTGFLSMACFSFMMFYQLFMFCWNGNGVLE
LSLDIVFAAYDSEWFLCDLEIQKAIISIIQRAQRPLQISAGKFAFLTLETYMNVINFIIFLLDETIYRFYELRDR
IIWFYKTLINNFTKYTNVF
>PbreOR2
MYVRWLVITHPDVFIPYACIMKLERIKRVVVSQSNVPVSSVSGGRKMSEKIVHQFMRVNVKILEITELWL
EDGNLTAKKMKILKLCIMFILVCPTCIPLLREFIIVLIDPAPDIVHRIQIILAESCVLGAMYMVICFWKNRSQI
KDLVDSIADFANYSELDLLIIDEKAGFFSKIVLGYSLSGMVIYTLSPLLSTESCEKVKSQYKIDHGIPCGIIVP
IRIPIDPENSHIFRFYVFDQVVNGVLAVLVVVNITMMVCGLVEHAISQLKQVRCLMSKLSESGNIEEKLGF
VVKYHYAVIRFINNINKYFGSQLVLHFTLTSVVISLLGFQILMVNDRKESLMYALHLIGWLIMLYNICYYG
QLLIDESIGVAFDAYSVPWYNCSVRVQKDIKFIVMRSQKPLTLKAMNLGIMSHPTFLGVISSSYSYFTLLLK
VKNA
>PbreOR3
MKMPVEIRMRKLSFGKTFLVKIIKLSKYYLISLSDFSKDLYKDGVKRTILPAKIILQSICCWPDSDTAFALFV
GWLLFANLFLILIFHAAYVFKHFNDIGDTIAVGVTVTTTIEALARLYLIITRRRIINSTLVMVWKQFWPVNA
LETKATQRIERSAQTVVILATMLLILASSCNCFLSGVPLVRYHDIIFKSAFPFDWEMMYVYEFIYVWQYFIS
WFEVLLVCGFDFFFTTLVSICALQFTIMQEIFKSILTRKSKKQRMAIFGERGRKMTDREMLFECLEQHKLLI
GICNDLEKSFNTQILLQFFVSTCAICAPSLVLKIDSSQFFKTMTYAVGHLCQLYNYCHVGATLSHESVLLSQ
AIYESNWHLTHDIQFQKALLIMLQRSQNAQCLTAVGVTEMNFESFIKILRLSFSFYTLLNNLLMKNMNV
>PbreOR4
MMVFRKNVQRMLNRFQLFQDKVHFLNDEAKYAMVLGRLLVSIANLSPMDYNLARMLFLLFTVAVSIIH
EITIAVCFASYVRDVDTLTDVLPALSLGIQIVSKGTVLLIKTKQLATLTEVVWNDFWPANIMGHAVEYKIS
KLSKRILIAFYTELIVTIVYSISTVILPLRRTPKVLFQQASYPLDLSKPLGYSIAFCIQSYMTLYAETATICGYD
LMYQAICINCAAQFRLLCVALEYIGTGKEKEILEEIQNAVSDKTKRKSIKDQDANKELLGICVKHHQTLIN
VGHELNKIFGTGHFLQLCATLTGICTISYRISQTNDYNTIFVGLNYYFGHLCQLFMFCSASHELSFWSTCPS
MSVYNSKWYNKGCDKIKKSLQILMIRSQRQMTMNSFGLFELNYVSFLAVMRFSFTLYTFLKTVAD
>PbreOR5
PFLRRLHPFLKRFRFLQESAHFLADASKYSLVLAQIYLYAANMWPINFTSVRKTLFFTVIGIAILHLVSILVY
CYSYVKNVDTLAEVFQQVDLGIQVLYKSSVVFRKSNRLSNLIHIVWYEFWPSNLFGGELEEKIADDTKTVI
VPILLEIIVAGIYSAICLFLPLFAKSTLLLHEAWYPPGWDRNPMFKYAHIFKSFVAVYTDLGIICAYDLLYVI
LCTNCSAQFRLLCSAMELIGSGMEKDTVDDIQGDDNDIRNPSSHGTREEYEMELLSICVKHHQKLIQFGDE
LNMIFGSGHFLQLHTTLMGICLTSYLIASADNLIYIIFNIIYYVAHFWQLYVFCAASNQVSDSSVCVFDAAY

GSKWYTKICKDITKCLIIIMIRSKSPITMNAFGLIVVNYVLFLTVMRFSFTLYTFLRTLAA

>PbreOR6

MKSLRHRLLSFLARFEFFNSRMDMLKDKGKCVIFAGELPLKLAKLHPADRGASSIMYFLIIMVLVTIHVGL
MYACLLENSRVDAIANILGLSNGGMQTIFKATAIFYQRKEFTKILKTMRYSFWPADAVDSATEEQIWTNS
KKLLLLLSTQYVVPALYLIYIMIGPILKGSRYLPCAYCLLPNQTDESPIFEMVYAFQVITTVFLPLHIIGGND
NLYVTSCSICSAQFRLLRAAIRVVGSGHEGELIERLLRLPGVETPPSRGRSDKAKILLVICIKHHQNLLRFID
TLNRVFENGIFGQFVFSLAGIWANCYTLTIQTTIKGSVTTLAIYGSCLLQLLLFCAFSDDLSHLSADLANGA
YESMWYQNGDPDVGRCLALVILRSQKAVCMNAYGLFELNHVSFMAVIRFSFSLYTFMSQVAT

>PbreOR7

MILKNTRNPVLKLGLGLLKAEEDILEDDVKYTMAISKIVLSTVHLWPENNNVYTKLSFYMIFFVYASIELS
LLVHIFVAVKDVVALTDTMLMLSVGVQTLIKTAILYLKSEDLNKMIQSIRKEFWPSNSFGADVKIRIKSET
RKLLYLCLTSYLGGLCFLLQFILRPVVEATRVLPYNSWYPFNWSKTPIYEILYILQGYMAIIVNLNIVCGTD
FLYYFICANCTAQFRLLCKAMEEIGRENVYSHILLKTVGVTYSVISNEKSENERRLLVLCIQHHQKLLDISF
RLNQLFSYVNLCQLAASVAGICAACYLVTSDSKASASSSAPYFIGHIAQLLLYCAVSNELGHWSQQLSIEA
FKSGWYLQKCTDIRQCLSILMMRSQEGISMTALGLFELSYASFLTVVRFSFSLYTFLDKFKET

>PbreOR8

MFFLKKLRYFLNRFDLVQDNVHFLEDASKYVMVLSRLLLHFVNLWPMNFTKVRKVFFYATMGIAMGHH
FTIFSYTCRNVRSIDNLTEILPAMSLMIELLSKTFVLYGKRDQLKDIIKIVWNDFWPSNTFGKELEEKISTNT
KDILIPIILQMAVGSIYSTICIFLPFVATPSFVYHAWYPPGWDRSLFQTILNILRSYLMTYVINSMVCAYDLL
YTALCINCISQYLQLSYALKFIGTRKELEVILKIQGKDNKNPSIIQNDEGRVMKLLIICAKHHQMLIRFGNEL
NTIFGSGHFLQLVATLIGICTTSYNISQKTGHMVVMLSCGYYLAYFWQLYVYCASSNSLSVWSTYVSDAA
FDSEWHASNTRDIRKCLSIIMIRSQRPVIMNAFGLFQLNYESLLTVMRFSFSLYTFLRKIAA

>PbreOR9

MSKRINQYDSLDFERKVLYLTGVYCGRKFPNNAAFKIYRIVNTSTAFLFLFLMAAKMIVERDNLELVFDV
LHTFISQITLIIKAIYLCKVLPKYNALEERLTEPIFNQQTPDQDALISENILGYKYAAFTLHGLAWVALVLYT
ICPIIEGPILAIPFWLPSGLDNYKMFVKIYEVLCTWTLSSGDPALDLLPVCLLSIGTAQLDILNQNLTCSADR
DPDDSYEVQEKKIRVRLKNCVQHHLAIIKYLDDVQDIFSFGMFIQIFTSVVAICMTGLQFISITPDSTVFFQV
VTYFGCMLSQIYNVCWIGQKVITKSDEIRDACYMSAWETCMLSQIYNVCWIGQKVITKSDEIRDACYMS
AWETCCSSNKKTIFIFMERSKRVMMFRAGNFFTLSLATFVLIIRNAYSYFAVLVRVYK

>PbreOR10

MSFWMKLHSLLVRCQILKDCAHFLGDSSKYVIVLSKLSLIVGNLWLKSFTKTRRIVFYIMITLSIVHHISILL
YCGSYVRDVDSLSEVLPTLSLGLQVMNKISIIAGKSNQLDDLVKLILHDFWPSNVLGPKTDRRIAKATRRI
LTPIVLQSTFGVCYSICCVFLPIFKTQNLLLHQAWYPKGLDEHLIQKALQIMKSYTMNYLDCLTVTSFDLL
YVAMCVNCSAQFRLLCGAIETLGTGKDRAMIWRITNKPTGGNEKEVARELLVICLEHHQKLIKVADEMN
AIFGRGHFVQFCATLIAICSSFYNIVVQRSYTTIAFSLGYYLAHFWQFFVYCSASNYLSDWSTCVSDSAYNS
NWYSKDSNNIRKCLTIMMLRSQKRLSMNAFGLFELNYVSFLMVMRFSFSLYTFLRTVAT

>PbreOR11

ESYTPNFFRVNAEFLTYSGLWSPPYKNGIKYYLYKLYKTVVLFSVLFYNPCVVILGVIEHIHDFVIVIEALN
VGLTILLTGVKSAFWLSNGERIKEIMHRLETNVLHYEETDGFDSVRMIREAQKSGLWYALLLWRAALLTL
AFAYIPVAMLSLWYCFNDLPITGVKAFENLPYYMYVPFKRDTAFKYFLACVLQCSPCFICATVFVGIDSLF
MSMMNLIGTHMLIVQGAFRTSRKRCLKKLKGRALTEDGLYNSEELESYMMDEMKSCIEHLQMLFRSCSD
IEDAFQYMSFVQALCTMYILCSTLLLISTVNVLTNEFGKDLIYFLGVTLQLGLYCWFGNQLTLKSEGVPLA
MWESDWLETRKPYKFCMLVTMMRFKKPVYINAGKFVPLILDTQIAVIKGSYSYYSVLKGMSK

>PbreOR12

MAGGIRSWIRAYKQKELPKNYFRAQVKILRFLGVELMRDETIKYRIYSCIMLFIVAIQGTFSEAVEFYAQW
GDLNAIVNVTSYLFTHSIGIIKVMMLFYNRKKFGKMIKTLHNKPFAPEVNRGGDIEEKYLRQIVRTTERQL
MFFFVLTYVTLFTAAVTFIKSRVFDPKEEWQYPYVPITIIKTTDSPLFEMVGVYEVIWVSLYAGLLITTDILL
VTILAHLTIQFMILNNAFKTIRTRSKRMNQNADGTDENEGVILSKLLGEYIEHHLRVLELAEEMEELCNMV
YLLIFTSSVILLCFLLFQMSLYPIGSVEFIHNISYYGIIAYQIGMYCFWGNEVTLQAEEVAVAVAEADWVGA
PRSVMKAMVVIIARSQKPLYMTAGKFVNLSIDTFMRVIKGSFSYFMVLQQTQEETT

>PbreOR13

EAFKKVISIVCPGIYKINEDNFEDSAKYILIYSQIPINFVKLWPEDKGILKKILYGILVLISVIHYSSLILALFTI
ADDIDSFTRVISSIECGAQVTIKATLLYYNTKELESLIKIIRHDFWPSTAMGPKIDQDILKYTKQVSTISIIQYS
CGLTFLLLNIWLPFTKTTKQLPHYAWFPFDWTKSPMYELIYLMHSYFTGYLTCPVVVGYDILYYGICANCI
AQFRLLCQAIKCFGTDEEEEVTQRLAKLVGIDADWKKDGRLLEVCVKYHQKLINVCEDINRIYGNGHVL
QLLSSAVGICMSCYRISNENNLNNITFLFLFYLAHTSQLFIYCTISHELSYWSTCVSEAAYESLWYLKLNSE
VKKSLCMIMTRSQKIIAMHAYGIFVLNHTSFVAVMRFTFTLYTFLTTIAK

>PbreOR14

MICKKKGKMDCIDYLLILPKICIKIKQKNCILPAKVLLESVCCWPDNTSSSRTITAWILFLNLMVAEIFHAA
YVVVHASDISDAVSAGATVTTTFEALVRVHIMLSKKRVINEILSVIWKQFWPLRVILKEEARKELKMKAM
ISVLLPCTFLVSSVLSNLLITGLPFIKEHQLVLKSIFPFDWNQSYIYEALYVWQYVMDWYVLFMVNAFDFF
FVSLVTICCIQFLIMQEVLTHILSSQSKEHRKIIFGPAGENMSDHQMLWECLKQHKLLIGICDDIEQIFNKAA
LIQFAVSACANCAAFLIMKVDYGQFFKMLFYAMAHLVQLFYYCYVGQQLSYESEALADAIYKCDWHLQ
YDRSFRKSLIMMIQRSQRRVCLTAVGFVELDFGSFIRILRMTFSFYTLLNNLLMKNEQE

>PbreOR15

EILKKFGLLLDDVDFLSDDAKYSLVLSRLIVDFVCVWPTNFSGFKRIFFFAIITLEVVHQVTMFAYFFIFVR
DVEALTVVLPSLSVGMQVIGKTAILYCKTKKLEELIKTVWNDFWPSNLMGEKIENQLAQDTKRLLTPIFV
EITIGLTYSAYCIIVSLNNREPVYPAWYSFDLSKSPAYVTVAILQIFLTMYVDTCLICGYDFLYNAFCINCAG
QFRLLCHTTEYIGSEEEYRIIERIQNYANVESARTLNSVGDQEGVLLRICAEHHQKLIRFCNELNQIFGHGH
FLQLASNLMGICTSCYRISLESNYNNILISVTHYITYLCQLFCYCCASDHLSNWSTRLSDAVYHSSWYIKKC
LIKGKCLSIMMIRSQKALVMNAFGLFQLNYVSFVMVMRFSFSLYTFLKTLSSN

>PbreOR16

MEMGFFDLNVKILKLSGLWVPDKEDSNYMILVIYNSICVCYSMIYFTIAELVALKESASDLNDLIANLNMT
MSFILTLAKVFGWFYYRKNIVRIIKLLEAKENTFSENNVDNENIISEKKRFKNIWTKSFFIVASLVPISAGILS
LTDTVISGNKYINYQNDSTTIYLQKLPYHSWIPFNYASSKYSFSFAVITQCLALLNCGYITVGLDMMFVAL
VSLVTAHFTVCSMAFEKSTNVRREISKQLKDSFVYQKVLMRECDKRIKKCVKHLQVLIGVCQELETIYSP
LVLLQVLISLVVLCTCLYLVSSLPIGMRLLGNELAYLLAIEIQVATYSYVGNELTYSALEIPTAIYQSKWSS
TSLDFKKIMLITMMRMQKPVHISVGKFSALTLNTFVMIAKTSYSIFAVLKGN

>PbreOR17

MEYKRLLQESGIPVHKLPKAFTSDLSKDAAKRCIIPGKWILQTILAWPDTDNPYVVAANWFLFINMIVVEI
FHATYTVVYRADLGNALLALATVTTTLEGLVRFQMIFFKKHLFNDILHKIWKKFWPLSVLSSTKEVEILK
RRCYTTLGLTIGCYGPAVICNVVLTLGPYLTGDGLILKSVYPFEWNTTYAYEAVYTWQYFTQWYILILVN
TFDSFAIPMMMICAVQFVVWQDIFRNIFTVGSKKQRLALFGKEVNDEEMISICLEQQNMLFEICQKLEEIFS
FAILFQFGNSILALCSSCVILKVDQSKFFEMFTFAVAHMFQLFNYCYVGSELAVQSENMAVAIYDCNWQD
SDDLKFKKVLPFMLQRSQKVRNLTAAGITPLDFISYVKVLRISFSFYTLLDTLFDSE

>PbreOR18

MFFNKDNHSQNPSQEFSDDIHKDEIKKCLLPAKFMLESLCIWPGSDSIILQTIAWILFWNLVVLQIFHAGYV
LVNIRDIGKAASAWITVTTIFQALLKLHIILTRKKVLNQILTKVWKNFWPLCAVTKDNVKLRLRRYTMVPI

MLLASSLICAHLCNIIVTGQPFIIQHGLILKSSFPFDWNKMYIYEVLYVWQYSLEWYAILAIEAFDILFVVLV
AICSVQFVILQEASRSILSRDSKMQRKIIFDDRAWKMTDNEMLWECFKLHQLLIDICDAMEATLNRAILIQF
AVCTSANCCAFLIMKVDYQQFIKMLFAAVAYLFQLFYYCYVGQQLSDESEKLADAIYECDWYLEYNRDF
RKGMVLMIQRGQRKICLTAVGLFELDFSSFVKILRLSFSFYTLLDSLVMEKE
>PbreOR19
MSGKKTTLRKLSLEFTKDVYKDGVKKCILPGKILLQSVCCWPDDESLFMKVVGWFLFWNLFVIEIFHAAY
VFKNFKDIGDAVGAGATVTTTMEGLVRLHIMLTKRDVINTTLVKIWKQFWSLDVIEPIKRKQIQRKAQLS
VMLTSIFLASSIISNSQITGVPYVRNRGMVLRSVFPFDWQKSYYYEVVYVWQYYSDWFVLFMINAFDFFF
VAMVTICAVQFVIMQEVFKSILGKDSRRQRVIMFGEKGQKMTDRQMLFEALEQHKLLIGICNELEESFNR
AILIQFFVSTSAICAASLVLKVDYSQFFKMLMYAAAHLSQLFYYCFAGAELSYESGHLADAIYECNWHLS
YDREFREAILMMIQRSQRVQCLTAVGITELNFESFLKIMRLSFSFYTLLNNLLMKHMDG
>PbreOR20
MGAYNTDLFYINDFFLKCAAVWPINHKTGIKHVSYKIYQFFVITVTLVLFPTSLFVNVAQNVDNLLTFMEI
LYPAVIGLLSAAKVYFSFMNCDRIKRVMYSLECNDFHYEKIENFDPCLIMKRAKLRGIITTMTMWCLCQL
TLFLTYGSPIVKSFWYYIKDMPIGNVTTFQTLPTRLHPLFQCDTALKYLIACLLQFILFSLYILVIVGFDGLF
MNLLNIIGEHMVILQGAFRTIRNRCLLTISGIDLGVDELEERMMIEMKKCIRHLQMIFQCCADTEEIFKYICL
IQSTATLLQFCDCLMLLSLTDFRSTEFRMYASYSIAIVTELSLYCWSGNNLTSRAIDIPLGLWESDWLETRK
PFKVCMLITMIRLQKPTIFTAGNIVPLLLTTQVSILKAGYSYFTVLSGR
>PbreOR21
MSPKIVIRKLSTEFSRDVFKDGAKKVILPGKILLQSVCCWPDNESAFMSIVGWCLFWNLFIVEIFHTFYVFK
NFRDIGDAVSAGATVTTTMEGLVRLHIMLSKREVLNGILVKLWKQFWSVEVIEPIKCKKAQRKAQLSIVL
TSIVLVSSVISNTQISGAPYVRNRGLVLKSVFPFDWEETYIYELVYIWQYYSDWFVLFMINAFDFFFIALVTI
CSVQFVIMQEVFRSILAVDSRRQRVLIFGARGAKMTDKEMLFECLQQHKLLIGICNELEESFNIVILFQFFV
STSAICAASLVLNVDYSQFFKMLMYAAAHLSQWFYYCFAGDVISYESGHLSLAIYECNWHLQYDRDFRK
ALLLMIQRSQRPQYLTAAGVVQLDFASFLRIMRLSFSFYTLLNNLLMKNMDG
>PbreOR22
MEFVKGKTFQQKSILYFNLNVIKLFGYWQPKNPSKSLQVLYGFYSFLIIVSAIFNVITESIDVISLLIAGDIDT
FASNAYLYFLNFAYVIRAVHFHAFKKQIVSLIDAMDQQIFQPRTEGDMLFSIRHAKKWLLFCKAYIFCGIA
TVCFFIIFPFTDSNEIKLPFCGWYPVGDDWFVVLYLYQELQEFSLGMCNISFASIIMCILSYLSMQMDLLQN
CVNHLKERCEEKLSARSWDSSSHTFNYQQELGKLINENLVDCICLHQTILRMKHDLEKIFSITIFSIFLFDCL
ILCMTMFQFVTVPLFSTQWFSVITYFTCIVLELFGYCWLGNELIIKSNNTALTLFHSDWNGTLKNYQTNVL
IFLTLLMRPITVTAGYFTLTVDTFTAIMRTTWSYFVFLWQVYNRH
>PbreOR23
MEFQQVNFQQRSVLNFNLNVIKMFGYWQPENLPKILRCFYRIYSFVIVIFAIFHVVTESIDIIGLVTAMDIDA
FASNAPLYFLNFSYVIRAIHFHAFKKEIISLIDAMDEHIFQPRTEDDMTFSIRHAKKYVMFCKIYIFCGAATS
LLFVIFPFTDAEELKLPFCGWYPVGDDWFAVLYVYQGVQEFSVGMCNISFTCITVCFLSYLSMQMDLLDT
CVKHLKERCEENLKKKNTDLLNVTYEEKLQKAMNESLIDCISLHQTILGMKRNFEKIFSISVFSLFLFDCLV
LCMTMFQFVTVPLISMQCQSVITYFTCIVMELIGYCWFGNELIIKSNNTALSLFHSDWMGMSKYYQVNVL
LFLSLLMRPIDVSVGYLTLSVDTFTAIMRTAWSYFAFLCQVYNRDS
>PbreOR24
MHSNSQSSRLGDYFRPQKLILKICGISLRENESISYRLYSGFVMCMIVSLLFVEIHGIVVHIKDVENLAGFIAI
VFQHVLTLFKLIILKRNRAIISETLQMLHEGCFAPNFERGGAIEEKLIKECNHDIFVRSATYYIAGAITIGNA
VIIALRTKLGQDDYRLWETFWLPFELFEIKSTTCYYIVYIYCSLAITYFSTFVMVTDLLITSVIKHATTHFVIL
GNFIRNITKYKVEKNNDELYNDYPKLRNDRLRMRMRAAIVYHQEIIKVTERFENLFNIFILCEFVGSIFVLC

GTMYKSTLYSLGDQRTIRECVFLFTMTTQLFIYCYCGNELTLSSTSVSFACYEADFIDEDLWFQKNLLMIM
MRSQKPVALTAGKFTVLSLPAFVGILRVSYSSAMVLRKAN

>PbreOR25

MIREKSKERLVISEFFKPFLRILQIFSCYSPVPASSTAYKMYSVVEVTLLIFVPCFLKTVDVLQSFTELDEAL
ATLLIYVTFFIYTLKSISLITQKDDVRKLVRCLDLDIFQPRNTRQRNFIKGQILTSKGILTLFTAGGCFTCTLL
DLQPLLINRQERNGPINFWYPVVNTTKSPAYEIAYAYESIVSITHAFTHCVLDAINIICMAFIIGQLHSIEDTL
INLRSYAEKEVNIKGEPNTTEKIHVQMNILLIECVSKHRALRRFANDLNKIYTNNFFLILANCMLALAFLM
YKMSKAATLDFTFIFMIFYFGTMMTLVFMCNWYGNEIIYKSGNLHTAAYSMDWPGTPISFQKNLLFTIIHL
QVPIKLYALNFFLLCLESYISVVKTSWSYFVMLTTVSNN

>PbreOR26

MVVPMSKIGYKEALAIPISVLTKMGLWPAKKVNLVVISKLFMNIVIAIITETVMVSNIIQAARSVNMKLLN
WSICVFFPLTNLHIKALSLWINRSYFLSLLNDLDSTSFNNHPVKLNRHIQTIAKISDVIVKYFALVMVIFLSIF
SLLPSFTNLPLMMPPPFDMGKFDVAYRIGHLLATSYLATMSATIDSLYMSLIALSNAQLDILRERLIKVSED
ANELCDSMDKMKWDAGVWIILKECVLLHDTITKYISKLSAVLSLPLLFQYASGCFIICNTIIQITILNERDSR
TIIGMCGYSGIVFAQMSFYHWLGNEIIFKSDKIIEASYTSNWYELDIRSRKCILLLMERAKRTTAIKLYDLVF
VSLESLGVVYYSYFQDGFSSTFHRCFLGGSMGIFSFCLS

>PbreOR27

MFQWNINTRVYSSSTLSIKFSAEANMTIIGRRLLHVPRALLAVFAIDPFRQEKMSLRCIVLDVFQFYVTLTG
ILLMKDFQNVNADLWITCISCSQAAMKVFSMLTQRRDFFEIGKTLNYLQLHRKYSNSTDGKYEKIGIQCH
KAYLFTIVVTVINFMIKPMLIQQRILPSVAYSPCDIQYSLTCYLVNYICQCCAGIYAALMISAIDLVFFSLLF
YGYFELEYVKINLLNLKVDKKYSGDDPKVLKDIAFIVEHHDQTFRYLELINKAYAGILIYQCCSTLFIVGM
SQFCLTINGFPPSMNTLLTYLPYYLASLGQVFIYCIAGGVIADQSESVGDAAYASEWWIRNQLKMRRALSL
IIMRSQRRLNITVGGIWVLNLPTFCSIVKTSMSLLAFMKTAYDN

>PbreOR28

MQNCDKKFGDYFRVHKLILRICGVSLQTNDSRLYRTYSTFILCLFLSIMMEEVYTLTRFTNDINKVMALLS
VLLSHIVGVFKIYVVLKNRPLICEILESLHGGMFAPNRKRGGVKEEILIKKCIADTHKQIIVYCTPFGISLLN
ASIIALMTKFTEDDCEFWELPWEAFGLFKICSSLRYYLIYLYQNGTLFVVSYIIIGTDLVIIAVFAHLTTQFAI
LANFISITVKGDEEFSPTHLEIKDKSKRHYRKCMKEIVVYHQALIHLAERFEDLFTFVILAVFIGNILVLCST
MYLATLNSPRNPKSLLGLTYGITIVVQVFLYCYYGNELALTSESISSACYDDGIIGTDISFQKDLILIMRRSQ
RPVVLTAGKFANLLLPAFVSILRASYSYFMVIRTSN

>PbreOR29

MIDLNVWFLKLSGLWCILKSTKIARKYVISAAFIIIPCQGIYLPMECYKLITGYENFQRTLELLGFALTHILA
TTKIINLYVNRKEICQIIEDLNSYYIMSDHTAAEECKRLKQRFYKKTYRLGILLFVLGNCAGLIRFGISAFHL
VFCTDDTKYEELCRTMQPFLIPLPEFLDMIFLRWVLCVFQGICLNLYAWQIVACDMLFVSLLVHIDCVTHI
LCYLFETVTERSMSHRNTISDKDSGQQLNSRMNKEINMATYRLQRLITTCEKTSNVYQYVVLMQLVFALF
TLMSSLYVATSVPIFGASFIYQLEFYVTIVTQLSLYCWFANEITISFGQIPKAIYNNNWMSGDQSFKKSMIIN
TIRMNKPIYLVIGMVAPLNLNVCIYILRASYSYFAIIKNK

>PbreOR30

MNRNIMSRAFAIQEHMMSIMGYWHKENSSAFYKIRGTVTMLIFLVAYVLMLMQFFHDIVDFRKFSEILC
MLMSYTSYFCKLCGYLYSRKTFLEMLSHLKNPIFTRCSPEMERHLIKTLNLSLFITKLYRFSCGMVVILFSI
YPLFDDKPLPTSVPFETGRYTFLLYIIESLAMLIAAWDNFCLDTLCTSLMGTAIGQFDILKEKILTFKERAIG
EEEIVLGVNFKTTNIFEANVNKRAKSLLNDCIVHHNALIKFVQQIEDTFSFGLLSQLMGSIIVICNTGFFLML
VSPASLQFGLFSSYFITMMAQLTLYCWYGNEIMLKSVQIGEACYFSQWYTCTADVKRSIFIIMEKSRRPLAI
TALKFTTLSLETFAAIIRSSYSYFALLQRLANKFESETK

>PbreOR31
GTMADPNQRIHVSGFLKLYLWILRAICYYYQEPGEPLWYIVYSAVTMFLYVVAPAILNLIKIIKCLIAERDP
EILARGFTNVTYTSEVYCFLVQKNNITQVFNDMDSDIFQPRNEFQRAIIRRQMLKAKRFIDISMWVTCALC
FLYGIKPRIRNDTSLPMNVWYPVDTSKSPAYWVVYVYEYIFMMLHACSHVSLASIPILSMAFLIGQLNMIA
DTLRKLKEYAEEDAKTDNKKDNTVKTHVEMKKLLIQCVYKHRALRRLVIALNKVYTYHFLMILSNAIIG
FALLLYQLSTEGLQSIITATFHIATLMTQLLLYNWYGNEVTFKSDTLYIAAYSMDWPGTPVWFQKDLLFA
LIHFQIPMKIYACNFFLLCLESYVGVLRTSWSYFLLLTNMNEE
>PbreOR32
KTHHISKNLIASMTTATRPNPFDAFRTEKGILTWFGFPVGNDWKGFKILKGVASIIISVSLMVSMIGRIALEL
DNIPLILEALYFGLTQTTFLCKIFNILVCNYKMRRLVDYLKNPLFNQHTKEQDIFIERAIRICHIFANTYRVC
VGLTITFYAIFPFIEHTLPLPGWFPMDSKKYHYVLYIYHLACLIINGYPHTSLDCINASMISMASAQFEVLK
DNLNHLIKPNDMKLTIKKQDEIIKKRISKCIIHHNMIIKFVSHIENMYSNVIFIQFLCSIIIICVTGFQVFVMSPS
RIQYITLVLYFLCNLTQIAIYSWFGHDILAKSGGIGQACYMSEWNNVSPNTRKLLFIIMERSKIPIILTAGKFF
NLSLNTLMMILRTSYSYLAVLQQMYKT
>PbreOR33
DDVIKGIISPTAVLNYRKEMLEVLYRCKRFCGIWPDENKPIYCLKLLINITSIMYLLTGFSLHVLKVVTDPA
GTVSNIADALFTLQCTFNYSFNYIGCAILSETSLNFFTSITNFEEFGMPKQLPAIDRELNKNVKIMVGYCTAS
AISIHLYSLLTYDYCLEKHNGINDIAYCGYCCGLYYPYNYDKGFSFVLHVLLNTYTFSMLCFSGVIFICAG
YCGIEFICVRIDHLIQMLDEVLNGRKDSKETRDGMRNCIRYHIYIISLANSLNDCYSIINTPAVFFYSVTLGL
CLFSLIKDYSYKAALVVVGYICGTFVMSVSGQKLLDKSQSIGQAVYDTKWYQHDVRTRKCVLLMMLRS
QKALTYKAGPFGTFSLPVFMLVIRGAYTYMTMQSNIK
>PbreOR34
MPENSYINTAPNSSKDRRIRFDKSKMAFVKWDAFELGFIVLKTVRLTNNNLATKILIFHLVNTVIYAEVFL
LTMVNLFRVEKEDFLDAMEGAISTLHAWMKYIWIVYYQEDLLRFQEDACEFWDYRVFSEKAKMYAIFL
KTALEKLQLTLGMAIVCLITLYMSFPLFSAGRFYVLNLNVHIQSHLLRYSILLSQCYAVMVSACAVYLYEII
FVMLCCQAAIQLQLLSEKIKFEEGNDPKAVLREIVKHHQLISSVLARMKKQYSLVFLLQFVIMVASACSEL
LFISIGNTDNWVRLMKSFIYCTYLFVEYAIYCVPSEEICSKVSQFSQAVYFSKWYDMLKIDAKDVAFLMM
NSQKDVYFTIGDLMDVNMEAYVTVVRATFTFYAFVNTMLLE
>PbreOR35
DGQPIKTYLFYLSIQKTAMRKLSFEFSRDIYKDGVARCILPGKIMLEGIVCWPNKDTLFMRMLGWFLFYN
LLLVEIFHAAYVFKNFTNIGEAVSAGTTVTSTMEGLVRIYTMLTKREVINRILVKVWKQFWPIDVMDPTK
QKEAKRQSYISIALTSLVLLVSGTCTVFITSVPYLTNRGLVVKSVFPFDWSKSYVYELIYIWQHFNEWLVIV
AINGFDFFFIALVTICYIQFIVMQQIFRSILTEDSRRQRVIIFGKRGETMTDREMLFECLEHHNLLTGICSELE
ESFNIAILIQFFVSTTAICAACLVLKLDYSQFTKMMSYVVGHLSQLFYYCLVGQELSYQSGHLGVAIYECN
WHLSYDRNFRKALVLMIQRSQRVQCLTAVGITELN
>PbreOR36
CRLLCKIEAMARISCTRTIEKPKRILSMSGVWPTAKITKYTVLNLIFNLSFDIIVLTVIILNILNALKFKKINLL
NKMICIVISMVNYIAKSSTLTINKECFMSIIQDLESTTFNGHAMELNYHIESIYNNCNLVFKYFAFMLVIYSL
IGSILPMIVDIGITIPAPFYTGRYEFLYKVLHFFATGLLSCNTIGLDILCLTLISLGIAQMKILQESLENIVEDRE
ETNGNKYFLNLKVDSTLRECAILHQKIIVFVGKLNTLLSFPLFIQYTCGCFILCNTVVQLTLLREPGTSNTVS
MIGYGAITFGQMAMYHWFGNEMIFTSEKIIESAYLSKWYEIDVSSQKTILLLMERAKITQSIQVYKYIFISL
DSLGVIVRWAYSVFALVKARYG
>PbreOR37
MDQQEIRCIDVLAPLEKMLRISGIWPLKHMTFKYKLRTAISWLFSYVFLASMIAEVIHVKGDVEKINEVLC

VLTPICGCGSKQVSLIYYKKNFFNILKILKSDTFNKGNANMIQKIMTLTKRGKKIFHGLTFGTLVLYSVIPLF
DQKDYPIPFSFNVGRYKCAVYLFQVASLAIVAWNLTTIDLLFVDLLALGTGLANNLGKKLSNIVGGKKEC
NDYCKISAKLNVEEIMNKVSLKSLRECIKLHISILRYINDIEAVFSFSVIVHYMTATLLLCSSLLQITTMVDIH
STQFIRCSWLAVNALLQSFLYHWFGNEIIVRTDKISEACYSSSWYKCNVELRKMLLIVMTASRKPVGVTIY
KFTELSLVSFVTMLRWAYSFTALVRARYVPD

>PbreOR38

MFQKILTEEVFFLLNIRGQSLDASTLSSKLKAIVCLSLEGLLGILLTVGILDSTDDLYSVLENITGLITLLQIIF
RSVILFVYKDNFLNSIIFLKIFWKADRFGATSFKKISNFRLQFVKILRIYRLIIGISGTLYLTKPLFEKHRMLP
MTCLLLCDIHNDVCYVFYYIFQIIALATQLIMLVGFDSLFFVLLMCAYVELEQIKQALSNLSMKENAKGD
QDEVLKEMITIIEHHNLVLGYIGIFNHLFKTALLFQFGFSIFSLCSSLFVMTTNGFPPTTGNFLKSGPYCFSGI
CQIFIYSAVGELIAQQTERISDAAYETKWILNYQPSFRKMLLLLIQRAQLRSQITVGGVWKLDMTTFASILK
GSMSLMAFIQTVYYNKK

>PbreOR39

MADKKDISYKRLLSKTLSYLGIVGLNPLAHTKASDIKAVVILTLEVLLTFSIAASFSINLSEPYAIIENFTGFA
PAVQIVYRKIMLLMHRSDFRDIINYLDYFWSINAFGKHTANQVIRIQKMQSKFLKFYLWTVILSGIAYVAK
PAFTDERELPVLSINICPIKTSSACYLGSYAFQSSCIAVLLCHIVLLDSLFLNLLLYGYCELEQIKYAFHEVK
AEVHVHDEEPKVLQLTILIKHHIKVLEYFDKLNKLFSLMLLFQFSSSLCSLCTGLFMLTSNRSPPSSSFALKF
VSYVISALIQIFAYCLAGQMILEQNESITASAYFNCKWWEAYQPKLRKGICLIIQRSQRPVNIVAGGLWKL
EMDMFVKIVKGSFSLLTFM

>PbreOR40

AIYGLNGLTNMHMIGRLLKSPYFLLKTVGLNPFDPKRFGFRSIILMVAQIVMAVLILMGLKDFRSVTADV
WVSCIMALQGVIKGSSAILQKDAFYNIGEALNCFLSRKFTSSEQKIEEFVHRMQYTYTITLILALVAFIIKPA
VVKQHILPSAWYDVCDIEATITCYLLCYGYQCYASVYCVIIITAFDCMFSGLLAYGYIEMEYIKRSLLKLN
DNENCTGDNQEVLLNIGILVERHNQVLRYFRDIDKAYSGILMCQYAATLMIICSAIFCLTTSGNQPPVDVIV
TSVSYFLAGITEIFFLCLSGDIISLQSESIVHAAYGCNWNHKPKLTKALCLMIIRSQRACRITVGGLWILNLE
TFTSVMKATMTLLTFMKKMYGR

>PbreOR41

MEDEYVMKCGLYYLHLIGTNPFKRSIRGNIICFIQTFTAIYSLGMAIDGVISKYENIKSLAGSIDTVPSAIQT
VIKLLSAIFLRQKMKNLYLMVEEKWPDDIFGREFQNKLNKWSKMFKTSYKVYLYFYVFTLVFFISRPLLR
HSRILVTEWNLPCNVASNQCYGFYVVLQASYFSIIVSVVFGFDIIFYAFLFYGFCELEKIKYAFEHLSISENV
EGDEEKVYVEFCKILKHHDYILRFLDMVSKVYSLQLLSNFSAYVSTIVFGVFFMNVDGFPPSKENLAKYIP
YLITHHFQLIMYCVLGEMIRTQLSSVSDTIYLSKWYVKKQPKLTKAMIIVMQMSRIPNKILIGGIWAMDLD
LFMKVVKTSISVHAFMQTIYQVD

>PbreOR42

MTLNGRRLLDLPCAILAIFGIGLFSPAKISLWCLFLLLIQFLVFITALVAFKDLRNMDADIWVTGAAVSQGI
VKVIVLLIQKPKFSKIADALNNFQLRRGRSMTINAKREQKFVQFGMRCQRIYTFIVTWALFAFITKPVVKR
ERITPSPAHYFCNIETLYCYVLQYTAQCWAATYTIYILVSVDCLFWSLLSYAYLEMEHIKDILLHLQVNKQ
RVGDDWEVLEQISFVVEHHAQILSFVQLINDAYSGLMIYQCACTFFIVGMSQFCLTINGFPPKLTVMVTYV
PYYVAGLCQMFTYCIAGDIITNQAKSIGDAAFNSHWWIKHQPKLTKALCLVIQRSQRTLTVTVGGLWVL
NLETYSSIIKTTMTMLAFVKTIYEN

>PbreOR43

KKDLRYSMFCWPDNDALYVKIIGWFIFMNICVVTVCHGACLFKATTEISVIISVAATFNALIETLVRFHILL
TKKKILNEILVKVWKKFWPAQAILRPEIRNKLRKRVIITFVLSIIFLITGTISAAEITTVPYLKERELELRLNVP
FDWTRKFLYEIIYVYQYFIVWYSFSMINTFDIFFVALVNVCIAQFVILQEALKFILSEESKRQRRIIFGGKAE

NMTDSEMLSECLKQHVLLISICNDMEKSLNRAILMQFIVSATASCAGFLIMKIDYNQFSKMLFYAVAHLIQ
LFHYCYVGEHLSNESERISDVAYECNWHLEYRMEFRKALILIMQRGQRKICLTAVGLVELSFASFVQILRT
TFSFYTLLNTLLVKRN
>PbreOR44
MGTIQQIPMTEVAIFLLELIGEVGQRSSASIMIFRVFNCSVFVIIIWYAFANLFVVTGESFVNSLLSVLYASH
GILKYFLLIHYKPIIESMLKDIGSKFWDHRDFSINIAETAEEIFRKVNFVQKAIVGGVLIVMYSYHLAPLFDS
NRLFLFETKAPESPFMNAVLLMSQYYCVSMEVPIVLGYDCIYFTICIHLVLQLRLLKQKITNLLETKKENV
ESEIYKCIRYHQFLISMFTRMKDTYSAMLLFHYFDTMITTCSVLYEVVDGGYTTILNFIGKLITMIFFYAQF
ACYTFPADQVAEEFSGLSHSIYNSLWYQNTIAVQKTLLFMMMRAQRTHYFSGLGLIDVNVDAFGSVVRK
SFSFCTMLRNFVNKEVEV
>PbreOR45
HSIQQKMLEPQHTIEQLSRLISKSMLTFKESATLLFECIFPLLVTLCWIFEDLSSYVLMESITGGIPAYQGVIK
KLVLHSYKNKIREIMESLKKFWPKNKFGKAQLVKVNACEAEIIKMLKIYSMVLWIGYVAFVSMPLVQTK
NLPVTWVTFCDIENNIYCYIFNYVLQSLCIAKLMHLLLSLDTLLFVLLWHGYCELEKIKYGLAHLGENDFT
ETVAVQDMASLVKQHSLVLIYIQKIFDLFSNLLLFQLLTSSTGLCACLFVLKAEDEASIVAALTYVPYLCG
CLLQAFLYCGIGQTIYRQTSSVADAAYASNWVQKNAPTQRKALCFIIQRAQKPEQMTAGGVFALNMETFI
SMVKGALSVVAFLQAVYHSK
>PbreOR46
MAIIGRPVLKLPLFIMSLYGLNLFNPSKNYRKFLIIWLAQTVIASTTLVVLKDFQELDADVWITFMAASQG
TIKSVVLLIKKETFVEVKELLNNLRLQRGNVEDTDSMRNFECFAIRCQNTYSFLQLLSLLAFMGKPFVMD
GRILPSEGYTPCDIRGDACYIFSYILQCYAGIYSILVIVSADDLFWGLLSCGYIDMEYVKHILLHLKVEERVE
GDDSKVLQQIAFVVEQHNQILMYLKKINRAYAGLLVYQCFITFFIVGMSQFCLTASGFPPPLHIVSTYAPYF
LASLFQIFLYCMAGDIVARQSETIGEAAYGSKWWLKHQPKLRRALCLIIMRSQRTLKFTMVVWVLNLETF
CAILKTTMSFLAVMKTFYE
>PbreOR47
MLKDDIKRCFIPIKIYLEALCCWPYNRSAFIKFCAWFIFSNMLIAEIFHAAYVVTNIDDVGGAVGASATVIT
SFEALVRMYILLTKQDFIKEILLKVSKQFWPFQAISNVDTRNQLRSGVILSISLPFIYLTCATISTGGYVFMSL
RSYQLVYISVFPFDWSKRFVYEIIYFWQYIINWYNIFMINVFDCFFVSIVAICTAQFVILQEALRVIFDEKSR
RQRRIIFGMKGGYMTNREVLLKCLEQHKMLLSICTDLETALQRATLIQLAVSVNASCSACLMMTIDYSQA
AKMLSFGIAHLIQLFYYCDVCQKLSTESEQLGDAIYESSWHLEYDRDIRRTLILMMQRSQRKVQMTAVGV
MALNLSTFVRCFSC
>PbreOR48
MSWLSELDIFIFGLTLLLFIGEYMEYTPKEIKVFRVINCVVIITVLVFILGNLADAKSGEFVSTMEDFTTVFH
TLFKYLIFMYNKNNLKELMDARTERFWKIPHGNEKLARKIRNIYKGVNIVQVVMLADVVPVIEIYFLTPY
FNPSNIFIFPSNVFVNSVVMDAFVLFCQYYFASFVAFIVVGYDFIYLSLCTELRVQVKLLKYKLREVFTKTS
EDPVSGISICVEYHHFLLSIYERMQKMYSSTLLFHYFVSLVTVCFDMYQSFVGESHLSHELLKFLMLAAIIA
QLAFYCVPAELLSSEFTDIAQALYMSKWYKHKPDVQKLMLPIMVKCQRPHFFSAAGLMEINLDAFGSVIR
RAFSFCAVIRNVLDK
>PbreOR49
ESARVGKMLPANYMSQVPLSILRFIGEHMYYSSKLLNLYRLFNFVVILTTAFFVICAPFNKEKLTRYAEYL
ESSAVTVQVGIKYLFFVYNKSRIRNLLAELAKIPWDSKDFTRNFLTSDQKFQKLIRLAALSFTIHSTIVVLVF
LLRPAYQKYAYILETLDVDSIILSTMVLALQYYILMLVPAVVMTCDCLYLALCAEIMIHLRRLRHRMERLF
ENIDDTSGVRNEFCELIKWHEHLLRVFSRLQEIYSFMLLFHYYTTLQTNCAELYTFLGSAKFEIKYLISVIAI
VSEFALYTFPAEAITVEFEKVSTSLYMSDWYKSSLDCQKLLLFTIMRAQVRECLLGGGIVEVNLRAFTSVL

KLSFSLYTLLRNI

>PbreOR50

MKQIPRDDIMQMGLALLKIMGERMHFKSTLLNCFRTFNITMMVVNLFFVLAYYPQIGADYAKYIKSTEC
ALTIIHVIIKYSLFIYHKRNIEDLLDHLLEFWDYNSYGDNIRLTTSTLFRNVKVGQFYYSIVTLACIALVFLK
PYVNPDNRFLFVCWTFSGSTELETTVLACQYYFWSILYPIVLGYDSVYFSYSMHIIVQVRLLKQRLQNIPS
NVHIEEIVTCINHHKLLISIFARMGIIYFWMLLLHYFITLVTGCSLLYVILLGAADNADLFATIFYLIGLFIQF
AYYSFPVEEIVFELTDISRAIYMSNWYEQNVKIKKILLFMMMKSQRQNYLSAGGIIDINVDAFGSVCRKAF
SFYALLKTVIDR

>PbreOR51

LIMSRLPEGDILEFALQVYATFGENLSNTPKKIYVFRAINVVMLSLTVIFILEACIEEEGIVLVKTLESAITVL
HVLIKYLSFMYSKEGLKAIVEARLKFWNPQAINEHVAQFAKKLYSYIKTLNTILLVVFITSSEFYWLKPYF
NSGEKFMLNARVPYNFLPLEAVALLMQYHSLGLITPIVMTHDALFLAICGHLSVQLRLLKCKIYEAAVGE
WEDLKTCIEYHQFLSRIFIQMQEIYSWFLLTQYFISLGILCVQLYILNSRALNIADTIELLLYLGTMYCEVAF
YSIPIEDISFKFLDIGNAIYMSPWYEKGLVRRKKLVIIMMYSQAVRYLKGGGIIPVNVDMLGSIFRKSFSLY
LLLKNVIQK

>PbreOR52

SNEIDLFKISRILLQLMGETYNKNTLKRIIFFRIFNSVIMVYTSLFITANFFYASGGNHVETMQGLILVTHTLL
KYLLYIYFKPGIRELLQNKHEESWAYQDIDNHIMAAIIKMSKTTELIQVFVLLAMSITAILYLFRPILTRNNP
FPFEAWVSGVIIMDVITLACLYYFFCIVIPIVLSYDSIYLCLCIQTVGQVKLLKNRLRYFQGSVKADISYYIK
YHQFLLSTFSGLRRLYSSMLLFHYFVTLITACTGIFEVLMGRNSATDIAAKAITICAAFFQFAYYTFPAEMI
AFELSDLSNSIYLSKWYINTVNVQKQLLFMMTKSQHQLYFTGGGLIDINVKTFGSAVRTSFSFYTLLRNIL
TK

>PbreOR53

MSEKIAHQFMGVNVKILKFTELWLEDGGLVISKVKIIKLFVIFLMVCPTCVALLSECVIVFIDSEPDIVHRIQ
IILAESCVLGAMYMVICFWKNRSQIRDLVDSIGDFVNYSELDIVPVDKKVGFFTAIVVGYSLLGMIIYTLSP
LLLIESCEKTKSQYRVDHGIPCGVIVPIRIHVHPENPHMFRLYLAGQVGNGIMAVLVVVNITMMVCGLVE
HALFQLKQVRSLMSKLSESANIEERLGFVVKYHCVVIKFINNINKYFGSQLVLHFTLTSVVISLLGFQILMV
NDRKESLMYALHLIGWFIMLYNICYYGQLLIDESIGVASDAYSVPWYNCSIRVQKDIKFIMMRSQKPLSLK
AMNLGI

>PbreOR54

MGGGIRSWIRAYNQRGLPENYFRVEVYILRSLGIELMREETIKQKIYTFFALFLIMFQGTFTEALELYVQLG
NWNAALNVTTFLFTHFIGMMKVAMLFYNRKKFGNMIRTFHSKPFLPDTNRGGENEEKYLRKTVKTTESL
YIFFFFLIYVTLISGAVAFVQSRIFNDKEDWRHPYVPITVVPTADTPIFEIAGVFQVIWVSVYATLLISIDTLL
VAILAHLTIQFRMLNNAFRSIRTRSKKIYQTTEGDDKDEGVILNKLLGGYIEHHLRVLELAEQLEELCNMV
YLLIFLSSVVLLSFLLYRMSLHPIGSIEFIHCASYYCIVVYQIGMYCYWGNEVTSQSAEVAKAVAEADWV

>PbreOR55

QMMEQEYVLASGLRYLDLIGLNPYKSTWQQRIRALMFVSSAIFGYFLNLIGLFYNCLDLKSIADSMDSIPA
AQQVTVKLLGAVIMKKQLYNLYKTVEMKWPNDVYGPELEKRLNELSLSFKRIYMVYRGTIWFAVLLYF
SRPLMLFSKVSITEIYLPCDLSSNYCYASGLFVQIVYVVVLASVIYSLDAVFYAFLFHAYVELEKIKHGFIN
LPITEDMLNDKVTYQEFSKLVEHHNSVIKFLDDVNNVYHLQLLNHFITMSAIIVFGVFSINMDGFPPNLSK
LIRCVPFLISYHFQLYIYCVSGEEVYNQLCSVSDVIYNSKWYIKRQSNFTKVNLCLTYFEHATLASLGAFL

>PbreOR56

MGRFRCNCKYIVLFLYTYFGVLVGLIKVTLLFYNRKKFGNMIKILHNKPFVPDINRGGEVEKIYLRKIVKT
TETQMIAYSTLLVTALWSGAVSFLNSRIFNEKSEWRYPFVPIMIIDTTNSPYFELAGIYQTFWISFYGLLIVT

ADIVLTIILAHLSTQFKILNNAFKSIRMRSRKMNELAGGDSRNEGIILSKILGEYIEHHLRVFELAAQMEELC
HLMILAELSGSVLTLCFILYQVSSIPPNSFSFLLYFFYYWIVVFQISLYCYWGNEVTLQAANVAKAVAEAD
WLEAPKSVRKAIILVTARSQKPLYMTAGKFVNLSIDTLVRIIKGSFSYFMVLRQRGISEG
>PbreOR57
MENHLGDFQQKSVLEFNLRILQIFGLWGIENKLNRAHKVYRFVMVIFFSLHVFSQVTGVVQLARDNLLDA
LELIYIPALSTGLFIKVWYMVKYEKGLEDLINSMDEGIFKPRTTEQAEFVEKYTHSMGVLFRTFIIASIFTWT
LFYATTLKNIQQRSLPIAGAYPYKQELFFVYYLFQGLEELLVAFCNVSVHCIITGFLCHMRMQLHLLNENL
KNIEALSVQKLDKRQGISPKNSVVREKLQDMMDETLVECVEHYGSIIKMKTDFEDTFGATIFFVMIVDCF
VICMTLWQFIVLSFRSFKFFDVLVYLTCVITELLLFCWPGNELILKSSEVPYSAYESEWIHRPLY
>PbreOR58
MVETKSSEIFRASSFFEPFLHLMRAFCWYSSELASSICYNVYSAIIAIFFIMLPSCLGTITVIETFSQLDEILDV
LMLYTTTLSYAIKGYYFIKQKEDIRKLLGDVDLKIFHPKNAHQKYIISSTMLKIKRYVKIMIVAAAINTNIW
SICSILNNDLPMSLWYPVNKYRYPTYQIICVYESLYLGAHGVIHPTLDAIPLVLMAFIIGQLDIITDILSNLKK
YSEVEMNVDESDNSTKDVLHQKMNNMLIECVHKHRALIRLVQILNRVYTYHFFSILANCIITLAVLLYNIA
MEGSFNLSFMYRLIFFFVVLFELLQFNWYANEIIYKVKIYTCYYHGFHLDE
>PbreOR59
TKVKGIIACRSPAAIYIYRHYRPTFWYIGTTMGPDLYYGDELAIKGVIKEFKNSARAKNIAKRSLLNSADG
NVLDQHDQFYRDHKLLLKLFQVLGVMPIQRGEIGKITFSWLSVPTIYAYCFYAVTTVLVVLVGYERLIILT
QKSKKFDEYIYSVIFVVFLIPHFWIPYVGWGVAREVCNYKNSWTRFQLGYYKITGKSLVFPHLNALIVIIST
GCLILAVAFLLTLSALLEGFTLYHTTAYLHIITMINMNCALWYINCRAIGNASKSVADSFEVDVKQYCGSY
IVKHYRILWLNLSEILQKLGNAYARTYSTYSLFMITNITIAVKYHYGNIMVLIYT
>PbreOR60
MGLVRSHLLDLPSLLLSKIGIKLFTLEKVSLKCIVLLLIQLIVVIMSMVILKDFRTVNPDIWITTVAGSQSSIK
MFVILTQKQKFFEIGEIVNNLDLRRNCEQAFTKEQKDFKRFFANLQLIYIFIIITGLTALAVKPIVLKERSLPS
PWYPVCDIQTSITCYVTCYMILCITGIILVIILLSVDLLFWSMLAYAYLEMECIKYKLLHLRIDKNASGDDA
NSLSQIASCVKHHNQVLTLIEKINHSYSGMLIYQCLVTLFVVGMSQFCLTATGSTPSLPIMLAYIPPYLAGFI
QMFGYCFFGNIIIEQWSIYSSQKVSVIQLIMQIGGPKVNLSSQKLLC
>PbreOR61
MLTAIIFILSNLAKAEGEVYVETLENFISVFHVLFKYLLTMYYKKDFAELMQKKAKHFWRNSRNGDKHLT
ASIDNLYKGINILQITMAVGLIVIAEIYFLIPYFNPNTIYFFHSYVFVDSVVVEVFLLACQYYTILLVPPIIMG
YDYMYLCLCTELAVQIKLLKQKLKETFTSTNGDILKSIAICVEQHELLLWIHNRMQRIYSITLLFHYFVTLI
TICFDMYQAFVRQNDLSNEILKMVSLTAIVAQFAFYCVPAELVSSEFADIANAVYMSKWYNHKAKIQKLI
LPTMIRSQRSHYLSGLGFVDINIEAFGSVIRKAFSFYAVIKQVLNE
>PbreOR62
MDTNDIHLKINMKSLFVLGAFRFRFKCWLTDIAYRTYSYFIRTYFVTFIICEYIELITMPDKRLLSIVEILAVS
LIYSTAAWRLKVYNSKSFNKLIRQLREVEHDIFSVNNTDLLKIYNEHVRTNSRICTGFMWIGVLTVIPYYIH
PILQEASANEATYMNVTHNNITKLLKIRPLPLSSWFPYNRYEYYYYSYAYHIVAAAIGASMVVLTDLLFVS
IMIFLIGQLKTLQYHFKNAKKIAMVLKLNIGTTYNNSLNYTIKYGIRMHQFIIRYVEDLDKSMSRLMLVDF
AVASLQMATLGLQMIVVKRYIFKQFFRLSNILRRPLLSLT
>PbreOR63
MNNFFDVNFTILKVAGIWVPSPNSSYRAKFLYFVYNSFWITYSCLIFCPSELAYFINTYTSLKDLIKNVNMG
MTHFLANIKVCMWFYYRKDIIGIIETLDIYGKRYESYGDFDNEKIVQKEKKFKDKFSVIFLLFGMLTSISSC
MACFYQTLKLKLQEDERVQLSLPYFSYIPFDYEYSKVKFLIAIWYQFFPLFNYAYLIIGFDTLYIAILSYISA
QLDIIYGAFETIRPRCMIRLKLDLPKNILKDPPMLMKEMHKEINKITYHLQVILDICRRLEDIYSTIILAQVLI

SLIVLCTCIFLVSLMNQNKYCSYR
>PbreOR64
MQHQTKDEILHAGLTLMSFLGESSSVTSKKLLAFRVFNSCLPVYLLVFVFANFPHAEFGMYLKYVQSAIT
LFHMLFKYVLFIYFKQDIEGILKDKIHFWNYRKFDEKTIRQTTNIYGIVNFIQNSSIAVSFAAILLYFLKPAF
NSDDVYIIDAWIFTDNVIIEVTVLACQYYFLIVIAIIVPGYDSIYLSLCTHVVLQLRFLKRKLKQLSKYSHGS
AEREIRMCIQYHQLLISIFKRMKRIYAIMLLFHYFVTLLTGCLDLYELLIRKSNLAYMAVMGMSVVFIYAQ
FGCYTIPAEAVATEFSDLSNSIYMTNWYD
>PbreOR65
MLNTFLLYRQQQKDTEDDKKIEQLGKRLQKAYAYALAVVLICFMTKPILVRQRILPSVGYVPCDIRATLA
CYLICYASHCFGGIYTVTCFISTDSLFWTFLCYGYLEIKYVKHNLLNLKINKGRGGDDPEVLEKISSLVQHH
AQILTYLQKVNDTFAAMLVYQFVASLCTLGMALFCLTMEGFPPSLRITVMYSPYYLASAQQIFVYCLAGG
IISNQSASVADAAYNSEWWSKHQPNTRKALSLIILRSQREVKITVGGLWILNLNTFCAIVKAAMTMLTFM
KNLYGRE
>PbreOR67
LIMALPLLRYSNNEAPKNYFALQKGVLKILGISFSGDESRWYRIYSFVWLTSVIISFSVIELYELYIYKDDM
DVTINNLSYLGTDLLGIAKMSVLLYHRLNIGKILDRMEQGVFSPNKLRGGDFEEELIKRCILFSNRQTCLY
YLSVGMVVFIGVVASLLKRHLEPEAENWEMPYTTFSWFDIHRSPNFEIVCFYQFCWRGLYALIVSSIDSLI
AGILAHISVQCKILQNGIKKSIPYSKGCRSRKKDSFFWIQSVFQDRS
>PbreOR68
MTQKPADVIRFGRKALWLFGLYHGKNNEKYFFYYTIRTITIVVISMFPLLLLLKLILRPCDVHIFLDSLMYL
TTITWFCIKIYLHLYRLKKLRKLEDFVDSKILNLQTEEQARFVAGAMTKQKLVISTFRYMTYIFTAIFALYP
IIMGKQDLIMPIWTPFEPQMEELATYVFETFYLSYVIMFYPSLDAIYIGATQTLVSQFQLLKDNLKRALDRS
AWDSTIKENIETKRQLKICVAHHNAILE
>PbreOR69
GIAAYYLYIFRPILTNSNPFPFDAWVSGVMILDILELAGQCYYLCLTVPVVVAFDAIYLCLCARTAGQVKL
LKNRITYFQELVVLDMGHYISCHQFLLSTFAHLRRLYSSMLLFQYIMTLITACTSIYELLMNTTNTNETVN
NTIVIWVILLQFAYYTFPAEMVAFELSDLSNAIYLSKWYLNNIDVRKRLLFMMVRSQHQSYLTGGGLIDIN
VRTFGSVIRTSFSFYTVLKNILAK
>PbreOR70
MDVLVLVCQYYCVALIAFIVLGYDFLYLSLCIELVVQVKLLKSKLKEVFTETSDDTAFNVGKCVSQHNFL
LLMHDKMQKMNSNVLLFHYFISLVTLCFDIYQSSVEDAELSYEIARFVSVAGIIGQFAFYCIPAELLSSEFM
DIARALYSSKWYENKPNVQKLMIPIMVKCQRPHYFSASGLLDINMDAFGSVIRKAFSFCAVIRNVLDK
>PbreOR71
MNILQKYRISNEIVRKAVWSRNKLMRVIQITMITLSAFLWMIYISKALITIDYVFVYETAFPDSYALCALVL
LSQYYFSSIQVMAVLVTNFMYLLLCAYLILLMQALRGNITRIINSKRDFLKEEIFDCISDHQTLLSAYSLMN
NVFSIILMFEYYSLVIFNCSVILEFVLNPNKTVLLQESLTLVFILFHFGFFTFFAEQMA
>PbreOR72
AKKLYSSTKTLNTTLLVVFTISQEFYWLKPIYNPGEKFMLNARVPYNFLPLEALALFMQYYSIGIVTPTVM
THDALFLAICAHLSVQLRLLRCKIYEAAAGEWEDLKKCIEYHQFLSRIFIQMQEIYSVFLLTQYFISLGILCV
QLYILNSRALNIADTIELLLYLATTYCEVAFYRIPIED
>AcorOrco
MMQFKPQGLVADLIPNIKLMQFSGHFMLNYYAETTGAVHTLRLGFCFGHLFLLLLQFGFTFGNLVQQSD
DVNDLAANTITVLFFTHCITKFVYFAVRQKLFYRTLGIWNQSNSHPLFLESNNRYHQLALTKMRRLLIVIM
IGTIGSWIAWTTITFFGDSVHTRKDPNNENETITEEVPRLLVRSWYPWDAMSGAAYYVSLVYQIYYVGFS

MLHSNLLDSLFCSWLIFACEQLQHLKEIMKPLMELSATLDTYVPKSADLFRAPSASSQDNLVDSDYNQSN
EDANLRNLYTTHQEMGVTYRSGNLQEFSSGGIGPNGLSKKQELMVRSAIKYWVERHKHVVRLVTAIGDA
YGIALLLHMLTSTIMLTLLAYQATKIDGVNKYALTVIGYLLYALAQVFHFCIFGNRLIEESSSVMEAAYSC
HWYDGSEEAKTFVQIVCQQCQKAMSISGAKFFTISLDLFASVLGATVTYFMVLVQLK
>AcorOR1
MSWGYKIGSFLNSKILKNEDEVFQDDAKYITMFSQVFAKIINLWPGDDGVSKKFTFGLMLTSVITQELSLV
MYLLTTKINVDVVITSMASMIILLQSIVKSCVYFYNARKLKKLIQTVRKEFWPANIMGETTHNDIKYNSKI
LSLVFVVQYASAILFLWFSVLLPLAKPGRKLPHTSWFPFDSTVSPLYEIIYVWEVYLTAYINANIVCSYDTL
FCSICGNCISQFRLLSAAVKCIGISGKENQISNRLLRLQGVDYNPVRTNQGKEDRESRRLLVICVNHHQKLI
KITQELNEVFGPGHLAQFFASALGTCTACYKIPIEKNPGDLFVLIAFYIAHVSQLLEYCALSHELSYWGIKL
GDAIFESFWYIKKHSQIRKCLPIIILRCQRAISMNALGIFELNYPSFLIIMRFTFSLYTFFNNMSKSTI
>AcorOR2
MVSKSYFKVQIFCFKMMGILMEDIDWNRFTSRAYAIYSFLLLSCFIYLFIITEAIDLILKWGDLDNMTFNLC
YLVTHFAGLCKIAVIMHQKSKIRTFYQSLESGYFLPNHERGGNEEFRIISSAIWQSNMQTYVFYTFVTVIVA
NRGFYAGFDKGYFIQFTSVNGSETTNKHYKVMPYTTWIPFDTNVSPYYEIAFAYQIVSALIYGLLIGTCDSF
IAGFMVHIKAQLLILKNSFGNYIYAAKVKTQVKNDFENLSFLKNCSDNGLKTNKDIKNLNPETLIYVQRYL
RDCIIHHQQTIKLVEIVESEFNYLMLIQFLGSLLLLCLSLFQLSINDIRSTRFFSMICFAGLMLFQLLIFCWNG
NEVLVESLEIAFAAYGSDWFLCDLATQKALVLVIQKAQRALQLSAGKFAYLTLETYMNILRASGSYYMV
LRKVNE
>AcorOR3
MDDENSNGNPDVGADFFKPFFSILKVILYWPLYTSKSQLLQHVHMLLSAVKVTFTVVLYIFLECFTVYKR
SNLQERLAGSFMMFTDVSYLTKVLFFIINRKKILLVFKAVSDDVFSPKDTLRKEMITTTMTNWKKVFYIYI
TNCCFTVTLWAIIPSLQNGSIVLPYNYHYPFDVTSSPAREFAYIYEAGILYLIVVSHVSLDALMVGIMAFISA
QLDVLNYNLRGLNNLDEVNSYSKHQFLKEEQIRCMIFHKKVMRLVKMLNQILAVPLVTQSCFGAIILCLS
LYKLSTLNPLSGEGLSFINYYFGMLIQLSVYFWYGNEIIWKSNELSRSAYQCKWVNTSKPFRRHLLFFMLS
TREPLKIYGGRVLELSLQPLISILKFSYSCYTLLKSV
>AcorOR4
MEKETLQYNAFDLERRILWFYGIYFSKDFRPQKLHYLRVIATSFVINTLVLGIVMEMIVDHNNLETVFQSI
YYIIVVIIGQIKTFSLYRSLSQFNSLEDMLQDVIFNAEISTGCTSISKAVDTYKIFKRIFWGTASFCASVYSLM
PLLSGDLSIPIWYPSESFKLYCQLFEIVCIWTIAASLLSIDVIIMGLIYFMSAQINTLNYNLRNATDRNPDYDA
GTQEKQVQDNLRICIRHHLAISEFVSKLEEIFKFGLLLQIFSSIIAISAGGVYMVFVTLTPSSFIILVSSMTMIL
AQIAMYCWVGQGLLTESDQIGESCYMSEWYTCNIATRKMFFIIMERSKRVISFKAGNFFELSFATLVMIIK
NAYSYFTVIITAFK
>AcorOR5
MVEMRFFDLNVRILKLSGLWVPNFTNKWKYRRTIAYNSICILYSMIYFTIAELISFKESAANLNDLVKNLN
MLMSFLLTLIKVIVWFRYRKDILKIIRFLETPRNVFKDYNLNNEEIILKELEFKDTWTKSFFIMSTLVPLSAG
ILSITETLTTGEKYVVFRNDSSLIYIQKLPYYSWIPFDHTSSKCAFRIAVVSQCIALLNCGYITVGLDMLFVA
LASSITAHFMLTKEAFRSIGNFGSNEFDNAQNDNYIKFKNCMMHLQTLIKICQRLEVIYSFLILMQVLVSLV
VLCTCLYLVSSIPVGVRLLGNELAYLLAIEIQVAVYCFVGNKLTDAALQIPLAIYECNWLNTTSNFKKAVII
TTIRMQKPIYITIGKFSPLTLNTFVMIGKTSYSIFTVLKSRN
>AcorOR6
MKRDITQYNALNFERKILWFYGFYSGKDYLARKHRNIPLIISSCFSFTFITGMVLKVLEYQDDLETIFETAH
ACITSITGAIKLFCLYRALPHFNILEESLIDPIFNLEIFEGSNFISKAVKEYVSFSKFYWTMVFITFVLYGVFPV
ISGEIPIPIWYPADCPKFYVQIFEVISILILSCSYPGIELVLFGLFYLMSAQLDTLNYNLTNSTQWNGEDNREV

QEEKIQDRLKCCIEHHLAIIRFINGLKDIFSFGIFAQIVLNVLIICTSALQFLRNSVTIVTLLSSIMYTLTILTQIG
MLCWVGQNITTKSSLIGESCYMSDWYTYSVSTRKMFFIIMEKSKLVISFKVGNLFEISFKTFIMIIRSAYSFF
AIVITMYK
>AcorOR7
MSLRDEKDPEILSDYFWFHKLVLKICGVSFKEQENLIYKIYSRFVQCLIGFLIFGEIYTIILSRNDLQLMAEIIS
VSTSHFLGLFKLSVLWKNRASIADALNALHTGVFLPNSRRSGLSEKILIKNCITKVYMLIAFHSATLVATVF
NIIGSSLVTKFKFDDYELWKMPWIPFRLFPITTTIVYYTIYVYQTVTLILFACIIITTDLMVVAVLVHITTQFH
ILASVMRTLVENNSVNYISEQYDLYLRKKLKYAAYYHQELIKLTDRFEELFNMLVLAIFMGNCIVLCFGM
YLMSSGELELNQLISEFTYLITVVMQIFLYCYYGNMITEASDAISFACYETDFVGTDLRFQKGLLLIMMRS
QRPIVLTAGKFAQISLAAFVAILRASYSYFMVIKSSSVEA
>AcorOR8
MEEEYVMFSGLYSLNLVGLHPFKSSFKKGVVALIFIVFTLICYALNLAGVILKYDGLKSLADSIDAVPAGQ
QVMVKLLSVLFLRNEMKHLYNTVEKKWDNKIYGEELENTIKKLSLKFKKIYNTYRMTIWVTAILYVSKP
LILFSRTLLTEMYIPCNLSQNYCYISFIAIQTIYIFDLAFVVYTFDGIFYAFLFYVYCELEKIKYGFANLNVSIT
TDLNNNEEDCYTKFCEIVKHHYSMIKFLQDVNKVYYLQLLNHFVTITATIVFGIFFMNMDGFPPALGKVS
RYIPYLMSYHFQLYIYCMWGQQVFDQVCSVCDVIYQSQWYVRYQPKLAKGMLLVMKVSQIQNKLTIGD
MWKLNLGTFMSVIKTSMSFHAFMQTVYKSDEVLVESYNQTIF
>AcorOR9
MTDRPKLLDFTKLPRRVLWCFGFYFGDDMEEHIFHRLICIISLTLTIPFPVMIMGKILLDLRHDLQALLETL
HYFFLHFWIVVKIMLFLYGFRRLRNLEAWLQTEIFNSYTRRQDHFLNKAMRKQMSFFKLLWSSTLCFTSM
FALFLPKDKVSLNIPIWMPFKMDRIFWHIYEVLCYVITASTYPAIDCIITGLVANMTAQLQILGDNLERIHL
DHEGGLLKCEDKIQERFKRFIQHHIAILQFINETEAVFSYSLFCQILFSVLGICLSGFQFLLVPPGNTKFMLA
CGYLTVMFFQIYFTCWFVEDLIIQGSDVVTSCYASEWYNYGSTTKKLLFILMERAKKPISFRAGYFFTLSL
ATFVMILRNSYSYFAILRHVYKE
>AcorOR10
MDSYIPNFFKVNVTFLKHGAVWSPKDKTQRSYKVYKMYQVIVLFLTLGCSSYSVVMGIIIHLKNFAILIEV
LSVSFTILLAAVKTSFWLIKGEKIKIIMNRLETDLFHCEKIDDFDPEGMLNQAKLSGIKYACLLLFFSHLVL
GLGYIPVMSLACWYYFKDLQITDVPTFKTLPYYTHIPFDHDTPLKYIFACLLQCVPMYLYVNAFVGVDSL
FMNLMNFIATRMLILQGAFRTMRKRCLQKIIGYDLAPDSLHNSNEMEEYMMSDMKKCIQHLQLLLRSCK
DIEDNFQYVSLVQALGTIYILCSTLLLLSTSPPFTKEFGRNIFYLLGVIVQLGLYCWFGNQLTLKAANVPIA
VWESQWLETRKPFKICMLLTMMRMKRPLLINAGKFVPLILDTQIAVLKGSYSYYTVLKGMSK
>AcorOR11
MAFYKISIKTSLTMLNLKGLNPLISTRSSNTRAITFLLTEVLATITVASSLFTKTLEADS
VVDNISGIVFSIQTICKEVTMLLCRDEFVALLNYVEEFWPVNEFGIESGTNIRNIQKSTSKALKIFRCLLLMC
TVIIISEPFFAEGRQFPVSWIDISCIQTSLICYGVIYGFLCGCTIGLVIFLSLIDGLFFNLLSYGYCELEQVKYAL
FNLSIDGDVRGDQVETLREIAVLVRHHVTSLEYVQRVKKLMSKVMLYQFSSSLFTLCTGLYVLTYQGFPP
SVEAAVKFVPFVACAAFQIFAYCVAGQKISEQTESIANAAYECRWWFKHQPRLQRSICLIIQRSHRRIVLSA
GGLWNLDMDTFIRILKASFSLLTFMQTMYIPE
>AcorOR12
MNSTSIYKNYFKVHFFVLTCLGIELKPMKGSIANFIYKIYAVMIFTVVVYLYFPYSEILSLVYEENFESAIYNL
AFLLTQILGLIKISLILIYKKKIRMFCKFIETPPFLPDQNRSGEAEFLYVKEAIRACNTQGYIFYGLTAGIISQM
MYDALSNPGYTKCFVDSATNITITKHIRALPFNSKLPFETIDSPYYEIACIYGSLSGAIFGYSVGAMDAIICGI
MCHIRAQLLILQECLKTFIPRGIYQMRENVKLTNNDQKLLQSITNNLNETIEIPNTLQKYVHIAVCNIITHHQ
KIIKLAQDAEELFSPLMLVQFLFSLGILCFQLFQLSITDIESVHFFGMSSYLILMLFQIYLFCYRGNEIMLHSH

NITDAVFESLWFLTDLKTQKLLLIMMIRACRPIKMTAGKFVFLSLEAFVSIVRGSGSYFMVLKNTNAPATE
L
>AcorOR13
MASNKTRTLCKNYFRIHYFVLMLLGVSIQPMEKNIFSYLYKFYSIVMFTIVYIYFPLAEILYLVYNTDLENI
TSGTTYICTHTLGTLKIILILIFRKRITAFCELIETKPFLPDPHRSGDIEFDYVQEAINACNYQGYTFHIFVVAI
VLPKIFYSLRDSEYETVFNDFQNVTFLVQRQRAGPFNCVMPFNTINSPYFEITAIYQASCAAILGCVIGSIDA
IICGIMCHVKAQILILKKSLGTYIQQGLFLMEEDNIDGKNVIGVDEFEMIRNSKTSIQLENVPISLQKYVDIS
VTQIIIHHQKVIKLSQDAEETFSLLMLVQFLFSLSIICCQLFQLSILKMGSAQFYSMCFYAMLMLFQIFLFCY
RGNEVIVHSYDLIDAIFQSNWTELNLKTQKSLLLMMTRACRPIRMTAGKFVFLSLEAFMSIVRGSGSYFM
VLRSINMPEE
>AcorOR14
MDNFFDVNFTMLRMSGIWIPDTSSQPIIKLLYLLYNTLWICYSCLFFCPSELVYFANTVTYVPDLVKNVNM
GMTHFLANIKVCLWFYHRKEIMAIIETLGIYGRRYESYGDFDTDKIVQNAKRFKDIFSVLFLNFAMFTSISS
CLICFLNVITAEIPPGEEIDMKLPYFSYVPFNYKASKVAFSIAIWYQFFPVFNYAYIIVGFDTLYTAILGYVS
AQLDIIQGAFETIRPRCMVRLGLKLSQNILRDPPTLMDEMHKEMNKVVNHLQVLLDICRRLEEIFTNVILA
QVMISLIVFCTCIFLVSNLPMMSLNFAAEMIYMIAIECQLLIYCVFGNKVTVSSGNISSSIYNGDWYSTSTSF
KRSMLITMSRMQKPIYFTIGKFTPLTLSTFLTISRASYSFFAVLKNSDFSN
>AcorOR15
MDYNYKNIFNLNLTILRVLGYFPSNTGSKPFNAMYKSCTCIAYFLALLFIASQAVEMVLMAKAQNLEKLS
TTCLKLFLNVSYFVKLTFFINNNNRVKLLIRKIEHKLVSPSSPQQDESMTKHIKCMTTFSRTFLYMSVITCV
LFAIFPLIDKNEELEEINTDGWYPFKSSNTVTLVAYVYLSLEELLAGLCNVSMDCIVIGCLSYICMQVRFLK
HNLKHMKDICTNTLAKIPDQSLLNKNDYRRQLQEHMDDTLINCILQYQTIVRIKRDIEEIFGMGIFIMFMFD
CLALCMTMFQLLIISFKSIQFFCVIIYMMCITMELMAYCWFGNELLVISSQVPVAAYESDWIDTPVYFQKN
LLMFITIAMKPMKVTVIHFSLSVETFTVIMRTAWSYFAVLRQKYNEEH
>AcorOR16
MTRREMNYPKNYFHKPLRNLALCGLWLYEPKKMNYKILHYLWFIILATCACFYLLTEYTHIIKHLHEMQ
EVTLALCYIFCHSMIFGKIVIFIIKKGKISKMVKLLESGPFLPNVARGGPEEIDIIRRTIHLTNVQLKIFGAVIV
VMMTTGVLPYLKNGRTFNEISPNITQVIVKFPYPSTLPFEVDYTASPCYELMFTFQVFSMNLYGWYFSNID
ALIIGLMMHIIAQFKILVSAIENVTKRAENMAAHDKSSALSQVIKKITFIKYNIDCDNFIIIERVETYSETIMA
NLKKCINEYAYYHQEVINLVDDMEKSLNFLFLIQFIGCLLTIVVGLYQISLVPFGSSSFTNMASFSFAITFEV
FMHCIYGDEISFYSAEVGKAAYNCEWIKADDRIRKNLLLMTLRCQRKCFLTFGKFSKINLVLFLSIMRGAF
SYFTFLQKMNEELNM
>AcorOR17
MSEVSFKSQKVAQYDTIDVSRKILWAFGVYTGKKYPNRILCKISLAINCILTFTFMISMLINILLNMDDLET
VFVITHLLVTELGYTTKTYYFMRMLKEFNALEELLEEPIFNDHSLEQDNFVAREIRTSKILSNIFRCLSWCA
QLTYTICPILDGDIWAIPIWVPLTDGSPKLIYQAYEALCFMSLASVEPALDLIPVGFISTMAAQLDILNDNLK
HSADKNEDELEEGKIRKRLAKCVKHHLAILSFLKKLEEIFSFGIFIQIFTSVGAICMSGLQFLVVPVRSATFV
AVFIYFWVMVVQIGTCCWVGQTLITKSNQIRDACYESTWYNCNTSTKRIFFIIMERSKKTISFRAGNFFNIS
LATFVMIIRNSYSYFAVLMQMYK
>AcorOR18
MDLLEKYSLRIMQLRSLNARESSIVNRAKAIGWVFVDLTFLGSTLYYLLFHVTDIIEAVDCITVIIVVCQTL
MKQLSLLIYQQEYADILNTVDKFWAYDKFGAAPNKKLTSIQNLIEKLVQCHMVVIIACGFFYYFKAALQR
EKVLIMGWVTVCGIENNMCYAVNYAGQVMWIAWLMPIFLGYDTMSLLLLGRVYCELEQIKYGFINLEV
QGEESEVLKQVSALVRQHNLVLDFLEKIGGLFSSILLCLFLTVLMALCTSFFLLTATGFPPSFSVLSRLGPYL

AGSCGQNLLYCIVGQIISDQTLSVADAAYDSKWFATKSLGLRKAICLVIQRSQRSTQLAAGGIFNLNLETF
VAVTKASASALAFLNTMYN
>AcorOR19
MRSEFSTYTNFLKVLLYWPIESKNVYIVIAYDIMSSIKFILVYIVFFIMDCIVIYENSANVEQMVSESFLVFTL
CNFFVKSFQYTNCNRRIRGVLEDISLDIFEPKDKQSEDVTKKAMAYSKRIFYIYVITCCSFGFLLGAGAIISE
EHNALPFNFSYPFDKTHGFGYKVALAYEALSISSNAMTHATMDCVVYSILAFVRVQLSLLNEDLCNLGSI
QYNTYQASLEQQILCIEKHQAIKRIVKELNEIITFPMFVQSTLSAITLCMSVYKLTGVELLSEEGISYIMYCA
VMHAQLGIPFWYGNEIISKSNELTISAYQCNWIEENKHFKSNLLIFLMSTIKPIEVSGGYFIVMSLEPLITILK
CSWSYTTILRSI
>AcorOR20
MLWVFGLHLLNFKETGFRLILFKIRTLITIVSSTTMLLFLLVKIIQSKNDLMSAFETCYYSLIQAAFVIKLYIY
LHYLPILIELENKLESNIFNGHKQDQLHFISDAIKSHQTYLGFYKICCVSTAIFYSIFPALDGQQLAVPIYSPL
DLKKYRLIVYLYEACNFFITACNNTAFDGTVIALITIMAAQIDVLKDNLIRATLRDQTVDAKDQERCIHKR
LKHCVIHHDAILDFTKTTQKIFSNGVFVQILVSVLGICMTGIVFLTVPLKSMKFISMVLFLITQVVQIGMFC
WFGENIRAKSSEIAQSCYMAHEWYSNNMSNKKILFIIMERAKVPIIFKANGVFVLTLNTFVMVLRSGYSYF
TVLRHISQNMDLS
>AcorOR21
MDTLEKYALRVLEARGLNPVKSSIMSKVNAIFWTIVDGTFVITILMELVSNTSDIFTFVDNFSAITVASQVV
TKEIGLLTHQQEFRTVITCLKEFWPKDKFGKQVKAKLDNIESFSKRFLQIYICSVACAVSLYMLKPILEGNK
ILPIMWVTFCSLEESLYCYIFNYILQVVWAACGLHMLVGFDCLFILLLLCGYCELEQIKHALISLDPDETTD
EDDAPLLDLIASLIEQHNRVLNLLKRIQDLLGSLLLLQFVATLLSLCASLFVLTSVDFPPSLPVVSKSLPYIFS
VFTQNLIYCVAGQVISDQTLSVADAAYASKWWIKAQPQLRRMILLMILRSQRPEEMTAGGVFALNLETFV
AIMKTTGSALAFMNTVYGEEE
>AcorOR22
MPVPKPLIRKLSAEFSKSMYKDDVKRCILPGKILLQGVCCWPDDETLFYKSLGWFLFWNLIIVEIFHAAYV
VKNYKDIEDAVTAGATVTTTMEGIVRLHTILTKRNIINSILVKVWKRFWPLDVVDPIKRIQLRKRAQLALV
LTSIFLASSIISNSQMVAVPYIKNRTMLLKSTFPFDWDQLYYYEIVYVWHYFSDWFVLFMINSFDFFFVAL
VTICSIQFAIMQEVFKLILSKQSLRHRAVIFGQRGKTMDDKEMLLKCLEQHQLLIGICNDLEKSFNITILIQF
FVSTSAICAASLLLKVDYSQFLKMLMYAAAHLSQLFYFCFVGHELSYESGQLSDAIYECNWHLSYDRDFR
KALILIIQRSHRVQWLTAAGMVKLDFATFLKIMRLSFSFYTLLHDMLMKNLDLN
>AcorOR23
MEKETLQYNAFHLERRILWFCGIYSGKDFQSQKRHFLRAMGVFLVLLIFDLSMLMKIIVDHNKLEIVFQTA
YHIIVIIIDQIKTVSLYRALSRFNNLEDMLRDPIFNTETSTRCTFIAKAVNNLINLKRVVWRMTIFIATLYSLM
ALINGNLPIPLWCPSIVLDMFNPYYQLYEVLCIWVTTATISTDLILIGLLYLMSAQIKTLNFNLRNVTDTNP
DYDAERQEKQVQDNLRISIRHHLAISDFVSKLEEIFSFGLFLQIFSSIIAISAGGVYMVFVTLTPSSFIILVSSM
TMILAQIAMYCWVGQGLLTESDQIGESCYMSEWYTCNIATRKMFFIIMERSKRVISFKAGKFFELSFTTLV
MIIKNAYSYFTVIITAFK
>AcorOR24
MLEVLYTLGEYTGVFPQKNKTDDYKNVLNILVVFYAAIGVFWFGVNVLMNSKATLLDMVDAIYTMESE
CSLLYYYMISIHTRKPALKLYNDVTDFTTFGKPKTLEREETRICKIYKIIVGYGIVAPTVTNGFYLATYDWC
MRSHRNDDDIQYCGYTFGIYYPYNFEKGVSFIIHTAINWYSFVFLSLVALTLVGYTICVARYLVLKIDHLN
TMLSEVLKNDAVNRRELLKKCIRYHKHIISLVDGLNELHSMNNAPAIFLYSTIIGVCLFYLTNEYNTKAIAL
ACGYIGGIFCLNFAGQMILEKSETVGVAAYNMEWYNADSSTAKDIMFIIIRSQVPLKYKAGPFGTMSLIFF
GSILRGAYTYMTMQTDIKEK

>AcorOR25
MEEELPHKIRRLSKDFNRDISNDGAKRIMIPGKIFLECLLVWPDREMKYITVFNWFMFINVIIFEITHACFVV
PNIADYTTVIAVLVTVTATFQFLVKFYVIVFKKSIINQILLNIWREYWPLSVLSPKKVKRHSSTCKVKLRLIL
GCYTLAVIFAAIITFAPFLTNTELIVKSIFPFQWNKTYTYELVYTWQFVTAWYITFLINSFDMLMISVVIISA
VQFAVLQNVVKNILTEKGERQRRYLYNKDISNQDMFKRWLEQQRMLIDTCNKLEEAFRIPILHQLFCSITG
LCASSVILKVDQSKFLEMSTIALANMFQLFYYCFASNELTLQSEKTSDAVYFCNWQISQDMKFKKALVLV
LQRCQKPLSLTAAGFIDLNFLSYIAVLRLCFSFYTLLTDLIVSKLEAAELQ
>AcorOR26
MASQQELSFLNRFKIFQINREIIMDGAKYLLIPGLVSAKIIMAWPEDVRSKSFEILVFVMSFIQCVTIITSIVL
NIVDVNTTIMMISAFEAVLQVVGKFSALIIKSKDLDKLIKTVRYEFWPSDITNKDTAEKIRKDSRILFKIMMI
ESSICIMVVLTIAFGPLLKTGRVLPYPTWYPFDTSASPVYEIVYILQSYFGLHLSVPPIIAYDMLYYSLCANC
TAQFRLLCDALRCIGNGTEDEMITKLVEFDESQKELRGTSEQKLLILCIKHHQRLINTANEISQAFGNGHLV
QLMGSASGICTACYILTSNPDLSSVANALVQYIAHVGQIFIYCAVSNELSYWSTLVPTAAYESLWYKKKY
PNIRQCLAILITRSQIAISMQAFGLFELNYTSFLSIMRFTFSLYTFLSSFA
>AcorOR27
PEDTSFNIYHGLILAACFLNIVSTTISVIVNTEDTDALVLKLFSIGALAGISVKYAALLYKSKDFTKLITIIRVE
FWSSDILDLDCDRSIYKDTKLLLVIIVTEYLAGCTCSSLMAIIPYIKSSKELPYSLWLPFDWTRSPYYEILYL
LQGYIAVFLMNAVFGYDSLYYTMCANCTAQFKLLCCAIKCIGTGTEHEIIRKLLNIPGLIHEWNPRISDEER
ILFICIKHHQKLIKMCNDINVVFGNGHLIQLIGSTIGICAACYRITTEPNFNDLLTCIAYYMAYVGQIFIYCAV
SNELTYWSSCVSIAAYHSLWYKKKYANVKQCLSIMMLRSQKPVSMQAFGLFELNYAFFVTVMRSTFSLY
TFLTKMATK
>AcorOR28
MTLNQNELQYTIINTERRILWANGVYTGKQYPRSITNKTVTIINTILTFTFLTSMLIKMILTKDDLGVVFEII
HMFITEVVWALKACYFVLTTKQFNALEESLKDPVFNEFTRDQKNLVSQQINKTKRIGNIFRNMSLTTCAC
YILCPILDGDVWAIPLWIPFTDGDPTIYYQLYESICFLSLASAHPSFDMLVIGFLNIMAAQLDILNDNLMNST
DRNEDEDFEVQEAKIKSRLRKCVIHHLAIVRFLSKLERLFSFGIVMQIILSTIAICVVGLQFLRVSLYSGTFL
GIMFYFWTMVIEIGLYCWAGQNIITKSTHITTACYTSNWYNCSTSTKKMFFIIMERSKHEIIFRGANFFDISL
TTFVMILRKSYSYFAVLVQVYK
>AcorOR29
MSFTVETLQKEDLIHVISFGTRILWVCGLYHCKHLKKHFAYNFARILVIALSLPFPCLIITGLVVSHSDLSGF
LELGMFFLGSSWISIETTAQIYSLRQVAEIEEMLESDYDYFKPKTELQCQFILAKRRRLVLITQIVWFCVYSF
MIIFVLCPLVTNASLVIPMWIPFGNKEFSAYLYQSFYLLILCGIYTLLHNTFLGPILMATAQFQILKDNLIHA
TDRSEDEDGFAQEKRIQERLKRCVKQHNAILKLVSTVQGMLAPVFLGNISFTILGICFTVLQIILATDSGNFI
LLTSYLGILILQMFLTCWVGNDLISETSDITQACYLSEWYNCTPSTKKMFLIIMSNTQQPISLQSIIFPVSFGT
FVMILRSSYSYYTVFSQVYD
>AcorOR30
MSTKTGKLEKLSLKFTQDIYQDGVKKCLLPVKVLLQSVCCWPDDELPYGKAIGWIFFSFLFINGVFNATYI
LMHGKDISEAVGASVTVTINFEALVRIYCILRNRRVFNEILVKIWKQFWPVKAVDDKTQAHLENKAVFAI
TVISIVLITSIFSNTFITTMPFLKYNQLISKSTFPFDWNKHFVYELIYIWQYFLNWYILFAVLAFDFFFVALVS
MCAIQFSIWQHVMRNILNEESKEQRRVIFGKMENEMTDKEMLRHCWQQHKLLNNICDDMESAFSITILLQ
FVVSTCANCAAFLTMKVDSSQFSKMLSFSMGHTTQLFYYCYSGQELMYQSEQLSHAIYECNWHLSYDRD
FRKALVLMLHKSQRIQCLTAANFTTLDFTSFIRILRLTFSFYTLLDNLVEDTAENGI
>AcorOR31
IIGIMQTAYVICLIITCSLIAFVAGCTEFIIIRIEHLNLLLKRVVKPNDLDSKESLIKCIKYHIHIISLVKKFNMC

FDKFTMLFLLQTGPTIALTSFSVIVEPKPSVIIHLAGWTMTLFIFCLSGQRLMDASTSIGDTIYDTQWYKMN
NSLGKYVILILIQAQRPLAMRMWLYSEASYMTLAQVMKLAYSITTLLNSTLQKD
>AcorOR32
RMLPMTMIIVCNFEDNSCFVFYYVLQICGLFTQLITLVGFDGLFFTLLFCGYIELEQIKNALVNLDRNGKA
GISDEKLLQQTIEIVEHHNFVLEYINKFDRLFQIALLVQFGITIFSLCSVLFMMTADGFPPSTSNLIRGGPYAL
SALCQILIYSAVGEKIVEQTEDIAQVAYEVDWYTCYRPK
>AcorOR33
LPPAAILSNTKISLKPLLVHKLLFKSSFPFNSQALYIFEIIYTWQYFVDWFVMFMACGFDFFFISLMSVCITQ
YIILQDVIRAVFSKESKKHRKIIFGERGINMTDKEMLFECLKQHKLLIRICSDLEEAITTTILLQFAVSVGAN
CIAFLILNIESSLFVEVFPYCGAHLLQLFYFCYVGQNLTHESGNLSVAIYESGWHLCYDLQLRKSLVLMIQR
SQQEQRITAVGLIELNLESFIKLLRLSFSIYTLLDSFLVVDDE
>AcorOR34
FLPMEFVKLITSYQNVKSTMEQLGVVTMHMISTLKIVNLYFKRNEISRIIDELHYNDLTETSGSLERKNLQ
NKFHRKIRRLCMFFFHMGNCTSTILCATSLIHLIICKHETVYQEFCSTVQPIVISTPIHIRSLIYSRWIICAFQW
MCMFLYGWQIVAHDTLFAAILIKIACNIRILQMDFKNITAESDQNITKMNYKMNQLTFQLQKLIRTCQCA
ANVFQYIILLQVLSSLFILITCLYVAASVPVFGIEFVFQLQYYLTVVTQLSMYCWFADEVTILFSQMPVSIY
QNYWICGDQSFKRSMLINMIRMNKPIYFIIGTVAPLNINVLVYILRASYSYFAIIKNK
>AcorOR35
SDNYCYTFFLVIQIVYVSAMVFTTFVFDVVFHAFLFHAYCELEKIKYGLQHLGISEDVDNDTIVYKKFCNI
VKYHNFTLKFLDKINDVYYLQLLNHFATFVAAIVFGIFFMNIDGFPPSPDKLSKYIPYLITHQFQLYMYCVL
GEIVYNQVNSISDVVYHSKWYIKRQSKLTRGMLMVMIVSRLKNKPTIGNIWKLNLATFMQVLKTSMSFH
AFMQTVYKTDN
>AcorOR36
MIKYHSIAAAQINILREKLRTLHTTLDDKFDENVRQGLHDCIRLHISIIRFLENINRVFSEVVLIQYISAMILV
CNFVMQLLFLVDPLSTQFAWSCSFLLVILTELLLYHWFGNEVVVMSHRIGESCYLSEWYMFNSANKRMII
ILMERAKRPLNVTVYKFTDVSLASFGTVMRWSYSLFAVMRNMYTKEI
>AcorOR37
DEFNRNISKTLAVRYKIVKFIQLGVVGISIVGVLAFFLRPVFISDVTFMLETWIFIDSNLLAGIVLMLQYYYF
SVIISVLLGYDFIYMSLCIDMISQMELLKHKISQILSDNIANVTLELVTCIRHHQILLSVYRRMREVYSLMLL
FHYFVTLIGTCTTFYEFLGKSDVPDFVINLVTVSVLFLQFGCYAFPAEQVALEFFDLSNFTYMSKWYECSIR
VQKLVLFIMTISHKELCFSGGGIMDINANAFGSVMRK

>AcorOR38
AKRTHFLYSTVQRMFVTLAILGIILYSFRPLATKGGLVFPSRIFVDLVGFQAVLLFSQYYFLLIIAAVVPGY
DIIYICYSAHVIIQIRMLKYKFEHITKNVEIETINSYIRHHQFMLNIFDRMKGVYFWMLFFVYSLTLITGCSQ
LYILILGNTQLSDLLASAVFITALFFEFGLYTFPVEEIVSQFTDISSSVYKSLWYERALEDRKVLLYVMMKG
QRQSYFSAGGLIEINVNTFGSVIRKIFSFYAILKNVLNK
>AcorOR39
KIASNTKLIYNYTKMVQTFLLCVFITSVHFYFLKPFFNSDDVFPFNVWINFNSLLLNVMVLASQYYCLCIV
TPVVLTYDVIYFSICLHVIIQLRLLKYKISRSSNNTQNELKIWVCHHQLLSSIFTRIQEIYSGTLLLQYLMTLG
MTCIQLYILNTGQLDVADTTELILYLATMYTEFGYYSIPVEEMSFEFLDVGNAVYESLWYETDARTKRSM
LFVMMYAQDLKYLNGGGLIRVNIDTF
>AcorOR40
SWTTFCDIDHSVCYAFNYIAQLLYVLWGLMGLLCYDIMIMLLLAAGYREFEQIKSGFLELSIDETVGEENI

KALEQIRALVKQHNLVLDFIDGIGSFLSKILMFQFIAIVFTNCSSLFLLSVVGFPPPASTTCRIIPYLACLFGQ
NFVYCIAGQLISDQSVSVADAAYGSKWWAKTQPSLRRAICLVIQRSQRRSQISAGGLINLDLNTFMAVTK
TTSSVLAFANAVFQ
>AcorOR41
MPGKKITVRKLSLEFTRDIYKDSVKRCILPGKILLQSVCSWPDDERLFYKAIGWFFFWSFLVVEIFHVAYIV
KHFRDISDAVLTGTTVTALLEALVRLYIILTKRSIINHILLKIWKQFWNVNVVINRITRNQLKKKARVSTILT
SIFLVSSIICSIKITSDAFLQNRGMVLKSVFPFDSTKPFSYELIYIIHYCTVWCGLFVINAFDFFFVALVTNCSI
QFAILQDAFKNILTGTSKGQRVAIFGQKHSNISDKDMLLKCLEQHQILIGICNELEESFNISILIQFVVSISAIC
AASLILKVDSNQFLKMVMYAAAHLAQLFYYCFAGHGLSYESDKLSDAIYGCNWHLFYDRDFRKALVLII
QRSQRVQYLTAAGIAKLDFASFIKVMRLSFSFYTLLNSLLAKNI
>AcorOR42
YNAFDLERRILWFYGIYFSKDFRPQKLHYLRVIATSFVINTLVLGIVMEMIVDHNNLETVFQSIYYIIVVIIG
QIKTFSLYRSLSQFNSLEDMLQDVIFNAEISTGCTSISKAVDTYKIFKRIFWGTASFCASVYSLMPLLSGDLS
IPIWYPSESFKLYCQLFEIVCIWTIAASLLSIDVIIMGLIYFMSAQINTLNYNLRNATDRNPDYDAEKQEKQV
QDNLRICIRHHLAISEFVSKLEEIFKFGLLLQIFSSIIAISSGGIYVVVVPLTPSSYLLLGTSMSVVLLQIAMYC
WAGQGLITESDQIGESCYMSEWYTCNTATRKMFFIIMERSKR
>HoblOrco
MMKFKPQGLVADLMPNIKLMKFAGHFMLNYYAENSGAVHTLRLGFCFGHLFLMLLQFGFTFGNLVQES
DDVNDLAANTITVLFFTHCIVKFIYFGVRQKLFYRTLGIWNQSNSHPLFLESNNRYHQLALTKMRRLLIIV
MVGTIGSWIAWTTITFFGDSVHNRKDPNNENETITEEIPRLLIRSWYPWDAMSGIPYYVSLIYQIYYVGFSM
LHSNLLDSLFCSWLIFACEQLQHLKEIMKPLMELSATLDTYVPKSADLFRAHSASSQDKLTESDYNARNE
DAHMRAMYSTHQEMGVTYRSGQLQEFSSGGIGPNALTKKQELMVRSAIKYWVERHKHVVRLVTAIGDA
YGIALLLHMLTSTITLTLLAYQATKIDGVNKYALTVLGYLFYALAQVFHFCIFGNRLIEESSSVMEAAYSC
HWYDGSEEAKTFVQIVCQQCQKAMSISGAKFFTISLDLFASVLGATVTYFMVLVQLK
>HoblOR1
MRRLSFEFTKAIYNDSVKKYMWFGKLLLEMLCAWPSKEGLFKRAIGWLFFCNLAVVEIFHAAYCAQNYS
NIGDAVMVGATVTTTLEAMVRFYIMLSKREIINKILTKVWKKFWPVEVLDPVKRKKIQNKAKFRFWLNLI
FFSSSLISSTNVNLAPYLSDNELIFKSSFPFDWNKPYVYEFLYVWQYFSNELVVFMINAFDFFFMSLVCICTI
QYIIMQEVFANILSKDSRRHRQVIFGDRGKHMTDKEMLKKCLEQHEMLIEICNELDNSFNRAILIQFVVNT
AANCAAFVTLKFDRSQFTKMLTYAVAHLSQLFYYCLAGQDLQYESQRLSDFIYDSDWQSSYNRDFRKSI
VLMIQRSQKPQCLTALGLVNLDFLSFVQIMRLSFSFYTLLDGFLADE
>HoblOR2
MSAGNTTKPVIRKLSLEFSRDVYKDGVKMCILPGKVLLQGVCCWPDDEGLRMKIVGWFLFWNLFVIEIF
HASYVFLNFNDIGDAVDAGATVTTTMEGLVRLHIMLTKKGVINSTLVKIWKQFWSLDVIEPVKRKKIKRQ
AQTAVMLTSIFLGSSIISNSQITGTPFIRNRGLVLKSVFPFDWRQYYFYEIIYIWQYYSDWFVLFMINAFDFF
FIALVIICSVQYVIMQEIFKYILTDESKRHRKIIFGERGETMTDREMLFECLEQHKLLIGICNELESSFNIPILIQ
FFTSTCGICAATLIMKVDYSQFSKMFTLVGAHISQLFCYCYVGQHLAFESDYLAYAIYDCGWHIDYDRSF
RKALLLMMQRSQRTQRLTAAGITELDYASFLGILRLSFSFYTLLNNLFMKNVGQ
>HoblOR3
MNSKKSENYFKVHFFVLNLLGISMNSMKNPIKNLLYKTYSLLLFLFCFVFYPISELVFVLCSGNVEDSTFVF
SFLLTQILGALKLVIVVIYKRKIISFCKSIETKPFLPDQNRSGQMEFMHIENAIRTSNNQAYFFHSFVVIVISQ
RYFYTSRAPGYYESTFDQTSNITIVKYVRMTPFYTILPFEQINSPGHEITVLYQVISAAVIGFMIGAVDAIIGG
ILCHIKAQLFILKDCLASYEQRAVYLMNKDGIYIDNREFAKHEMNKFNYSGNVRKLPSVLQKYVDVCVIQ
LIKHHQKIIELADGAEDTFSLLMLVQFLFSLLLICFQLFQLSICEIRSTKFVSMICYFFLMVLQLALYCWYGN

EIMYHSSSLSDAIFESNWIVANAKTQKSLLIMMMRVYRPIKMTAGKFSVLSLESFMAIIRGSGSYFMVLKN
TQSAV
>HoblOR4
MNNEYVMSFCLYHLHILGLNPFKQSIFSKLRAGFVALITCVLYGFNLAALFHDNNGIKSLQNTIDALPCGQ
QTSVRLLYLLICGNKMKQLYEMTNETWPNDICGSKLKERLQFDSERFKSFFKSYSTYLVTIVIIYLITPSFYT
YRRLTTEWYLPCKLESDMCYLSARLAQVVYFMLIIYQIIIFDGMCWAFLFCLFLETEKVKYSFKYLNIEEL
NSKNEVNRKFCSIVKHHCYLLEFLKRFNAVYSLQMLNHFLTIAAALCFGMFLVSKDGFPPTTFENARFIPY
VIGYNTQLYICCYSGEVIYSQLISIADAIYESMWYLNNHSNVSNGIRLGIQVSQIGNKVTIGGLWPLSLSTFV
SVFKTSVSLNAILQTVYINAERSI
>HoblOR5
MSDNFFNVNFTMLKMSGIWIPDSKSSLIVKIMYWAYNFVWIGYSCLFFCPSELVYFATTLSNLEDLVKNV
NMGMTHFLANIKVCLWFYYRKEIMGIIETLGAYGRRYESYGDFDNEKILKKAKKFKDIFSVLFLNFAMFT
SISSCLICFYNTMELDLPKGGRIELKLPYFSYVPWDYRRSKILFSIAIWYQFFPVFNYAYIIVGFDTLYTAILG
YVSAQLDIIQGAFSTIRPRCIVRLGLEVPEDVLRDPPELMNEMNKEMNKIVDHLQVLLDICTRLEEIYSYVI
LAQVMISLIVFCTCIFLVSLLPMFSLNFAAEMIYMVAIECQLLIYCVFGNKVTLSSSNIPNSIYIGDWYSSNIS
FKRSMLITMSRMQRPIYFTIGKFTPLTLSTFVTISRASYSFFAVLKNSEL
>HoblOR6
MVCPCEEILSSPRWILELLGCWPMKSPTKTYTIKMFVASVFTIAFPTMLCFEILFVYKNYNVLMQILNFLIP
YVYVLCKYTIFTFQQKQLFILLNCTKSKLFNQSPSNDKILRPIADVIIMVTKVFRFTLVFALLMIALLPLSET
KLYPVPISHDLGRYSILMYVFQITCLSIGGWLCMGFDCGFIALAAIGIGQIDILKLRLSSVIQDLVVNKNTFS
KSVEQLKTQTKINKALRACILHHFAIEEYCKRLSNLFSTTIFIQYLMSILALAITMSRILTITELSVKILELVFI
LMFIFTQLITYSWSGHQIMIKSSEIGEACYMVKWYYYDASNRRILFLIMERAKRPLYIKASMFAKTTLTTCI
NILRCTYSIFTILSMSYREN
>HoblOR7
MKGHSENFFDINLIFLKYSGLLPPKNKSNISYTLYKILRFFAVIITVILGTTGAIIGVIENIYNFNVLIELLNVA
LTMFLSAIKSVFWLINSKSIEEIMQTLATNEFDYEQTEVFKPNILKEKARRIGRNYTLILWILTQLTLGFAYI
PAISLSFWYYVNDLPITNITTFQTLPYYIHIPFAYNTAMKYCLACIVQAVPCYMYANILVGVDTLFMNILNL
IGTHMLILQGAFKTMRKRCLKKIAGPSLAPDGLHNSNEMEYYMMLEMKKSINHLQMLFQCCYKLEEIFK
YVSLFQALGSVIILCSSLLMLSTTSCVSKEFGNEIFYFIGINVQMGLYCSFGNQVTLKALDLPQAIWESEWL
ECRKSYKICILMTMMRLKKPVYLTAGKFAPLLLNTHISVLKGSYSYYTVLKSMDK
>HoblOR8
VCNMFAKTYRFFVGITIAFYAIFPFIEHTLPLPGWFPFDKKKHSYSLYVYHLVCLILNGYNHTSLDCINAA
MISIASAQLEILKDNLHNLRRNEDDDLTIDQEDVVVRKRVKKCVLHHNVIVKFVADIEDMFSNVLLVQFL
CSIIIMCVTGFQVFVIPPARMHYITLVLYFCCNLTQIAIYSWFGHDILAKSSDIGLACYMTEWNVLSPKSRK
LIFIIMERSKVPIILTAGKFINLSLNTFMMIVRTSYSYLAVLQHMYKT
>HoblOR9
MGHINCLEPLIKPKWLLIKSGLWPMENISVLIAIKYFLNFALDVTILGIILQNIVNAISTNNMNLLNWSICVL
LPLTNYYTKAVTLFVNKKHLRSILNDLDSDIFNYNSEKLNRHIRKIYAISNMMIRYFAFVISTFVFVFGVLPI
ITNIRPIVPASFKTGKFTRLYNVAHLFAVIFVGYNSTAFDVLFMSLMALCGAQLDILKERLSNVFVDASEIY
EDASSNMKFTDINVVVGCILKDCVALHEAINRFVMKLDILLSFPLLTQYGIGCFIICNTILELTIMGHLDSAN
IIGLCGYSGVVFAQIAVYHWLGNEILYKSDEIIQSCYLSQWYKLDLKSQKCIMLLMERAKRSLVITLYKFV
FISLESLGVIVRWSYSIFALIKARYK
>HoblOR10
MNTNQFKQINKDYFKIHFFVLKFFGISIKPVEDSLSGIAYKIYSVILFILCFVCYPTSLFFFLYLNADLENATL

VLCFLLTYILGTFKLIIILIFKRKITTFSAHIENEPFLPDNRRSNDREFIYVENAIRTCNNQGYIFHTFVVLIVV
QRLYYAAIDAGYYKNFWDFSTNTTIVKHVKSTPFYTVLPFEPIDFPYYEITVMYQAYCSATYGFVIGATDA
IICGIMCHIKAQLLILKNCLKTYIQRGIYIMKEDKVHINIQELEKHQELNKDSVYAVPLVLQQYVEISITELIK
HHQKILELAEGAEDTFSFLMLVQFVFSLALICFQLFQLSIYSIPSVKFFSMFSYLLLMLYQLFVYCYHGNEV
MLYSLSIIDAIFESNWVMVNVKCQKSLLLSMLRASRPIKMTAGKFVYLSLDSFMSIVRGSGSYFMVLTNM
NARKH
>HoblOR11
ILYSVASRMRKMFSVMLLFHYFVTVATTCSDLFILVKYVDITVIGIKFTVLVFIFGQFAYYTFPSDEVACQF
SDIARALYMSKWYRTNIKVQKSLLFIMMKCQRTKYFTGAGLMDINADAFGSVVRKTFSFFTIMRNILNK
>HoblOR12
MLLNATITKSVSYLNFKGLNPFISRSKSAKFKAIIFLFTETMATISIALCIFVSSLGSDSFMDSVTGMICATQT
ICKQIILLLFQDDFIDVVHYIKEFWAANKFGKEPAKQIDVIHNTTERLLKMFKYLLSIAAIAFLVKPFLEEEL
VLPVSWLSVCSIHESMICYTINYISQCFYIGDLIFSLLIFDELFFKLLTYGYCELEQVKYALSFLKTKNTTSEK
ESKIMLEMVIIIKHHSRALEYIDKVDKLFSMVLLCQFSTSVCGGCMGLFVLTYEGLPPKLNTALKNVPYVL
GVLSQIFIYCVAGQKIAEQSESVADMAYNMRWWIKHQPQVGRAICLIMQRAYKRKQMTAGGVWNLDMI
TFVAVRSVVMSNRN
>HoblOR13
MSTSRLSLRRLSLEFTKDIYKDGIKICLLPGKILLQGCCCWPDSGTLYSKIIGWFLFWNLIVMEIFHTAYVV
KNFRNIGDAVSTGATVTTTMEGFVRVYVMVTKGHVINTILVKVWKQFWPLDAIEPKRRQKIQRKAQLSV
LLTSILFFCSASSNSLITSTPYIRNHGMLLKSVFPFEWNQLYVYELIYIWQYYSDWFVLFMINAFDFFFVAL
VTVCFLQYVIMQDVIKFILSEKSRSQRIIIFGKRGQSISDKEMLYKCLEQHKLINGICNELEESFNIAILIQFFV
STTTLCAAALIMKVDYSQFSKMLTFAGAHLAQLFCYCYVGHQLSYESTSLAYAIYTCDWHINYDRNFRK
ALVLMIQRSQKVQNLTAAGVTELNFTSFVGIMRLSFSFYTLLNNLLMKNENE
>HoblOR14
MGKIMQIFSRFLRNLDILKRNSDILNDDGKCTLIVSEFVLCLVNFWPENHTIRTRITFFTLLAAYISMQISFF
MFLVTSIEDIGSFTKIMSSLSVGMQVIAKMSILFFKYNELNAIIRIIRYEFWPATTMGKLIKNQIRSKLTFILIG
CIAIYLLGMLFLLQIAAAPLMKGIRILPYNSWYPFDWSQSPTYEVLYILQSFMIIYVTENIVVGCDFLFFSICF
NCTVQFNLLCEVFKEIGNGREMELLNYLFRLPGTFYRPTMNEVDVNERKLMVLCIQHHQKLLNICLQLNK
LFNYGHLYQFVASVTGLCASCYLITNENSANGRSLVASYFVAHASQLFQYCAVSNEVTHASLELSTAAFQ
CGWHKKEYSDFRKCLSIVLQRSQKGVPVKAAGLFVLNYASFVTMMRSTFSLYTFLNNFTEQ
>HoblOR15
MSKRIIHYDSINFERRIMSVFGFYSGLGFEGNSLTKLVFKISITSGILFIYTMIIKIFLVTNDLEVVFEIFHILITE
TAFVLKAILFYQALPKLYSLEELLRGPTFNQHTEDQDRFISKAIFTYKLFARSFQFTSWGAMITYSLCPIIDG
TILAIPIWTPFDEDKYKIYAQFFEIICFIVLSTIDVAIDCISTSMINLMAIQLDILNDNLRRIGQNRSTGSYLEQE
KQIQNDLVRYVQHYIAIIRFVSDTQDIFSVGIFIQIFTSVVAICTTGIQIALRTSGTFISTLLYFQTMIIEIGMFC
WFSQDIITKSSQIGESCYISEWYTCNTSTKRSIFIIMERAKKEIKFRAGGVFEMSLTTFVMILRNSYSYFAVL
MRVYKN
>HoblOR16
IDAAQNGASIEKINAMIATLFLIVTCCLKIGVCTFQSRLFFSIVEDLHSIRFNSHDPKYNKPLEDAIQLINFFA
KVFRVCVFMIVLFHIVFPLFGRTPLSIYPTFELENHPYISHGIQIITVGIAAWNLTCVDLFFGAMITIAITQIDI
LRLKLQHFTADAKQEFLKRRKRENCSYHTISNEILVDCVDLHIAVIRFLKKVEDMVTIILLVQYSAGVVIV
CNNILQLTKSDSIFSPDFIQPFSFLCGVILDLVIYHWLGNEIIAKSDKIGEACYTSEWYICNAKTHKTLFMIM
EKAKKPLNIKIYKFTDLSFQSFVAILHAAFSVYTLLDTVAEEN
>HoblOR17

NNIDNLSEILPVFLAVLLLIVKRINIASRSSEFLLTLLVLEKTEFNNHPEGLMGPIKRCVRISKTLTKIFKVVM
ICVIIFYVMLPFVEDKPLLLPLSFDLGRFEKATCIFQVISFSYCCMSVVCVDLFCALLLSLATAQLEVLELRL
RNIVSEAKFSNGSSILNYKRDLDQRVYKILRKCIKHHLSIISLVRQIEKTYTVPLFLQYTLGIGIICFLLLQLS
YTVQPGTLQFLLLCNCLVAILNELLLYHYFGNELIFQSLRIRDACYMSEWYECSPFIRKMLVIIMERTKIPL
AITVYGYCLVSLESLSALFRWSYSIFALLSTVYWKVNQ
>HoblOR18
MLGIFGLYSRRDDENGVFFKIRRALIAVFSTVMLICLFIKMYENRNNLLDIFETCYYFMVQAAFVIKLYIYF
YHLPDLHALEEMLSGKIFNSLSKEQEMYISDAMKSHKIFAGCYQFCCVCCAIIYSIFPAMDGQALAVPIYSP
FNVEKYRMFLYCFEVCCFFMTACNNSSFDGMTVGLTTIMSAQLDVLKDNLKHAADRDMGGKFLEQEKK
IKDRLRNCVRHHNAILEFTAKTQDIFSTGVFFQILASMLGICLTGLQSLMVPWGSVQFASVGLFLVTEIIQIG
MFCWFGENIIIKSSEIELSCYMSEWRSCSTSNKRIFLIIMENAKTPIKFTAKGLFILSLGTFVMVLRSGYSYFA
VLRQVSQK
>HoblOR19
RISRASCKKRNELRICIKHHQLLLSIFGRMQLIYSWVLLLQYLMTLGMACVQLYILNTGELDIADTIELVIY
LGTMYTEFGYYSIPVEEISSEFSGIVNAIYMSSWYEKDMATKRILLNVMTTAQVPKYLYAGGMIQVNMDT
FGSVFRKSFSLYLVLKNLVK
>HoblOR20
MEEEYVMSFGLSFLRAVGFSPFNPGLLDHIKSSALILTTTFIYCLNILPMIIEYDGVQSLESSIDSIPAGEQVM
VKLLTTWILRNKMKYLYEMVNQKWPDDIYGNEFEIKLNNNAKNFKRIFALCKAQFMFSSLIYTSKPLFVQ
ARVLATQLYVPCDLLSNSCYITFLGIQNIYIVIVATGEVVFDFIFHAFLFFAYIELEKIKYGFRNLDIMGKKE
NNEKIVYSEFCKIIKHHNFILEFLHNLNNIYSLQLLNHFITLSANIVLAILFMNKDGFPPSYDEMARYIPYLIT
ILSQLFLYCLAGEGIYSQLASVGDAIYESKWYNMKQSSSLTKGMILVIQISHINRINRKLTIGNIWPLNLATF
MSVVKSSFSLQACLQTAYNTDDTM
>HoblOR21
NCTALIRFLITTIHLTYCKEDTIEKELCVTVKPLLIPTPGPLNTISGRWILGGFQCICLILYAWQIIAYDTLFAC
FLIYVDCNVHILRYFFETITERSINKLQLRCCSTPNLELTQQMNIQMRTGTINLQNLIITCEEIMDVFKYVILL
QVLFALFILMTSLYVAASVPVFGASFTYQLEYYMTIVTQLSLYCWFGNEITLSFTKIPDAIYNNNWVVSNK
SFKRSMLINTIRMNKPIYIKIGMVAPLNLNVLIYILRASYSYFAVIKNKY
>HoblOR22
MANIKCIKTITTPIWMLRKTSFWPEQTYTKTFILTSIFNNIFHFTIYTLLILDIINALKSNDIKLFNWVIGILIPIT
NYYAKAVTIIYNKKCLFSILDDFKSNIFNSHTEKHNLHIQLIRKVSHLIMRYLIITMGIFVLIFSVPSFFTNGL
LFIPPPFEIENFDVLYKFVNLIAACYIGYNCGGLDVLFLTFLALCVAQLQILKERLKNILEDAKKINVESKYK
MDLDIILADILRECVLLHKKINRFVEKLGVLLSLPLFVQYVSGCVMICFSIIQLTLGSKRSALDIIGLCGYSS
ATFGQMSAYHYLGNEIIYQSNEIVESCYLSRWYELNPKFQKRILLLMERATRPLILSAYKFVFISLASLGVI
VQWSYSLYAVTKAKYG
>HoblOR23
SKMSSDEFNGGSEVCDSILPYYIVIPFDISTKISCCFALIFQILPFVVFAWQITAHDLLFAVFLIALKCHFIIVR
GAFETLRSRCLKMLNLQPHYNILHDRDNIKLDNAMKTEMRKCTQYYQIIIKLAQEVENSFTHIILVQVLIVI
FIMVSCLYMMSSLVITSGKFFAEVNFVIAILIQITFYCWFGNEMTLASADICSGIYDNNWLSASESFKRSMLI
QMIRSSRITYITVGKFSAVTLLTLASILRGSYSYFALLSNLKE
>HoblOR24
LRNAATTEIYACVKHHELLLSVFSCMKKMYFWMLLFHYAVTLITGCSQLYVILVSSTDTADLIGTLIYVN
GMLVQFAYYSFPAEEMVFELRSVSVAVYMSMWYEKDASVRKTLLMIMMKSQQQEYLSAGGIIDMNVN
AFGSVIRKVFSFYTVLHNVLSN

>HoblOR25
MKLHCLVTKNGQIRKILKEISGDVFQPKSEYQEQLIITSLLTIRRCTKLLIYISLGSCVFFVVLPLVNRSDEKP
LPGKLWFPFNTSNHPVYDILYIYETIAVILHPLTHICMDTIPLALMAFICGQFDVLHDTLTNLKIFATERLES
NTKRTKDDLHEEMNKLLIECIIRHQETKRLANEVNDVYTSHFFAQLVNCVSVLSLIMYKLTMIPIASVGFF
TLALYLTCLLGLLLLYCWYGNEIVSKSSAVYLAAYSSDWTGCPISFQKNLLFMMLHLQRPVTMYAGRFF
PLSLDVYTAILRFTWSYFTLLLNIDE
>HoblOR26
AVLTVLFFGILDSILSRFKSNDYNDWSMPLAPFSLFKVTTNTQFILVCLYQSTCLLICASIISSIDLLMGSVLA
HMKTQFTILKNTIRSMQSNDDLAEEVTERHSQKTSVDLLQNKLQYVVTYHQSLIDLAHQFEDVFSFLLLT
LFIGNFVVLCFTMYHASLYPLSNTKALMDFAYVIAICVQLLLTCYWGNELTLESESVAFACYDVKFVGTS
PNFQKELALMIQRSQRPIVLTAGKFVNLSLNSYVSILRMSYSYYMVL
>HoblOR27
MSMSFILTSFKVLVWFYYRKDLLKITKLLEENSSRFKDYNFDSEDIISKEKLFKDLWTKSFFLASSLVSVSA
GFLSTTEALTSGEKYVEFKNGSQYFYNQKLPYYSLVPFDQTSSKLAYIKAVVYQCLALLSCGYITVGLDM
MFVAIMSLTNAHFILLKNAFRRIGAVRIERLNITSTICIEQSTMLRRACENELNKCIKHLQMLIRICQSLENIY
SPVVLMQVLISLVVLCTCLYQVSSIPFGAKLLGNDLAYLLAIEIQVVVYCYVGSKLTHSALDIPSAIYESSW
LTASLTYKKTMIMTMMRMQKPIHITIGKFSPLTLNTFLSIGKMSYSIFTMLKSRN
>HoblOR28
VKTLQSLMHFTHIFLKYLFLMYYKVDIEKILVDILKYFWKTWMFKKQIAEEVEKIYKTVESIQKCTIFLAAI
VLLVHTLRPLFDKSHIYLLETYTPSSYAIGALLLISQCYTLYFGFCVAIGFDLIYFSLCVHLILQTRLLKEKLR
EVFENSRDNSSFKLGYCIQHHQFLFSLFLRKKAIYSLMLLFHYFVSLITGCSVLFEILSSNTDLGNAIIELTVI
VFFMGQFGFYTFLAEQIAFEFSDISDAIYLTQWYNSNIFLQKCMLHIMMKSQRIHYFSGGGLIDINVDAFGS
VLRKTFSFCAIMQNLINK
>HoblOR29
MVPPPFDTGRFNIVYIFFHSLSVMYLCVNSTGYDVFYLTLMALCVAELNILEERITNVLEDVSEGHDSREKI
SLENIKPLLKRTLRECIILHDAIIAFVKKLSIVLSFPLFVQYTCGCFMLCNTVLQMTIVSDKVTESCYLSMW
YATDTLSQKTLMLIMERAKRSLILQLYGFVNISLDSLGVIIR
>HoblOR31
YCFMGGLIVQLGGIFTYETCMKNSVKTNKDMTCGYGYKVHYPYEYQSGISQIAHIAVGVYSLSFVAASAS
LFLCLFICCVEYITVRIDHLNDMLLKVFEVEDYEDQSFTMKLCLEYHILITGLAEQANSCLLLILSTAALFNY
SSTIGLSLFAMLKGNITKPLVSAIGFTCSIFILSAYGQQIINKYEAIGTAVYNSKWYHSSPLLRKHVLLIVMR
AQKPLLLQVGLGFYFHLPLFT
>HoblOR32
LLILHFYYFCIGFPVVFGFDLIYFALCVHVIIQTRLLKRKLKNVSRNCGKNSVSELNSCIQHHQFLFSMFLR
MKDVYSMMFLFHHFLTLISTCSTLFEVLNGHNTDLTNYFIKFVLILLFISQFAYYALPAAQVASEFSEVSDA
IYISEWYNKEVVLQKCMLFIMMKAQRIHYFSGGGLMDINTDTLGSVIRKTFSFYAIIRNLINK
>HoblOR35
PFEIDFSIKLLIQIMDFFYVLWVGITCVLIGFVVGCAELIIIRVRHLNIMFKDALSFASCVNSKKLLVTCVKY
HAHIISLTDRYNKCFDKFVMLFLMQTGPTIAVASFSIIIEPKISVMLHLSGWVVTLFILCISGQRLTDESLVIS
QSVYDTEWYQIDISLRKYVILILMQSQRPLRAKMWMFSEASYMTFAKVIKMSYSIITLLNGTLARKN
>HoblOR38
KKLKKLVEKMNEILAVPLFIQSVLGAVILCLAMYKLTKVAPMSGEALTYINYYIAMLIQLSVYFWYSNEIL
WKSDELSNSAYQCQWVDKTTSFKRNLLFFILSAHSPLKIYGGYFVVLSMDLLIAIVKFSWTCFTLLKSM
>TcasOR1

MMKFKVTGLVADLMPNIRLIQASGHFMLNYHADNSGALHTLRLGYCCMHLVFVLVQTFSCNFVNLVLE
RGDVNDLAANTITVLFFTHCVTKFVYFAVRSKLFYRTLGIWNQPNSHPLFVESNNRYHGIALKKMRRLLY
IIIIWTSFSAIAWTGITFVGDSVHNIKDPENENLTITEPIPRLLVKAWYPWDAMSGMPYYITLVFQVYYVFFS
LAHANLLDSLFCSWLIFACEQLQHLKEIMKPLMELSATLDTYVPKSADLFRAPSATSQDQLIENGTNPAKK
NEDLKGVYSTRQELGGHFRGGALQNFGSGGVGPNGLTKKQELMVRSAIKYWVERHKHVVRLVTAIGDA
YGVALLLHMLTSTIMLTLLAYQATKITGVDKYAATVLGYLLFALAQVFHFCIFGNRLIEESSSVMEAAYS
CHWYDGSEEAKTFVQIVCQQCQKAMSISGAKFFTISLDLFASVLGAVVTYFMVLVQLK
>TcasOR4
MYSENKFDFVLTIKPNILFLKIVGLWPVDNNDVYRIYTLIVTVFFMGVFDFTRIMNIFFVYTDLKVLTATIY
LTVTDITVLVKTCLFMSNIKTLKRLIVTINCDVFQPKTDHQKQLVQSGLKAWKVSYMVFWSLVFCCLVM
WTVSPIIQATPAGRKPLPLPAWYPYNTDITPFYEITYVCQVISMWFLATANMNMDSLIAALMIYVGAQCDI
LSDNLKKMKTFSKIKEEQAKFNQTLIDRIEHHKKILQFAYDCNASYNFIILAQFFTSSLAIALSMFQLTLVDP
LSMESFPLLSYAFGMALQIFLYCWFGNEVEAK
>TcasOR7
MNKLQKFDWKATIRPNIAFLHYLGIWPEGEEYYKLNFYTLKTILYIIILVISTIVFQVINIFFTLDDLTSLTAN
IYVLLTEILYFIKLCFLVKNMPALKLLMKTLDHKLFQPKANQIVIIQPLLNFWKLIFLAFVITCSFTVLFWAI
FPILDSSEEEKRLPLLAWYPYDTKISPNYELTYLHQVASYIYICYSHLNIDTFITALNTYIQCQFDILCDNLK
NIKSDTKNVDTKLAKCIKHHLLILMFANTSNEFFSWIIFFQFTSSAAITGMTLFQLTVVKPFTTEFYNFMAY
VTAEVVQIFMYCWFGNEVQVKSSNIPYAAFGSDWTEFSPNKQKSLLFLITRSQKSVKMSAFNVFDLTTDS
FILKSAWSYFALLNQVNSZ
>TcasOR10
MEDFSWEATLSQNINFLKVCGLWPPGDEAYKFNLYGIYAGFCVLGFLCVHTGTQTFNVYFILDDLEAFTS
SIFVTFSCVACVFKTYYLLKNMKLLKVLFININKEIFQPKNKEQQLLIQPSILFWKRFYLVFRILCYNTCFFW
CAYPILDKRIKQHKLPFLAWYPFDSSVSPLYEITYFYQAVAIWYIVIISFNIDMLIGALNMFVGAQCDILCD
NLRNLGKSDINELNPDLIKCIQHHKAILSFVSKLNIFFNWIVLLQFFSSAVSVGFTMFELTLVAPFSGQFYSFI
CYGSAITTEMFIYCWFGNEIEIKSSKIPYAAFECNWVGTPLGVQKSLIIFTIRTQRPMQVSALNLFYLSLDTF
KTVLRTSWSYFTVLNQVHSZ
>TcasOR11
MEKYDWMQAIKTNILILKIVGLWPDSEDYKFDFYALHASVWLSTLLVASTFFQGINIIFILDDVKALTGTA
YVLLTEILAVIKTYFVVKNMKMLKHLMQSLNNNKLFQPRSHEQIKLIQPSLKFWKLLYNLFHSLVGGATL
FWILFPIVDKKEKRLPFLGWYIVDTKVSPYYEIVYGFQFCSCCYMSALIINIDTLIAALNVYIGNQIDILCNN
LRNLKAGCSIERDLITCIKHHQEILNFVQYANKFYRWIILLQFFVSAVSIGITMFQLTIVVPLSSEFYSFLFYA
NSIISQIFMYCWFGNEVQTKSNKIPQAIFESGWTDFPLKTKKDLVFLLMKTREPIKVSAFNLFSLSLDTFMRI
LRTSWSYFALLNQVTZ
>TcasOR12
MQKFDWRSMIKMNIVVLRCVGLWPSGEESYKPGVYTIYASTVLTLFLLGHIFFQAVNVYFIRNNLSAVTG
TIYILLIEILLVFKVYYLVKNMTVLKQLLKMLETEMFQPRNSTQINEIQADMKFWQMLIRFLWVSVMCSN
LFWAIYPLVDNAGKEKRFPFLAWYPYDAQKSPYYEITYVYQTISVNYMSSIHVSVDALAGALNVYNGNQ
FDILCDNLRNLHRLTKNGTIDAGRNFRYCLKHHKHILDFAKKCNNYLNWILFMQFFVSTISIGITMFQLTV
VRPFSNEFYSLFTYISAIIGQIFMYCWYGNEVEVKSSKIFYATFESDWIEFSEEVKKELIFFVMRTQKPVKLS
ALNLFYLNLDSFMRILKTSWSYFALLHQISNRNSZ
>TcasOR14
MLLKWSSVIEFNLFLLKWIGLWPGEDYQLNMYSFYGFSVIILILCGHTLSTGLTLILDSGDIDTFTETMFILN
IEFMTAWKALNFALNRKKFMQLLDAIDKTTFQPRNGKQVTLVLRNIDGWKVMFKMFGISLGLSFIFTGLL

PIFSKTYKDRKLPMEAWYPFDSTKSPFYQLCYVYQMAAVAVAVMVILNVDTLVAAMNICIGLQCDLLCD
NLRNLHTNTSKSMNQKLIECIKHHQNIISFAEKFRQAFNWSIFLQFFVSTTSLGIVMFKITRFSLYVSEYYRF
ISYACSVLVQVFIYCWFGNEVIVKSSKIPYALFESDWTQDSLEMKKNMIVFILRTQKTLKITVCHVFDLSLP
TFLTILKTGWSYFAFMNRVTSPHZ
>TcasOR15
MLVKWSSVIKINIFLLKWVGLWPGEKYQLNVYSFYAFTVIILILCGQTLSTGLTLILGSGDVDTFTETLFVV
NIEFMTAWKALNFALNRKKFIQLLNAIDKPMFQPRNDKQVTLVLRNIDGWRVMFKMFAISLALSLIFTGL
LPIFTKTYKQRKFPYEAWYPFDSSKFPIYQLCYMYQMASASTLVVVILNVDTLVAAMNICIGLQCDLLCD
NLRNLHFDTSKSMNQKLIECIKHHKSIIRFAEKFRQAFNWSIFLQFFISSTSLAIVMFKISRTTNYGSEYYRFI
SFACSVLVQVFIYCWFGNEVIVKSEKIPYALFECDWTPEPLEVKRSMIIFIIRTQRILKITVSYMFDLSLPTFL
SILKTGWSYFAFMNQVTEVNTSKZ
>TcasOR17
MDDFNWISTVKTNLLLLHIGGIWPRGDGTHKLNLYTIYAIFITFTFTTYHCFSQIINFFFVDDLQALTESIFIS
LIQSMALVKAFYILKNMRILKNILKNLETNKMLQPRNLKQIKMVQPSLTQWRLLSQMFWISAVFAMCLFG
AFPIVESTYKEFRLPYLAWYPFDTKSSPFYEIMYLHQFVSSYTIAIVDIGADTLIAALNVFVATQCEILCDNI
RNINGSVEEMDSKWKECFTHHKEILKVARHCQKFFNWIVLMQFCASVICIGLTMFQLTLVVSFSSEFFSSL
FYFGAITVQIFMYCWFGNEVELKSSKILYATFEANWVEAPHQVKKNILIFAIRCQNPIKMSSLNVFYLTLET
FMAIFRTSWSYFAVLRQIQNRISEEZ
>TcasOR23
MANFNWTKIIETNFVVLKVIGLWPEKTFFACKLYNIYTHFMVTCLLVIHLLLQTIQLALIIDEFQLFLTALPL
LLQQYHLLIKLFYFMVKFPILRYILHSLNNHQVFQPQNQDQKIQMEDRLSFMKKIYFSFYSMAGVAISFLV
AFPILDILNGGERQILFVCWFPYDYMTSPFYEFTYFYQSASIIYAGVIVLQIDTLVTLLMTYLGFQCDLLCE
NLTQVGYNNSKENNTELEFVKCIKHHQELIKLKNHCVDFSSGLIFVQVATSSIAIGLTLFQMTLNVSTFNVI
FLVLYGLSVTFQMFQYCWFGSEVIHKSDKIAYSAFEMNFVDAPLSVKENLVIFMACTQKPIKMPVLKVTH
LSLQTFTKVLRTAWSYFALLVQVSKZ
>TcasOR27
MLETKQIVFHSFKLNVTVLSLIGLYPPKNYSILYKIYAVILFLAVHTPQLVLGLLHYFLMGDFTSIDYSDFV
TVGMMFYAFKLLPFVTSVTKIQKCINYFDTLGYKILKSEEKIIEDCVGSCRRNTNVFFVGCCLSWMGFVA
QVFLRDEPQQLPLKVWFPYSRDESPVLFYCIYILLIFGPGYSVLACGTIDPMIGGLAYHAAAQLQRLKRNL
QYLDEYIKEKNVGKSKENKRGVIYEEIISCVQRYQEIATFVDLFKDSFSQVVFSQFMGSVFLIGLCCFQIITA
TEVDINFVITANYIWVILFQIFFYCYYGTMLIEENYTLTNAIYLSNWYEYSIPEQKALFMLMERSKKPMIVT
AGKILDLSLDTFTMILRRSYSLLCCLKZ
>TcasOR32
MCLSTSEQSFSINLKIMKLCRLFPPTEGKKFYKIQAYLLQFLLLLPIPILGNLHLLLDENLDMEKVNYNAVF
LAQVTCFVIKLMAIIANSEKIKKCITELDSPKFAAVRENHKIILQHCIKVCKRNTLIFVVFVICGASSWATKP
LFWSRRNLPLDVWFPLDTTSTPVYCSLYIYLLIGVYFTSFANMVIDPLIAGLAYHATSQIKILKDNLQHLNN
VYANEEITSSKNKIIYMKIKRCVQHYDDILSFVKEFEECFSLAIFSQISASVFVICFSCLQLSKIKTFGYYFIQL
VFYFGVILAQIYFYCFYGSTLFEESSSIINAVYSSKWYDFDVPCRKALLILMERAQTPITVAAGKIMDLSLV
TFATILRRSYSLVAVLNNYQZ
>TcasOR36
MKGLVEKSFRVNLLVMQVMGFYPPQKYKSLYKIYTYVVYCAFTTLIPVLATLELFLAENINLEQISDNAFI
VCEAGCFIIKYLPFVRNADKIKKSLFLIERPMFHIYTKRQEHIIEECVAICRRNCRLFLTFCTITVINWSITPFF
LPGNNLPVEIWSPFEHKASRKFYFLSFVYIVAGVGNAAVSSGVIDPLLAGLISHATSQLKVLKNNLQFLDE
HAEERIASRNISFIERKRFKADFIYQQIKLCVNHHIAITEFIDVYEDTYSSSVFIQFAASVVVICISCLRLSMVE

PFTFTFFVMALFLWTMLCEIFLYCYYGTILYEENHSLTNAIYMGKWYNYDIKSMKALVILMERSKRPMIV
TAGKILDLSLETFTTILRRAYSLLLVLKNYESTPTEZ
>TcasOR39
MSNQHEIDLTEFVKLNIKNMHFFGYFFPRFGHNKTRKTLYTMYSTLFVGSTFVLTALSQIANMINSFGDM
ERMTEASFILFTNVVQCFKIYSFLTYGPRVWNLIDGLNRNIFKPINTDQHRILVNDIYMSKKISKIFLLACTL
TCMSWAISPFFDKRGDVLRLPLSGWYPFNTDKSPAFELVYIYQILTTWIGGMGNISMDTFISGIIMAISSQLS
ILNNALKNITKNNELVRCVFHYRIIIKFSDEVIYLFNTCLTTQFIVGVIIVCISMFQMSLVPVLSFQFVAMLLY
QMCILLEIFLWCFYGNEVMLKSDQLTQAAYMSDWTKSPNHFKQNLLFFMTRTQFPLKLYASGYFTLSLE
TFKAIVKSSWSYFAVLNQVHSRQTQZ
>TcasOR40
MSSLIQESLHINLRVLEFFLLYTPGEPTNFQKLRSSILFFALMFHVPVLSGINLIVGKHDNPMKLVDNSFGFV
GLSCYIAKLWPLIGNRSKIKVCINYLDKPIVELRENQKGILQACSKICRRNSNIFLYYMIISVTGFVTKPFLFE
ERGFPVDVWLPTSLKDRLDVYWGFYIYVSIGVAYPVIASGVLDPLIPSLLCLATGHLKVLNDNLEHLDEYS
SEENGSKDSNLYKNIQKCIKHHIEILNFVYNHQKCFSLMVFSQFLGSPMILCFTCWNVSMREPFSLEWFQS
LAYFLGLLLQLFFYCYYGTRLSEEFEHVTTAVYMGKWYKYDVKSRKALIILMERSKKPTIVTAGKILDLS
LETFTIILKRSYSLLAVLKNQNZ
>TcasOR41
MDNTLDIDLTEFVRFNVNSIHFFGYFLPEFGKHPKKKIIYVIYAVIFVGTTFGLSLVSEIANMINAFGDIEKM
TDASFLLLTNLVQCFKMYSFLTHGPRVWKLIHSMNNSDFKPKNLEQRNILVEEIKMSKRISKTFFMACTIV
CSLWGISPFIDRGNSEKLRLPLSGWYPYSTDTSPGYEITYAHQTLTTWIDGLADVGMDTFLSGVIMVIAAQ
LSLLNNSLKNLTKNCKNDGKKANTNLIECVIHYRTIISFADEVTYLFTSCITAQFIIGVIIVCVSLFQMTLVSL
RSFQFFSMFLYQGCVLMEIFLWCYYGNEIILKSDELTRSAYMCEWIEESREFKKNLIFFMTRTQFPLKLYAS
RYFTLSLETFTAVVKSSWSYFAVLNQVHTKZ
>TcasOR46
MSKSEKIHTLATYFDSNIAFLKLTAFWIYDDETTRRKKYLQHAYNIFWIFYLFVAYQPAELLYVYYSFNDL
SVFLRALRDIGNHVSLAYKAFNYFIMRRDILKLMETLQHGNYHYEDCGDFQPKLIVDEEKKEALKWTKY
FLNFCNAICLSMFANGVFTFIFLSDKQYVERNGQRVYHQEQPVNTVSPFGSGTKLRFFVTFIYTMIALTFY
AWTIVALDSLFITIMSCISSHLKILQGAFKTVRARFIKLCASLSKLLISVSGKLESIYSTQTFVQTFISLGEMCF
SLYLLSETADQNIGNEITYLIATGFELLMYCWFGNRITEASLKISYALYESDWFPTSLSFKKQIIFTMTRMQ
KPINVTIGKITPLAFSTFLTIARGAYSFFTFLKQRHGINHZ
>TcasOR48
MPHATLSKMVVQKIDLLEPFDNVTRLLKILGLWYSPNETIVYKIYKNFVMATCFLYTLTCTVYGFKFMSF
ETLEIAFGAVEGVLKSLMFRLKFQKIAESWQQIRQQEFQPRNEHQRTVLKWYIEVTKSLFLVYFFGVYIGC
ISALTVSSWLRHKDFPTDHWFPFNYRRPFLYQYIYVHITVGFYLTAFLNCASDSCFYLSLLHITAQCEILAD
TLKNVHDLHKLNAAKKNSGQKGEDEVMNQILIECMKHYNLIKKYTSLVADCFKEIITLQFVPTIVMICIA
MYKISTLEPSNTQFWFFAFTELGAITQIFIYCFVGNLVTSTSQKLFYATFESQWYNASQKFKKNLITVMMA
VQRPVIFYGWNIFAINYATFKSIVQTSWSMCVAFRSTQDLZ
>TcasOR49
MVVEKINLREPFENVTRLLKILGCWYFPNESLVYKMYKNFALITCCMYTVTSIIYSFKYMSIDYDKAYESL
EIGVGTAEGVLKGIIFRMKFQKITESWQQIQQPEFQPRNEKQKMLLRRYIYVTKFLFKVYFFVVYIVCVTG
LIVSSLLRHKDLPTDHWLPFDYRKPFLHQYIYLHLTAGLYLNSLTNCAVDSCFYLSLLHITAQCDVLADTL
KNIHDLDKLNAKNAPERENKDQVMNKILVECMKHFNLIKKFTNQITDCFKEILTLQFVPTVAMICMGMY
KISTLQASSSQFWFFVCTDLGATTQIFIYCFVGNLVTTTSEKLFYATFKSQWYNASQKFKKNLLTFMMAV
QHPIIFYGWDVFAINYETFKSIMRTSWSICVALKSTQDLZ

>TcasOR50
MVVEKINLLEPFENVTRLLKILGCWYSPNETAVYKIYKNFIIATCFIYTVTCNIYVFQKMFTDSDKAYETLE
IAVGSAEGVLKGIIFRTKFQKITESWQQIQQPEFQPRNEKQKSVLRRYIEVTKTFFKVYFSLVYVGCVTGIV
VSSWLRHKDLPTDHWLPFDFRRPFLYPYVYVHVTVGLYLNSFTNCVLDSCFYLSLLHITAQCDVLADTLK
NIHDLDKLNGKNVPERENVDEVMNKILTECMKHFKLIQKFTNVITDSFKEILTLQFVPTIAMICISMYKIST
LHPSNTQFWFFIFTDIGATTQIFIYCFVGNLVTTTSEKLFYAAFESQWYNASQKFKKNVITVMMAVQQPIIF
YGWNVFAINYETFKSIMRTSWSICVALKSTQDLZ
>TcasOR52
MSQIDLKEAFKQNIVLLKAMGLWFFQNERFYKLFKCFVQGSLVFDSTSLIIYVALNIRIKNVTDTIYSLPGS
LEVVLQAILFRKNFHLIRKSLNNLKQKEFQPKNDTQEKILKDSIALSRRVFYSFFWLVFVMIGMWMVLPLT
KKGKYLPTKYWIPFDYRLPVVYELLYVFECSCIIFHAFSNVALDTFFSIAMIQIGAQCDVLCDTIRNMDEQE
KTNTMDRILIECVHHYRLIEDFAKSIATSFKEILMVQFVCSSLMLCVSMYELSLSEPMSGHFFQVLLFQISA
TNEIFLYCWFGNEVIIKSERLFYAMFESKWYDSAATHRKNLMIFAHQVQKPISLLVWNIFPVDLKTFGGLL
QKCWSFFVAMKNIQEIQEZ
>TcasOR54
MNLQKLDPLEGFKPTISMLKIFSVWNSSNMFYKIYKNVTTLSLAITYTCVMICVVVNFNVSEINENFYYIP
ALSTAPFKLVIFQKSFKKIQNLLFLLQSQYTKIRSEKQAKMVEDSVVLSKRVVKVFAVLVVPTCVGLFGM
PLLKDEIKLPLIIWIPFDYHEPVVFGLVYFVISFSGSFTAYINIGTDTFFYNCLIQIETQCNILSDTLRNLHEFG
RFEAEIHTILIECIEQYKTILKFTKILSKTYQGILSVQFICSLLSLCLTMYRMSLADPGSEEFLRYFVFQWGVL
PEIFLYCYFGHRVLDSTKNLYYSTYELQWYNTSAKFKTNLLIFMGQIQNPIVIYVAGIFSLDLETFKKIMQK
AWSFFTALRNIHEQZ
>TcasOR55
MKAFSFNIKLFQIFGQWCYENESFYKIYKYTATVLLFLDWLFTMIFVLVNFQESEVVDSLYISPSMTTSML
KYVIFRVNFSQVERMLKIVEEQYLKIDSRRVGFLVERGTKSSVFVIKACFYLVMATVVSLITQPLLQEDIDI
PLVIWLPFDYHRSGIFELIYVYVSVSYLYFAYVNVATDCFFYISAIQIGVQCEIVGFMLENLNEIAKQEEEN
VRRLFLSCVTYYNNILECVKIISDCYREILIVQFFCSFVALCMTMYQLSIVEPFSDVFFKMCVFQSAVICEIF
LYCFFGDLVLEKSGKLFYASFSFGWYNGSAKFQKELLIFMNQLQKPIIFHVGNVIPVTCETFKSIMQKSWS
FFIALKNTQNRZ
>TcasOR57
MSHSNPLEAFKLNTFFLKALTVWHVENPTYRLYKIFVVFSFAVTFFSAWICALVNYNVSEISENFYYLPAM
STGPLKYAIFQKNFTNIVNLTHLLETQYAKIRTENQKKIFDESVIFERKVMKNFAILIIPTCVAMFIVPYFQD
RREMPLIVWFPFDYKQPVVFDLVYFILAFACISIAYTNVSTDAFFYTCLIQIETQCEIVSDTLRNLDKIVTNG
FRNVAESRKIFIECIEQYNVILRYTKIVSDTYQGILVVQFFCSLVALCLTMYKLSLADPGSQDFIKYFVFKLG
VISEIFMYCYFGHRVLEKTEDLYFAIYEMHWYDASKQIQNEVFIFMGQLEKPIVFYVANIFSLDLDTFKKI
MQKAWSFFTALKNMHDIRNNZ
>TcasOR65
MTATKSLKEIPPIYLRVHLTVLQILGIDILPVESVPQNLFYTYTALIISTMCLFTIAEFLDMVLNYEDIYRLTF
GLCYCVTHVLGTVKMFLMLYLRKKLWGNLTTLEEGIFKPNPTRGGPEELQIVNDAITMCNRQGYVFYTL
VFLIIGARLLYASLANWPYDKHNYFDGNVTVIVNTKEMPYTTWMPFDYNDSPLYETIFAFQIFSTTVYGF
YIGAADAVICGFMMLIKAQFLIVKRELETLIERAQKAAIAENPDNEDNFGREIERIELLDKRTQDYVAKYA
NECVYHHQELIALCDHAEEDFCYLMLLQFISSLLIVCFQLFQVSTLSPDSVEFFSMVCYLLLMLFQLLCYC
WHGNEVQIVSGELSRYAFGINWIIMRESPKKTLLLLMMRAQRPCYFTAGKFSLLSLQTFMTIVRGAGSYF
MFLRQMNIZ
>TcasOR70

MPSIIDISFKININVLCLAGLYLPDKFKSLYRVYTYLVYVFIVIPVPTLGCVYLLAQEKITFRQIADNLFLIAE
LGCFIPKYWPLVRHAERIKRCIHYFSAPIFKTDRKEHQEILDDCIKVCHQWSAFYFASVTAGFVSWSIRPIS
WENHILPTDIWLPFDPHTASSAKVASVYFYLVLGKGFGLGIKILKNNLQHLGEYVDEELASLEPCRKAQLT
YQKIRQCVIHHEHILAFVEEYEECFSQVALSQFVGAVVIFCVSCLQLTIVEVVSLDFLAMMMYFIAMLCQV
YLYCHFGTILYDESDTISDAIYLSKWYEFDKRSKKALCILMERLKRPMTVTCGKIFTMSLVTFTMILRRAY
SLLAVLENYNIELNZ
>TcasOR76
MMESTVTRLKRMYLWPTASVTSRKPAFFLITFSCFLLYGSVMHLIVNDISMEEVHVIETTAGQFGVLYYL
TLFTIYRKGILEIYADLSNFTKFGKPYNFDKRNKQLNQWSRWFSVVLYFFVISVFAWPGIFTQSCEDLNVA
LNKTEVCGVVSPVWLPFRFDYKPMKQFVYFWQSFCCLYSNGGAGTISFAMSETIEHLILRVEDLKILFPKI
VAERSPEVRRKMLAKWVDYHLWLLSIGKLMNDTYRYSFSVIVLCAGTLFGCIGYTVMKNASTNFNSSFIF
FGWMESVFVICVCGQRLMDAFHSVGTTVYNSEWCDTDVDFQKGVILITIRAQKPVRIYAGPFSYVSHLLI
LTVFQTSYSYINLLNASSZ
>TcasOR77
MKYILMKKTIAFLSVTGFWPKTKESTKTRAFCILFSSSFLLFGSLGYLIVYRKFGSDDIDSIETATSHFGVLY
FMFFWILKRDGLVHIVNLLSDFSKFGEPRFFNDRNRQLDYLLQYCIFVLSVATGGVFLCPIIFVKNCEMVK
QEKNLTKVCGLVSNVWAPFDYSEYPMKRVVSLWESYCCFINFGCGGIMSFTMIKTMEHLHIRVEQLKDM
FPDVVNEKNLAVRKQKLEKWVKYHLHLYDIGELMNNTYRYCLSVIVLCVGILFGCIGISTMQPGSSHNSL
FLFMGWFQSICILCMVGQRLLDVFLSVGVMAYDSAWYEKDVDFQKAVLMIMIRARRPVLIYAGPFTNLS
HLLILGVLQTSYSYINLLNAKZ
>TcasOR78
MGHAIMTEILTYLTLMGFWPRSPKSSKASAFLIILSTSFLFFGILFYLIVNRQFGSSEIDSIETITSQFGVLYYL
ILFTWKRNDIVEIVELLSDFSKFGKPPFFDQRSTRLNYRLSCIVLILIVANIVVAALPVIYIDSCHKANEQLNL
TKTCGLIAPVWLPFDYNEYPRKHLVFAWEVYCCVMNYVGSGIGALTMVGTMEHVIIRIEQLKYIFPKILD
QPNPRIREQMLKNWVRYHLALFEIGRLMNDAYKWSLSVIVLCVGALFACIGISMLQSTASQINSICLFFGW
FPSIAFLCMWGQRLLDSSLSVGTAVYSSRWYDMDVAFQKSVLMILIRSQKPIRISVGPFTHLSMLLLLGVF
QSAYSYINLLNATSZ
>TcasOR79
MGHVIMNEILTYVTLLGLWPRSRKSTKTISYLIILSSSFLFFGSLLYLVVHRKFGSNEIDSIETVTSQFAVLY
YMTFFTLKREGTVRIIDQMSDFSKFGKPPLFDQHNKRLNYLLSYFVICLFVAIVGVVALPAIYTGSCHKAN
EQLNLTKTCGLVAPVWLPFDYNGYPLKFLVFAWEGYCCIITYACSGISSLVLVGTMEHLIIRIEQLKLMFPE
ILNEANRHIREQKLKNWVQYHLALFGIGKLMTATYTYCLSVIVLCVGILFGCIGVSTMQSASSNNSVFLFL
GWFQSLIVLSVCGQRLIDTCLSVGIAVYNSRWYDMDVSFQKSVHMILIRSQKPILIYTGPFSYLSHLLILSV
LQTAYSYINLLSARGZ
>TcasOR84
MTEEKELRLCLWSCYYLKLSLMWPLKREEFKSSKGLYLRLLVFVIISGSTFTAMIFMHLYKSLKVGSYDV
SEDLAILASNIGYVLMMTMYVSRQKDLELLLLDLSDFKTYGKPPNFDKVRKRMDLYAHLIFFYSMFGSFV
YNMDKIILIDKCKEARRINEVCGSAIPFWTPFETEDLFTLTLVITYVLINIFVVVKVAMTVSVQVLEISSHIN
LRIEQLKIFIAGCFDRDFKASRERLDFCIRYHNVIIDFSERFSRCFSYVMFIHLAITGIIIGCLENQIVQEHQPE
AMLHMGGWSTATFIACYGGQLLMDASTSIADEFYNCPWYEADVKMRKDLILIILRAQKALFVSTGPFNV
LSFALFVSIMKLSYSIFTVLSZ
>TcasOR87
MKHVIMDELLIFLTFLGLWPRTPTSPKIISYLMIYSTSFLFFGSSIYLILHRKFGSDEIDTIEIITSQFGVLYYLT
LLVVKRDGITKIVNLLSDFSKFGKPPLFDQRSRRLNLLLRLFVTVLLAATVAIVSVPVVFINSCNKQNLQLN

ATKICGLAAPVWLPFDYTQNPRKYFVSAMEIYCATMNYAGSGSGAFLVIGTMEHLVIRIEHLKNMFPEIL
NEPDKQIREKRLKKWIEYHLSIFEIGELMNETYKWPLSVIVLCVGILFGCIGVSTMQSVSFQNSSVFLFFGW
FQSIFVLCFWGQRLLDSCLSIRKAVYNSKWHEMDVSFQKSVLMILIRSERPVLIHAGPFSYLSNLLVLGVL
QTAYSYINLLNARSZ
>TcasOR89
MKEAVLQQSKKEMHLLNLWPKGHVKHFRFRYVITLIIVSPFTLGTLTHFINVLKENLDVDLSGDISVIAVV
TGLHFMLITFVWGHKKIAYLWENLGPHEYFGKPDNFEKRCKQLNFYSRLYAYYCYLGLTVYIIMKNRGG
IECRRLNVERNLTEICGLVTTFWAPFDIDFFPFRQILFVDQVFATYFIVKGGAAISFTTLEVGEYIILKIKHLK
RLVKEVFDDPREEVQRKKLVFCIKYHQYIISIQELYDGRYKHCNGCYILMVGIIIASLSNEIMKNHNIEALL
HLVGWVFSFYICCFSGQSLLSESLTIPDAAFESKWYEAPVYMQKDLLLMMLRSQKPLMLHATPIGVMSLS
LFITLVKTSYSYFTLLNQSTZ
>TcasOR90
MAKDTSPVLRESIEVMKYLQLWPQNERTNLRRRYFIVIFLCSPLHLGLATHLVVCLKDNLDVDLSANIAV
LSAVTGLTYMLIVFVWSQDKLVHLLAKLDTHEIFGTPDNLTKRSRRLNFYAKLYSYYCYFGIVIYSLVQII
EMPQCRKMNEEKGLSEICGMIVPFWAPFDIDWFPLKQIFWLNQLLGIYIIIKGGAAVSITTFEVAQYICLKIK
HLNRLLREAFDDPCDVVVEQKLLHCIRYQQHIIRTNELFNVCFKHCNGCYVVMVGIIIASLLNQILKEKSV
GALVHFAGWICSFFICCHAGQAVISESLTIPEAALDSHWYEAPVKYKKVLLLLLVRSQKAFNLQATPIGIM
SFDLFIALLKTSYSYFTLLHKSTZ
>TcasOR92
MKNQEIKICRATLTVLKYSLIWPSEADEMNPGKWYYIRVVTFILFTCPWVLSVFMHLIVSIRNNADIHLSE
DVALMVAFTGVYYMTIIYVKKQPKVAFLLRDLSYFQFGKPPGFDETERILGFLSKLTFCYSVMAVVIYNYI
KYRQKPECERMNKLKGLKENCGMLTPTWWPFEINYSPAFQLIFLYIFTSTQVMMKLSLMISFNVLEMAH
HIILRINHLKTMILESLDEQDYEASKRKIKTCILYHLEILGFAERMDDCFSNGMFAHLTITAAICGCLEKQFV
DGDNQLGSLLHIFGWILALFLACLGGQHLINASETISDAIWSSKWYDADLRLRKDLIFMMARSQVGLYLN
VGGFGILSYALFLSVIKMSYSILAMLTSZ
>TcasOR94
MAIKICKFTRKNMQISLIWPREFEEINPGKWYYIRIVIFLITYGVFPFCTFLHAVVVIHNNLDIRISEDIGAVV
SNIGISYMAIIYVQQQNQIAYLLKDLSDFKDFGKPPFFEEENKRLNFWSICTFIYPTCGASLYNLSKILEKSE
CNKINEENGLPATCGFIFPIWVPFNINYFPLFHIMLISTWFCTTMFVRLHLSISYNAFEIAHHIILRIKHLNGMI
ITCFDCQDYKISRQKFTTCVLYYKQILDLSNRLNQSFSSIMFVHFTMTSAVCGCLEKQFVDGEYVGGFIHL
VGWIISLFIASVGGQDLVNASQSISEAIWSSKWYLADIRLKKDVLFMLMRSQKDLHMSVGSFGVLSYAFF
VSVLKMSYSILAMLTSZ
>TcasOR97
MNNQKIQISNMTRKVLRYSLLWPKTNEELNPGIEYQFSVLGFFLVTGVLVLCITIRFFITIKAVHEVDAEVL
AILIASYGSYYMICAHLKNQHKVALLMRDLSVFNNFGKPPNFDKRNNQLNFVAKLLALYSFLATIFYNGE
QLINKTECKRINKEKGLSDHYCGLLAPCWLPFEIDYFPVFHLILIYAFTSGYLLIKMAIHISYNAFEIVSNIVL
RIEHLKAMILETFENRNKQVCHKKFLQCILYHIEILDFAARLDDSFFNSMFGHLALTGGICACLEKQIVSGV
NVVAGTLHFIGWILALFIGCVAGQYLINASEILPSAIWTAKWYDADLELKKKVLFMLARSQKSLFIRAGPF
GILCYPLFVTVLKTSYSILCMLTSZ
>TcasOR98
MVKKESEIKISRVTRKLLQYSLLWPTEGEELNPGKWFYFRIFAFLSFTSLWCIAICMHFIFVMKDKPDWDP
TEEIAIIIAIYGTYYIVLAYVKNQRKAAGILRDLSNFDKFGVPPGFEEEEQRLRVYIICVFIYGFITITFYNFYK
MSQKKSCERFNIEHNLHENCGLLSPVWIPFRIDKFPRYELVFLYLLTCCHLLMKLPLIVSYNALEMVHHIIL
RINHLKIMITECFDDPDYEISRRKLTQCILYHTEILEFATRVDDCFSNCMFAHLTLTGTICACLEKQIVAGFS

RFGAILHFFGWILALFIACLGGQQFINASDTIPEALWASKWYNADLRLRGDLLLMMMRSQRDLHITAGPF
GVVSYALFVSVLKASYSILCVLTSZ

>TcasOR105

MKPALKLANVLGLDPLRNDNYTQLKKMFCALCIVSLFVSAYLEFFSNFTTFETYETAPESLIPHFQTMFKM
YSLIFSRTEIVELIQMAEQFYKFSQCDERKKLTKLYKRVDLFFYVYASLVAAACVLFAIVTLIFKPGKPIFLC
YGGLHGLESPEFEIYLVVDLIGIVIISVTVPAFDGLFFYFALYIYTEFKLLKIAFKTMSGQELREAVKHHDFL
LKYIKKLNSVYSPIFLYQFFCNLLAICFCLFMLSRSGIPPEMVSFSKYFLCLLAFLVQSYTFCSIGDLITELSE
DVSNAIFYTDWLDDEAYENKTARLIIMSRAQNPVMLTIGKFANMNLRTFILIVRNAYSFLAFVNHALNZ

>TcasOR106

MESALKLIDIIGLHPLKSDKYSTMRKTISFLSLVVILISAQLEFLSHLSVFEVYNSGPHSTIPPLQSLLKMATL
HFYKNELIDLMEKSKSFWKLDKFGDLYKQELSKLHRLVTIIVYIYIALLTATCVQLAVLTLIFRRGKPIFLC
YGGLYGLESPHYEIYSILDAIGIGVISIAVSGYDAMFFFFALDIYTEFKMIKSAFKRHSDQTVSSYNKQFIEA
VKHHDFLLQYINQVNDIFSPMFLFQFFSGLLGICFSLFMISRSGLQDINTLSIYSAGLLGFTAQSYTFCLVGE
VISELSEDISNEIFYTDWLDDEVYRNKTAILIVMNRAQESPKLTIGKFADMNLRTFIMIVRNAYSFLAFINN
ALDZ

>TcasOR107

MENPLKLLHIIGLDPRQSDKYSTIKKVISFLIVLAVLLSALIEFFLHHNESQVYDTAPQSTVPNLQALLKMF
ALIIYKKELIDLFTKGNHFWKLDKFGDCHKQKLTKLHKYVDLFFYVYAVIITGAFLQLALLILIFEPGKPIFL
CYGGLYGLESPQFEFYAVLDFLAIGVIAISVTAYDSIFFYFALYIYTEFKMIKIAFKRENCAQFIEAVKHHDF
LLQYISKVNEVFSVIFLTQFFSGLLGICFNLFMISTQGTRDMKSFSTYFVGLVGYTAQSFTFCLIGELISELSE
DISNEIFYTDWLDDEVYRNTTARLIVMNRAQESPKLTIGKFADMNLRTFIIILRNAYSFLAFINEVLDZ

>TcasOR109

MGKVKFTEPLEFLNVVGLNPENCSNFSLFRRVISLGFFLVVITLGLLELLLHFEGLETCSRASEAMIVQYQL
FIKIAVLLKHRKNLVVLMQKTRKFWPLDKFGQDAKIERPHKLLKAFFFAYKLIMILMALQYILRKFVSKN
GKPLAIAFGESKGLSPKVDHLYFVLHSTSTFVVLHAVTGFDRLFFFLIGHVLTELKLVKKSYRLTQNRREK
FLETVQHHAFALEFVRKLNRIYSQVLLNQHLSCLFGICFGLFLVSKDGIPPDLGHVTKYVPYVISFITQTFTF
CFIGSLLITWSLQVPDAIFYNDWGKNQAYKYKTDKIIAMIRGQRAAKLTLGGFGDLDLESFNLVVKNAFS
FFTFVNAMNQKZ

>TcasOR110

MDKVEFSDPLFFLNVIGMHPFKADKFSKFRLAFSIAVYFAVIFSGVLELIVNSQGLETYARASDTLIPQCQL
VCKIFVLAKYKKQIARLLNGSQRFWDLGQFGARYGNSFGKTHKYLKSFFLLYKVMLTFTCLQFLAVKIIF
KIPKPIAISFGETKGLEPLYDHLYLVLHAMITLVTINLVNGFDGLFFYFIGHVLTELKMVKVAFGDSPIETN
WSEEKRFKFAVRHHRFVLDFIEQFNIVYCTMLLVQHLTCLFGICFGVFLMTKDGVPPDLDRASKYLPYIVT
FIFQTFTFCFAGNLLLSWSLEIPNEIFYHDWAKKTTYENKLAKIISMKRGQRAARLTLGGFANLDLDSFRM
VLKNALSFFTFVNAMMNKKAVTSVZ

>TcasOR112

MITRLMAQFAIKGRVGTGGYIMDKVKLAQPLAHLNIIGLDPLKNDRFSKIRTVITVAVFALCNVFSFSELFL
HYNNPHVIVRSSEVVFPFFQNDWKIAIMLVYKKNLAQLIQNTSRFWQIDAFGKNYQYSMGIKHKYVRIFY
LVYRLMLMFSCSQYILLTIGSDRPMILSFGETGGLGSGALLFYLIFHIVYLLIIFNVINGFDGLFFFLVAHVLS
ELQMVKVAFSSSKVITFWNHKRRFKSAIQHHRFVLDYINRLNSIYSILLLNQHISCLFGICFGLYLFISDGFP
PDYEHISKYVPYVIYYITQVWVFCFAGQLIIDWSVNISDEIFYHDWTLNRTYENKTDKLIIIQRAQHAARLS
LAGYGNLDLQSFNLVLKNGLSFFTFVNAVIHKZ

>TcasOR113

MLDAWERLTFYPFKWISLGGLHPQNDRLVAKFLFLYNFAGFGTILGLAITQIYLSYENIYYTIDSILTIVLYL

HIASKYVNLHLHKDTLAMLIQERSKFWPIDTFEMTVRQKCVRILTKSLTIIKSYLGYSLLVVISFLIQPIITGQ
LPVFMYVPRGTYYIFFVIFMTITPGIMSSIWGVDTLFFSITTPVSIQFKLLAHKFETIDLKMDSKRVRHEFRK
LVDYHNFLINYCQNINRMSSGIFLTQYLVAIATSCMQLFITSQPEFGLLNKIKCLTYFIMQIIETGIYCFTAQL
ISESSENVGNAVYKAPWYDFNCGTRRDIALVIVRSQKKVVFNGLGLVWIKMETFTKIFKTALSFYTYLNT
MVYQNZ
>TcasOR116
MPETLSFYPELMLKSAGLHPYTKLKIVKFFYHHYLNLFFFIFLLFLAILEVGVSVKCDIYRAIEALSSVLFMT
LTLFRYVVNYRNKPSLAWLLEKRSNFWLLEHFEGQIRTDCAKIMHTSSNFIRNYKNYAIVLAAVFYVQPFI
FHELQMKIYVPEGWFYYLYLVYWYMTPPLFVSVYGVTSMFCAICIPVTIQFKLLAHRIQNLDFKSEKFQR
DLKHLVDYHNFLIDYCTRINRYSNGVLLFEFFITISVCCILIFIAANDYPFVDKIKYAGFIVSQFLDTAIFCYN
CELISDASENVGKAAYDSLWYESESKIRRSLILIIVRAQKKVTFSGYGLVRINMMTFTQVFKATLSFTSYLN
TVTVDEKMLNNZ
>TcasOR117
MDKNLDPDDLSAYPLKFLWYGRLHPGLNPWWTKILVPVNVSVAFLYLVLAIKGIFSSYNHDTFFTAECV
QTCILVVHAIGKFSNFLVHKNSLLRLVAKKSQFWKLESFDGDLYNECVWISTFVKKITRFYYFLNLFVLISF
DLQPFTTGYLPTGCYVPEGWFNFLTGLLWYLSCAVLFGLPGTDGFFCSLATSLIIQFKLLGYKFKNTKLYK
NEPDITLWNNLKQLVDYHNYLLSYSKELDATFKTIFLLQFMISIGSASVSVFIFMQPGDWSNRIKFLLYFVA
TMVQTAFYCIPLEFVVSSAKQIGDFVYESNWYQVKDIKFEKCFTLILARTQKNVVFSAYGLIWINLGTFLV
ICKTVFSFYTYLNSVNKITSZ
>TcasOR120
MAKKFLLDISADSLRLLWLGQMHPLSPFRRFVTFLILNLAACWLMIALAIKGITISYKSDIFFVAECLQTCN
LMFHGVGKFLNLYFQKNNLKSLLENRSKFWQLDDFRSEKLYSQMQGITFVIKKVLRYYYLLVLCVVFLF
DLQPFATGLLPTGCYLPEGWFKGLTLTLWFLSVSFFLNIQGTNGFFYSQSVSLIVQFKLLSHRFKTTQFDK
KELKELVDYHNFLTSYCKQLNQAFAAIFLLQFFTSITSASLSIFIFMQPGAWTNRIKFILYYSYTLVETSFYCI
PAEILVNAASEIGNSVYDLDWHKIRINRVKKCIVIILARTQKTMVFTGYGLVNMNLQTFVVYVKTVFSFYT
YLNSVRKIZ
>TcasOR123
MAKKFRSDDISADPLRLLWLGQMHPFSPFRRSVAFLVMNVSACWLLAALAIKGIITSYKNDIFFVAECLQT
CNLMFHGIGKFVNLFLHRDNLKKLLKNRSKFWKIDDFQSEEIYQELSEITSTVKKGLRYYYCGVVVVMLL
FDLQPFATGSLPSGCYVPEGWFKSLTVMTWLLSLSFLNGVQGMDGFFCSISISIVIQFKMLTHRFKNMRLF
HNESERKMWKELKELVDYHNFLTRYCKLLNTIFASIFLLQFLVSIISASVSIFIFMQPGAWSNRIKFILYYLA
VVAETSFYCVPAEIIVNSASEIGYAVSELDWYKIRINQIKKCFVIILARTQRTMVFTGYGLVNMNLQTFVV
YVKTVFSFYTYINSVRKIEKZ
>TcasOR126
MYNNKDSLLYDPLRFFGFIGFHPIFNSLILKISFYSTTTLGLFIYTMAIIGIVKPEETNSFFTLECLQTCILLSHT
IGKQVNYYMNSNKIVKFLQMTTEFWEFETFQGTIHPESNFLFHTVRKMIRYYFLVTTFGFIFFLINPPVCYV
PEGWELFLRIVRALTFWSYYTSTMATDAFFVACGTLLLIQFKLLGHKFKNLDTQSQEKWNNLRQNVKHQ
IFLHSCKLLNQIFAVVFLIQFLNSIAALAISIFIFSKPGSWNNRFKTLFYLVVVLFENAFYCVPAELVSAEALK
ICDQLFASKWYESNVAQFRKSLVIVLCCTQKVIKFSSFGLVEMNLQTFVLISKTALSFYAFLNQLKRZ
>TcasOR127
MSPRTLEMIADLPKDIITDSLKMLRLGRHHPTGSIWWTIFFIPVNTFFCSLLIILSIVGIVRYHEDDVFLAVDC
LGTCTLMLHAISKQIFLHAQKNAINVLLKMKSQFWNLDDFDGEISKECEMILTSGKIAVRIYFSFTCAAAT
FYFLQPFTAHHLPSDCYVPEGWFPFLAISYMYLIPTLVPSVVGLDALFWALGLSLAVQFKLLAQKFKLLGT
CHENETAILWNQLKELINYHRFLIDFCKKLNKLFSFIFFVQSFITITSASVAVFIVMQPGNLSTRVKCLLTFV

SYILEMAFYCLPAEMSVNAAIDVADSVYNSKWFRIKSTEFKKCLILIIGRAQIPFTFSGFGLIHINIRMFQLV
CKTTFTFYTYLNTVQNRQZ
>TcasOR133
MEYLKYCQTYIKGTGLASNSSLFRNILAKYFFLPPCFLIIAFSIHELWDTNNNDVSLVTEVLECVASYTQLII
RKYIVFTQSDLMVEIINDCGKLWPFDMFGSELGKKFKQQMKTCWTLVKFLVVCGFATFFLMCISARAAE
RDNSLPFLCWVPDFPYATELLFFLQFMLLLELLYYVLATDAFYILICMDIQIQFEMMGKMLKSIKFGEISEK
ECWDKLVELAKQHDRMLHQKLNQVYSKYYVVQYFMTVGSMTVQAYNLKYRMVNIQTALKSIVYTFSL
MFQCGYYFFPASNIEIEAENFSTEIYFLNWQNIGNIKIRKHILFMLMKSQENLAMMGEGMVHVNRNECLM
MFRLAFTIATLLDGLNQVZ
>TcasOR136
MEYLKYCQSYIIGSGLASNSPVFRQFLARYLFFPLSLILVSLSIYIIKDANNDIYFLTEVMESLASYTQLLIRK
YMIFTQSKLMVEIINDCENLWSLELFGPELGKKFKQQMKNCWTFVNVLVTSGFSTVLLICITTLTDKEKSL
PFVCWIPGFPHATELIFLTQFVLLMNGLYYIKLTDAFYLLVCMDIQIQFKMMGKMLKTIHFGLLSEKESWE
KLVELAKHHNKILHKKLNKVYSKYYVVQYVMSVTAMTAQAYTLKYIEVNIQLALKSIMYTCSLLLQGA
LYFFPASNIEIEAENFSTEIYFLNWQDHGDVKIRKHILFMLLKSQENLEMMGEGMMHINRNEYLMMFRLS
FTIATLLGGLNQLZ
>TcasOR138
MGASIHYFNYFYGHSQSSKFPTSSKSLKARIFNYVLYPISCTILAFMLYNFRYMNDNIFKIARNCQSITSFGQ
IFTRHFIYAQHSFLLENLLKNTAKNWKYDPKNPLFGHKTWTRVILVKSLKIMSFFVVTFIIILPFFCQDIDLP
QACWVPGSGHKMQLLIYFLQSVCLLEPMILLDMVDSVFLLTGVELEIQFILLRKAIKNVQISEQENQNFCM
GKLKLYASYHQFLLDQHTVLKQAFSSFFFLQYLVSVQGLCVEIFVIKQASTIEQIFFSATYIVANAMFLSFA
FLAASYLEIEAEALPHAIYSIDWYNGDEKLGKHVLFMLMRAQKPLILTGAGMFAVTRNALLQVYRLAFSI
STLLKQVZ
>TcasOR139
MSDHFRYVSTIFKLSGYLPSLKTLKTKILKYVFSPIMFTLITFIIYNFRYMHHDIFEIARTCEALSTHGHVFGR
KLAVLKHANLIEQVINDRNFFWSYEKFGEKLGQRFRQKLFFRDYVMKSLSAMSVLMLAYFYLTPVFVRS
VNLPQASWVPQFSHATALVYFCQVVCLSESIPVAVIIDGTFLLMGAELEIQFNLLKKTLKAVEIGQNSGQK
HEEKCLKQLKICASYHDFLLKEHVKMKTIFSEFFLLQYLLSIEGLCIELFVVNKAQTWGQFALGAIYVVGII
MQSSFTFLSASNLEIEAESLADSIYDVDWYNAKDPRIRKHILFMLMKAQEPLRLTGAGLFDVNRNMLLQI
MRTGFSISTIMRQFZ
>TcasOR144
MAFNESQDNFKLCFIAFNLSGLGPSSKPFLKILSYVLYPWLCLLFVLVCVNIVFKHSNIWDIGEVTSSISIAV
MMVVRKTILIKYSSVFAEIIELHSRFWDYGLFGKATETKIRKKVDFFKFILKCYIVSGITATSTRSIVPIFDKN
LTMPQDCWIPGNNSIVKHIIYAFQVIFYAESISYFTFFDGFLLIVTANLQAQFILLQKATGSINFETDSEETA
WKKLVKCCEHHKFLISVHKKLNTLYSYFYLVTYFLVITMGCVSLFVIFDKSSTFAQLLESAITMVVLNVMI
AMICICSSEIEIEAEKLLTQIYEVNWYETPNLKIRKFILFWLMQAQVSVETKGAGLLVVNRSLMLQVQRFS
YSVSTLLKGMNEZ
>TcasOR146
MKYIREVENIKAGIFSSCGIVAEEQRVEMSEKNQQDYFKIPDKCYNWSGVRVSSNALTKFVSLYILYPLML
ILYFMIIYNIRFKKNISDITEVFISISTFTIITFRKTLLIRNGSIYEDILKKQSHYWKYYMFGKPTEMKLRKSME
FCVMVIKFLIASTTGSIIFHSIISPIIEGKIVLPQPCWVPNNDPVANDIIFALENIFYMEGTNYLVVFDGLYLL
MTANLKTQMILLRKAVASINFREDEKTTWAKLKEYCEYHKFLLRIHGKINKIYSAFFLMTYVFTIMGTCTP
LFVIFYEEADMVLLGKSVFIALILNTLLVMTFIPAGELEIEAEKLSFEIYSINWYETKNLKIRKFVLFWIMQT
QIPVQMSGGGMLIVNRPLVLQVQRIAYSLTSFLAGLSZ

>TcasOR153
MAPNKSSKKDFLDMCRKYMHGSGLGPTSPKIKRFISLWLLFPVSLLLDVLVIYDFHFLDNDIFKTAELLES
VSSFGQLPIRKFILTYHSKLIQNLLEDRKKFWSYEMFGETYGKFLRRKMVLATRLIQTMIFFGASVATLMF
VSTLADDRKTVPLECWIPEFKHSTHVVLVMQFCSLCEIIYLVGAVDCLYVLTCVDIKIQFLLLQKKLKTIQ
VGVKPMEECLNELTICVKHHNLLLRSHKSLNRIFSEYFFVQYFVSVLAACVQLYILMYITASLEDIMKSIV
YLSAVVFQVAIFFMPASDIEEEAEQFAVEIYNVNWECTSGTKFRKQLLFMLMKAQKPLYMLGGGMIHAN
RNEYIVLFRLAFSISTLLGGMNENGRTDKZ
>TcasOR154
MSNNDETDYFKIPLKFYNWSGVRITSKKIPKIISVYILYPMMIVLYGLMIINIRFNIDSLAKFIEVGISVSTMTI
IALRKTLLIKNGSVYEDLLETQKLYWAYNRFGQAFESTQRKRMYFCLLVTKFIIGYSCVSLSYHSIIAPIIM
HEMILPQPCWNPGNTTIGRNIIFFVENIFYIEGTSNFVIFDCLYFLLATNLRIQFALLRKELNSIDFKEDSEEK
CFARLVKCSQYHKLLLSVHRKINTIYSAFFLLTYVFTITSTCTLMFVVFYMEADAALLGKSIFIVIILNTLLV
MTFIPAGELEIEGEKLAMDIYFMNWYETSSLKIRKFILFWLMRAQIPLQMTGGGMLVVNRPLVLQAERVS
YSLASFLANLZ
>TcasOR160
MSGKTKRITTKTIHLSNPYSSFKKVFSDFAYSKIMIFYTIATLAFHMLSLFLQIYYVATNYSVELICRYGPM
MCLAIYVVTAKVVGVFYYKTFTMLENQCLFVLWKTCNSSPTTQRLILNKSLKMNQKLHLALMSYFLLAI
VMLPTWGDLNELFIFSQVYERYFKFWAPVLYYFYISTFLWCSYYSFHLPGCILYLTLLLDVQIKLINDKITE
IDQNFSQNEISETLRLCISHHIALKRWMSTLAKMVNSVMPVFVLLGALSTVAVSFFVLNTLQNTTMILKIR
LAILTVCNFVIVSTFAELGQIFSDQNNSLFEHLIDCPWYLWNVKNRKILLMFMANCMKPKTFSWGGITLD
YSFAISILKTSFSYALILFKLRGETIRNZ
>TcasOR165
MSDNTKKATTKSLDLTNPYSSLKKVFINFAYSKIMIVYTSATLIFHILSLMLEIYYLATNFSVELICRYGCM
MCLITYMVTAKFFGMLFSNQFKFLEEQCLLDFWKAFNSGPTTQRLILKESSKMNRKIHLALTFYVILAIIM
LPIWEDVNDFFMFSQVYENYFANWAPVLYYFYISTFVWCSYYSFHFAGVIMYLTLLLDLQFRLINDKITEI
DQNSTQNEICGTLRLCISHHIALKRWMNKLANSVDTAMPVFILLGALSTIAVSFFVLNTLQSTSVILKIRLA
TITVCNLIVVATFAELGQIFSDQNNSLLEHLMDSPWYLWDVENRKTLLMFMANCMKPKTFSWGGITLDY
SFALSIFKTSFSYALVLYQLRGNTFZ
>TcasOR173
MSNVTFDEPFMFFKKVFFDFGYCTSIRFYHLFCFTFHICCQIIENYFFLTEYLSADFVTRYGCPMIVIGYTIV
CEFFLMKWEEPIKELLDERETIFWEIDSNSKPQILKYSSKVNRIYKFFLFWVVILAIFLLPFWGDLDETFFIIR
IQKIYFGKWSTLFYTLYVSTLPFMVYSGIRFPIVTLYLIMQSHLQILILSQKIGQISQNNNHMDDVSKFHDV
GYQKKIRTSLHVCMCRHVTLKQWISKILQIVQKAIPVYFSLAIIVLVTVMFCILYNVESASTTTIFKIRLVLV
GICGAVVLFTFSETGQLLSDDTSQVFDTLAASPWHEWDPKNRKTLLMFLLNSLKPVKIYWGGFALDYQL
GGSVIKTTFSYALVLFNLRKDZ
>TcasOR175
MRNFQDSDDPFIFIRKVFVGFGCSTIIMYYSRLIFIFHTLSLLLESYHVITNFSLDIITQYGSAMSLMLYSITSQ
FLLICEQNLITEVVEECKSFFWTMDFLSFIKQTQILKDMTKIKRKMYLSWIWFVVFGIALLPVWGDYNEMF
LFPFIYQTYFGNWSPLFYYFHASSFPFLAYIAIRIPAFILYLTLALHFQTLLLNQKILQIPQNKSGNQEDIFRN
LCSCISHHVALKKFVTKTQQSIQKMIPVYFVLAILCLVAVMYSCLNSLAMSTSNHFKVRGFFGGVCGVVV
LYTFAEAGQLQADTTGEVFNTLMQCSWYNWNNRNQKILLLFMVNSLKPSYIDWGGVIVGYSFGSSVIKT
CYSYALVLYKLKISKEQNVTFZ
>TcasOR198
MPNVTNKRQKRLFSKTRTKSEDPFVMIKDVFVDGGYHPVTKMLNYICLVIHSCSLLLELNYFVHNYHFDL

MMKYCCAMSLMGYIIATMLFAIFQEHSAIDLTKDILSLFWPIDYCGPRVKEEIVKKATKINRIHYIVLLFAG
ALGITMFPIWGDQKEWFLCVQVYQHYFGKWSKIPYYVYFFTYPMLAFSSVRLPFMTMYAIVQIRMQVYL
LHQHISEISGEYVYDMKNLQILCDQNYQNEIYDKMRLIISHHIMLKRWMRKLVHTVQISMPVFVLLGTMT
SISVLFYAIYSFHNINFILKVRLISVSVCTVLVVYMFSEAGQALSTETTGVFDLLMTCPWYVWNIKNRRILL
IFMANSLEPMTFSLAGVTLDYRFALGMLRTSCSYSLILYKLKTGIZ
>TcasOR199
MSMTRSKYFQDSDDPFSFIRKIFIDYGYSKKINYYNRVTFTFNTCSILLESYYMITNFSLDLFVRYGGALSL
MLYHVVTQFLVIAKQKSLEQLLEESKSYFWKADIFNSSVKNQILKSCNHMQRKFCLLWTPFVACGIVLLP
VWGDFTESHIFPQVYKAYFGHWSPIFYYFCISSYPFAVYTSIRLPAIALYLFLQAHFQIVLLNQKILQISKNN
DLDETTIFENMEYQKTIYRNLRSCISQHVALQKYITRILVSIQKAIPVYFCLAVLCLIAVIFFVLNNLNMSAS
NHFKARIFVSGVCGSLILYTFTEAGQLLADTTGDIFNTLMQCPWYYWNIKNRTVFMIFMLHSLNPLKIDW
GGFTLGYSFGGAVIRTCCSYAVGLYNLRESKYZ
>TcasOR200
MSIQNTKKTLNGKEKHPDFIKFCFTDSNDSFLILKRVYIDFCYHKITKTCNFLLVISQLFFYLIQIHFLLSRFS
LELLARYSTIMMITTVALFGLILSFYLEEDIHELYKILTEIAWPLDKASKKDQQDLRQKSRRINSLNLYFLGF
LAFMIVIFLPIFGDEENLFLCIQVFDEYFGDRAFIFYNLYFIGFPFLIYFSVQLCFMFLYAILHLHVQINLINHH
ICEMGASFELLSDWKKLHSVVYQSAISQLLCQCIRQDIALKRFIVKLNETVQLGLPFFLPVSGLCGISVIFFL
LNYMCTMSLVLWLRVSAFFICLVFVALIFSVSGQLLIDETGKIFDTLVKCPWHIWNVRNRKIYLICLTHCV
RPNCISYAGITLNRIFLITVFYKTVSNAFILYQVRNSZ
>TcasOR202
MVEFKDPVIMLKTIFLVNVKEMTKFSQVFLAIFTFYSLVHCVQMYYLYKNFDVNLLIKYAPATTATLFVS
NTKLLSSSLNIMPIFSVVSETKLLRITTFIDKTFWPLDSIRKEARIKLERKCRAINISIYCILLLLSVAVFSNFPC
FGRQDDFFLCIKIFKEYFGQWSSIPNYIYFTLFPIFCYPYFRIAFSFVYAILETQLQFSLIEEYLFEVYQMVDL
NWKYLQDPRYQQEIGKSLQLCIEHHTALKKLIHSIVNITLTGMPIFLLFGIGLFVSCFVFIINFGDTMTMILK
LKTPLLLYVATMLSMTLLMCWNGQQVIDVTSRIFYTLVRAPFYFWNLYNMKVLLMFITNCTRNENIVLA
GICLDYTLSVSILRISVFYTLGLLELRNHSFDZ
>TcasOR206
MWNNNPFIVIRTIFLDINNYKIVKFCYVSLTVFYSLVHCLQFYYIIKNFNLNLIIRYGFITSLLSYVLAAGILS
LVVEKRIRKTQIFFDEIGWSLNIVGKDAEMKLEKKCKLINISIYAIMLLLIITLLVNLPFVGSQRDLFLSIQVF
EEYFGKWSEILDRLYFALAPFLSYHGARLSFTCIYAIMQVQVQFSLIGEYLFETYQVDDSKSWKYLQDTR
YQHDIGESLRLCVEHHVALKKSIKMMVDIALTCLPFLVLLGLCTLISCLAFIMNFWDTMDNILKLRIFMW
AAWIVLITIMFCRSGQQLIDATSDIFFTLGGAPWYYWNLDNIKILLTFMANSTKNDSISLAGICLDYPLFVS
VANTTVSYALVLYNLRESSLDSSNKKZ
>TcasOR207
MVEFNGPFIMLRTILFIDMNTYKILKLFNVLLNVIYSLIHCLLVYHMFKNLNINLIIRYGPAVLFLILVIVGA
VFSVYLEKDILEIVTLFRKTRWSLSMIKKDARIKLEKKCKIINIFILFLVLLIISTITINAPYFGDQRELFICIQV
FEEYFGEWFFIPYNFFFVAFPFLYYNFFKLWMTFVYGILEAQLQFFILEEYLCGTFETDFRKNWEYLQDTR
YQQEIGTSLRLCIAHHINLKKLIKMIQNVTLMVMPFFLVLGVLILISSFSFIINFADTMTTIAKIRMVISAISM
VGITILLSWIGQQVIDVTSDIFVTLGGASWYYWNQKKYXKTLLMFLTNSIKNESIVLAGICADYGLCVALL
RLSVSYALVLFNLRKSTLVZ
>TcasOR208
MEKLDDPFITLRKMVFIEAKNCKIARFCDVLLIVLYSLAQCLHLYYMCQNFNLNLLIRYGPILISCLLVIVT
AVISVGLDKEIFEVYTVCWKISWPLNFLRKDAQTKLRRKCQIINRGILCSALLFLTTVISTFPCFGSVRDFFI
CVEVYEKYFGEWSFIPYYFYFAAAPFLYYHFFRVCYVFAYAFLHAQLQYFLIEEYLLETYQTNDLKGWK

YLQDTRYQQEIGKSLLLCITHHIALKKYVKISQNLVLIGMPFFLVLGVLLLINSFGFITNFGDTMSNILKIRIL
IFVACGVSITIVMCWIGQQLIDVTSEIFVTLGGAPWYFWNRDNNNILLMFLTNCTKNESFILAGICVNYQLF
FSIVRLTVSYTLVLYNLRESGFIZ
>TcasOR209
MMPEFSDPFIMLRKMIFIKNHKIAKFCDFLIIAIYSSAFCLQIYYLCKNFSISLLIQYSPTLLCYIFVIDAAVLF
FYVEKNILEAITYYDEIGWSLSMIPKDAQTKLRKKCLIINICVSFILLLILSTLTINLPYFGSQRELFICIQIYEE
YFGNWAFVPHHFYFGVFPFIYYNSVKMWISFVYTILEAQLQFILVEEYLLESNIINDFKGWKHLHDIRYQQ
EIGKSLRLCITQHIALKKLVKMIVNITMAAMPSFLVLGVLLLISSFAFILNFADTMTNILKIRVLMFVACIVC
ITTLLCWTGQQVIETTSDLFDSLVGAPWYLWNRENIQIFLMFLVNCTKNESLVLAGICLDYRLFVSMLRIS
VSYALVLFNLRKSSITZ
>TcasOR214
MAEFIDPFLMLRALVSVKFNDYTSLKLCNILLITIYSLIHCLLIHYMFKNLDINLAVRYVPMIMFLTLVIVG
AIFSVAIEKDILEAQVFLFKANWSLEMIRKDAQLKLERKCRIINICILCVLLLIFATITINAPLFGSQRELFICIQ
VFEEYFGKWSFIPYYFYFAAFPFLYYDFLKLWMSFVYAVLEVQLQLTLVEEYLFETYQINSSKEWKNLQD
THYQQQIKKSLRLCITHHIALKKFVKMTVDLTIKVMPFYLTIGVLILISFFSFIINFADSMSNILKIRIFMFSAS
IVSITVLLSWIGQQLVDVTSGIFWSLVGAPWYFWNLENVKTLLIFLMNCTKNESIVLAGICIDYSLGISILRL
SVSYALGLFNLRKSSLDZ
>TcasOR222
MDKRDDPFIILRKMIFIEAKNCKIAKFCDAFLILFYSLVQLLDIYYMSKNFSISLLIRYSPITIMYLLIIIAAVIS
VGLDKEIIEAYTVCCKIRWPMNVVKKQTQIKLKKKCQIINAGLSCTVPLFLVTIISTFPYFGSERDLFICVEV
FEAYFGEWSFIPYYFCFAASPFFYYHFFRITFVLVYAFLHAQLQYLLIEEYLFETYETDEAKGWKYLQDTR
YQQEIGKSLQLCISQHIALKQFVKKTVDLVLIGMPFFLVFGVLLLTSLLAFITNFEDITSNILKIRILLAAGCS
LCITIVFCWIGQQLINVTSDIFFSLGGASWYFWNRDNMKTLLMFLINCTENESVVFAGICLNYELFLSVVRL
TVSYTLVLYNLQKQZ
>TcasOR223
MTDFKDPFIMLRRIFIDVNSYKITKLCDFTVITFHSLVLCLQLYYMIRHFDVNLSIKYGPITAFFLFMTVSAV
LSGALSQDIFRAVAFFEKISWSLDVIRKEARIKLERKCQVINTCISCILLFSSTTMVINLPFCENQRYFFISNQ
VFEEYFGKWSVLLNVFYYSGVPYLGYHSVKPCFVFVYAILEIQLQFSLIEEYLLQTYETDYLESGEHLEDT
QYQREIGEALRRCITHHVQLKKLIDMMVDIVLMYMPFFLVLGVFLLITCFAFIINFADTTTNTVKVQAFMF
VVTALCNTVLFCWNGQQLIDVTNSIFLTLGGAPWYHWNVENIKILLMFITNCTKNDSIVLAGICLDYKMF
VSVFRISVSYALVLFNLRKRSLVZ
>TcasOR224
MTEAGDPFIMLRWILLMDVSNNKITKYCNVFLTTIYSVVLCLQIYYIFKNYDTNLLIKYGPITISLLFMITVA
VISLIMQKEIFKTVTFIRETCWPLNIIQKSGQIKAERKCRTINFYITSTFLLFLSAIIIHYPCFGSQRDFIICIEMF
EEYFGEWSSVLYYLYLIGVHFLYYRLFQTCYMFVYGMLEAHLQFFFLGEYLLETYQTDCLKRCKYLQDT
RYQQEIGQSLRFCIKHHIALKKLVKMALNLAFIGMPFFLVFGVLLLISCFTFIINFADTMSTILKIRIFMFASN
TVCIAILLCWIGQQLIDVTSDIFVTLCGAPWYNWNLDNIKLLLMFIMNCTKNESIVLAGIRADYQLFVSLLR
ISASYALVLLKLRKCSLVZ
>TcasOR229
MSARPLHLRNFPYYFLKVLVFDFEQYSAGKVLSYFCAIVHSISIFLQMHYLVKNFTKETMFQYGCVLTVL
TYCVVALFFAIASGNFVEKLESEISSFVWPLDICGEDVKAAILKRAFYTSLVAYITIIAFPIFSVIMFPVLGDQ
SDMFLCVRVFNEYFTKWSQIPISLYFYSFPVIAFSGIRLPGMLLYAILITHIQMFLLNRRIEQISELSNQRRVF
ETLCSCIELQAKLKRLIRNVFQLVYIAMPIFILLGAVSSVFVLFFVVNSLETASYFLVLRMGCFFGANVLVV
FIFSQSGQSFSDETGRIFDTLVMCSWYNWDKRNKKVLLMFLANSLEPMSITIAGITLDYKFALAMLRTSCS

YALVLYQMKNZ
>TcasOR231
MEVTGKKVTSGDPFITLKKLYIDLGYHKVTKLVNVFFIVFYGFVYLLQIYYLIVHFNFEIIAKYSTILLLSTY
LFNVMIFSIIYEKCILDAYKTFSQIAWPCDNASKPLQIIILQRSKTIKYLNYFFLGFIFFMACINWPWLGDQN
DFLLCIQVFKKYFGSWSPLFLLFYYLGFPIIGYSAARIFFIILYGVLHLELQIRLITELFCKISRNATLEDVRNA
KYQRDVYWTLREGIRHDTALKKVLFDLNKEVKHGIPVFLIVTLLCSVSIFFFAITYLESMGFGIQIRTIAFVG
AVIFVLFSYSLLGQHLINQTSLLFDQLYECPWYTWNVKNRAIYLNFMLNTVRPIKITYAGICIDSRFFLSITR
IILSNAFMLYQLRNSZ
>TcasOR238
MMSEFNDPFIVWRMIFMINFKKHKITKFCEIVLIVIFSLIHCLLLYYMFTNFSVNLLIRYGPTTLFGIFIIAVTI
FSVALEKELSGGIDILDEICWPFNMIGKEAQLKLERKCRMRNMCTAFVVLIILTTIIVSYPCFGDQRDFFICI
KVFEEYFGEWWSIPYYFYFITIPFFCYNYHKLCFTFVYAVLETELQFFLIEEYLLETFKMGYLKRWKYLEN
TQYQQELGKSLRFTIAHHNALKKMVKAIVNVTVNGMPLFLLLGFLLYISCFTFVINLADSMTNILKIRIFVC
GASCVSVTVLLCWNGQQIIDVTNSIFSTLTGAPWYFWDVDNVKILLIFITNCTKNDSITMAGICLDYKLFAS
LLRISFSYALVLFNLRKSSLSZ
>TcasOR254
MDKLDDPFIVLQRLIFIEAKNcklaRFFDVLLIVLDSLAHCLQIYYMCQNFAFSLLIRYGPVFIFFLLVIVTAVI
SVGLEKEIIETYAVYCKTCWPLNIVKKQTQIKLKKKCQIINRGILCSLVLFLAAVISLFTCFGSQREFSICVE
VFDEYFGEWSFFIQHFYFTVAPLLYYHFFRVCYLFVYALLQAHLQYFLIEEYLFETYQTNDLKGWRHLKD
TRYQQKIGKSLLLCITHHIALKKFVKMTLDLVLIGMPFFLVLGVLLLISSFAFIINFADTMSTILKIRILFFAA
SSVCLTMTFCWIGQQLINATSEIFWSLGGAPFYFWNRENSKILLMFLMNCTNNDSVVLAGICLNYRLFLSV
VRLTVSYALVLFNLHKSGLVZ
>TcasOR259
MMPEFNDPFLVWRMIFTINFKKFKITKFCEIVLIVIYALVHCLLLYYTFTNFSANLLIRYGPVMIFYIFMIAA
TTFSIALEAELSEVITFLDEICWPLNMIAEDAQVKLQRKCRIINMCIAFLVLIILSAIIVNYPFFGDQRDFFICV
RVFEEYFGEWSFIPYYFYFAASPFFYYNYFKLCFTFVYAVLEAGLQFFLIEGYLLQTYKVDYLKRWKCLK
DNRYQQELGKSLRLCIVHHIALKKLVKMIVNLTVNGMPIFLLLGSLLYISCFTFTINLVNSLTNILKTRIILM
GASCVGVTVLLCWNGQQIIDVTSSIFTTLVGAPWYFWNLDNIKILLMFITNCTKNDKIVLAGICLDYKLFA
SILRISFSYALVLFNLRKASVSZ
>TcasOR263
MWNNNPFIVIRTIFLDINNYKIVKFCYVSLTVFYSLVHCLQFYYIIKNFNLNLIIRYGFITSLLSYVLAAGILS
LVVEKRIRKTQIFFDEIGWSLNIVGKDAEMKLEKKCKLINISIYAIMLFLVITLLVNLPFVGSQRDLFLSIQV
FEEYFGKWSEILDRLYFTLAPFLSYHGARLSFTCIYAILQVQVQFSLIGEYLFETYQVDDSKSWKYLQDTR
YQHDIGESLRLCVEHHVALKKSIKMMVDVALTCLPFLVLLGLSTLISCLAFIMNFWDTMDNILKLRIFMW
AAWIVLITIMFCRSGQQLIDATSDIFFTLGGAPWYYWNLDNIKILLTFMANSTKNDSISLAGICLDYPLFVS
VANTTVSYALVLYNLRESSLDSSNKKZ
>TcasOR272
MTRTPTDDTTFDLDNFMKNDSMKLVRVIAYDTLKFKITKLILFITFLVHFSTTLIQVYFVCVDFNVYFFVK
YAPAMFGSLFVMVSIIALFVTAETDMVVRVFRKAQLRKLTVEDGPSFHVFQKECKIFTVFFVLNLIIALFSG
YLHALPDDDDREIFYAFAFFEDYCSEWKDFCSFLYRITFLPVAYVMYVPINVFVYAAIHLKSQIYYLKEHL
IQINEGYDISNNNDLFYDENYQRIIREKMIYLYKIHVKLFLAALDIRKLIRGFIALFAIVGCLLGISILYFVML
FQGNLFDKFGRLSTLTVVAFNSFAAVIISGQMIESSSSDDVDAIIYNCNWYDWNEENKRFFLLIRMATMHP
FKLQFSQNYAVNYQLGVAILKAMYSAFSLLKAIKNDFZ
>TcasOR276

MTMQFIVKRATRGIFHDLRVLKFISSDIFDIKIMKLCLFITFLIHLTACAITIHAFMFNNFSRREFISCAPVLFG
CFYGLLGLGTILFKPSMTRTLMLELKAWDITAADDAVSSRIKFEINVITVFCLVNYLLALVASFFYYMSFY
GDEEIFYLIRFLEDHCPNHKRVLIKLYKISFVLLGYVMVVHACQVLYATQHVRFQLILCAHFMANVTKQA
KNIKDEHLPDDNNYQNMIRERLKFCIIRHQEIRRFYFDKLEEMGNLIGGFALLGCFLGISFAMHMLTSEFLR
YHFARTVSSIIAGVTTFATVIAAGQSVETEVDISTRVVKEVKWYTFNESNKRSYMLMLLNSMQTYKIKFSE
NYSINYELGLSIVRGVFSIVSVVVQLDYZ
>TcasOR281
MDYSEKSLIQGDCLKLLKVISSDIFQPKLVKLILLIVFGVHLVVDLLTLRALLVNELDFKEFIFYGPVFFGSF
YGMMALLTLVLKDDFISNLKQEFRLWPLDCAGDEIYSQIKFENKIIKIFVVFNCIVTFIGSYLYFLPLDSDNE
TFYAVRFIEENYPDHRNLLHGLYRSTFLIFGYAMTVHVYQVIYNSQHLRYQIIIFTEYVASIGNPDKRKENE
LFYDKGFQKVVYERLKFCIMRHQEFLVISNKKVGDMRVFIVGYSLCGCLLGISLTFYIFSGKFYREHFPRV
SVACVGAVTTFWAVITAGQAIESEYDSLLSTLLGKIEWYYFNDSNKKNYLIMLINLMQPWKIKFSEEYAV
NYELGLAIVRAIYSIVSVIASMHFEAZ
>TcasOR283
MLKFEPKPTTKDELLWVVRTIYVDLFRNKLIQFALKMLFYGSIIMAIYQGVLFLYEFEIHYFVKYSSMYCF
TCFILLAAYSVPIIAEVATTAFTTIKCWKIDSGGALVENKIKQEAHFTNIITAINCIFGLMVLVLFIVPFEDDN
DFYFLFIAFEKYFPQWQQLLKWGFKAFFPCITILLQAPFYIVIYACLRIKFELYMWMEFLKNLNIVYEKSDI
CELVHDSEYQTEISKRLRFCIERQEHIYRSLLYGKKYVQQLDIYIFAYAILGSLGGISIIFVCISFEGNFFQGT
YLRLSALTFVTLTTFMHVIWAGQSVETTSSDSYDILKQCDWFLWNLKNRKTYLMCLNYTQRPLKAQFTQ
NVSINYVLGFSVVRTVYSTLTALNSLRKASKZ
>TcasOR286
MSVQKVSPDNLWLSAKICLHIFQYKAIKIILKILSLSIIILTCIQTFLYLKRFDSAYFMKYLPVYAGSLFILASI
FCIEHISHVILSTVEEFEFWDYADSKPEIRNWIKWEALYINTFMVVDAFVAYLSGIFHAIPLDEDYEIFYPLPI
FQEFFPDWTNVLGWLYRSSFLIVPVVMTAPSLMIIYFTSRLRFQMFLFMDILENISEGYDISEANDLIENSTY
QKEIKERLKTCIKRHNEFLSAGGQVMKNGQLFILIMSAAGVILGVSIIFFLFSFEGSFEKRYPRLVTLVISTG
LTFTHVIIAGQLVENIATRLYEILHFMDWHSWNQENRKILLIFMHNAQQELQIKFLDEVAVNYQLGISIGK
AVYSMISVLSSFKNLEESYNZ
>TcasOR287
MISFEEKHLTNVRESDVLWLGRIMSLEIVQYKPMRFILNIIAVSIIALTLIQTYLFLQKFDGLYLIKYASVYT
ASLFILFSIIAAPFLTKFSTEALNNLEYWPIESAGAQIEKQIQREAIYINTFFVVNMVVSLISGVAHMIPLDDD
KELFYPLAIFEEFAPKWKNWLEWGYRLSFLVVPVVMLNSSYVGIYTLSNFRFQISLFNHLLKNINFPLNDN
EQTIELMDDQKYQNEINKRLKFCIKRQTHLYKVAHYVTGKVKHLSFFVAILTILLLIAVIVFLFSFQGTFEN
RYFRIITLVLTAGNTFIHVIIMGNRIEEETEKIFENLKSLNWSSWNLQNRQVYLIFLHNNEEHFKVPISENAS
VNYELGISMAKTICSMVSVMSQLKNIDYSKNZ
>TcasOR289
MLNFEEKLDENIFKNDVLWLSRKLCYDYYNTKIVKIILFVLSIRVAILTLLQTLLFLQRFDGRYIIKYAPVY
AGSFLIFLSVKHIPLSLMLINSFKTITLWRIDSCGPEIERKIKKHAMWTNICLISCTVVGLVSGIFHAMPLEDD
EELFYPLAMFEEFVPQWKNLLSWMYRLSFLTVPFSMPIPVYIAIYVTTKSYFQILLFGHFLENLNTGFDTTL
NHLLIYNNKYQNTIKKRLVFCIKRHAYFSRAMNELIKKTYVTIATFSIMGVILSISVIAFLFSFQGNFENRYI
RVTTLVFTIVTVSTHILYAGQLIEDAAFQVYTTLKTVDWNSWNLENRKLYLVCLQNAQIIFSIKFTQDVSIN
YRLGFSMAKDIYSMISVMSKLRNVDYSKIZ
>TcasOR292
MQPNLQKNDILWLIRKLTFDLFQLKITKMFLIITSVSIILLTIIQTFLFLKKFNGYYFIMYSAVYTGSLFILVSS
LSVLPISKLIKTAWVKFSFWEINSATPKIERKIRKEIFYINCVVFFNTIVAIISGIFHAIPLQDDEELFYPLAIFE

TYTPEWKDWFSGIYRASFLPMPIIMVAPAYTVVYLCAHMRFQFCLLLHFLENINPDNENISDKKYQAQIKE
RLHFCIKRHIHLFSKSRPVLEDLKKFVFVLTLCGTIFCISIIIFHFSFQGTYEGRYPRIITIIIAASITFFLSILPGQ
LIENTSSEIFEVLRNTNWVSWNEQNKKLFIILLLNTRQIYKIKITENVSLNYELGVTMAKAMYSMISVMKQ
LZ
>TcasOR293
MISFEENIDHDIYKDDVLWIMRKISIDYFHYKVVKILLTLLSIGITILTVIQTFLFLERFEGRYFVKYAPAYIA
TFLMVVAMQYISFSIRLAALIKRITFWTINSARVETERKIKKHAMYTNIFFLGTVIMGVISALFHIMPLDDD
NELFFPLILFEEFVPNWKNFFSWMYRLNFLAVPFTLPIPIYITTYHLIKSYYQILLYLDFLKNINTGFDTTSSN
NIESAEYQQVTRDRLVFCIKRHSYFYTQMREVNRKMSKFIAIFALISVLLGGSVLTFLFSFQGTFENRYPRI
VTLILTAGCIFAHVIYAGQLIEEAATQVCENLKVLDWYHWNCHNRKLYLIFLQNTQKPYKTQFSQNVSIN
YELGLSIIKTVYSLISVLRNLQDINZ
>TcasOR294
MIVYEEKIDQDVNGNDILWVMRKVCIDCFQYKIVKILLLLLAIFIAILTLVQGFRFLERFDGPYFIKYAPAFI
RTFVILVAIAFISFGMETAIYVKDTTFWLIDSAGLESEKRIKKHAMYTNIFFVSYIVVGVISAIFHIIPLDDDN
DVLYPLALFEEYVPDWKNLFSSIYRFTFLTVPFTLGIPLYTAIYIITTVYYQILLFVVYVKNINTDLDVENVK
YQETIEKRLIFCIQRHSSFLKRMKESNRKMSATVLVFSIVGILLGASVLMFLFSFHMSFKIWYYRIITMVLPT
GAIFIHIIFIGQSLENAISQLEANLKMVEWYHWNIPNRKLYLIFLINTQEQKGVKFSQNVSVNYKLGVSIAK
AVYSLISLMSNLRSIDZ
>TcasOR295
MKFAEPQIQEEDYLKLIKICFIDVFETNLAKLALIVAFVIATGLTILQSYYSINNFTANQFLKYAPVYFGRFY
VLVCLVFIFYYTKVIFKIFDTVPKWKYETVSKKYNQHVRRDALYMNYFCLFSVLAGLCTAVLYVIPSEED
HEIFFIVSWFEDNVLEWADILSLCLRLSFLFVSYLMQAPCFQMCYLLRHIQYQMGILKFYMRNIHHGFENL
DKLIFDDSFHKEIKRRLEFCIKRHVTIVLIGTTMVREGRIFILLFAISGAMISVSLMMFLFLVERSLPFTYLRL
SALIFTATLTGIQYIAQGQSIQNTSEQWFRTLVRIKWYYWNRENQKSYFIILNAYKPFSIKFSQKLAVNYKL
GITIAKAVYSIMSFMRYITDHVGNZ
>TcasOR299
MNFDFRGEPKKQEEDYLELLKLCFIDSFRTYFAKFFLTIAFVNVIALTILQTYYSLTSFSTVQFLKYAPIYFG
RFHVLVCLLFILHYINVTDKNFQNIPRWSYTTIGRTYDNEVKKNALYTYILVACIVMAGFGSSVLYAIPTE
KDNEIFFIMTWFEDNLLEWADILSLCHRMSFLFVSVIMQAPCLQICYMIKHTQYQVGILKSILENIHQGFDN
LDELVYDDNFHKEIKKRLVFCIKRHINITLVGRKMVRDTGIFVLFLSISGAMLGVSLMMFFYVIEDTPTFV
NVRLCALIFAATITGMLYVIQGQNTEDISEECFQTLTEMGWYYWNEENKRIYQLLFINSCEPFSIRFSENMA
VNYKLGIELAKAVYSMMSFMTYVSNYIKNZ
>TcasOR300
MIGLTNGDYSPRPSMEGDCLKILKFFAVDIFNPKIVRFFLWIMLLYHVVFTLVTAYFMLYVLSNSEIIGYTP
AFLGNFYPMLCVWSVLFISRLIYVKEDMPLWAIDTAGAKVQASIKRKIFLYTAFGIFNLVLSLSAGSFYLK
NVSEDVNVFLALRIFRDYFPNYYQVLDLIYRLIYFCFSYLMVAPSYLLIYYILHVRIQAIIFAAYVAHIDGHS
DYGTDIDLFDNEEFQSEVERRFKFCIKRQIEFLLMESKKLSQISNLIAAFSLAGCLFGISIIFHLFTGQLIQEYY
FRIGLTSLAAIATFSAFIYTGQSTEVQIELVDNAIDNLCWYNFNRSNKLLYLIAKADLARVRKIKFSGQWA
VNYDLGFAIVKGIYSIISVVVSMWZ
>TcasOR301
MSFENEHLGIKLTHFLCVDLIKKRPVKFLKNLIFFQYFVVQIIQTYYFLKIFEIRFFVKYAPVYFGTYFLLFVI
IVSWFSENIDNYTTAQFDKWELENMTQTFDEKLYKKIKTPSMIVSIVIITNFALALVSGYFHILPDDDDKEIF
FIFIFVEEHFPKWQSVISWAIRSTYIFTAYFMIYPINTLSYFMWRLKFHMYFYLENIKKINEGRPQNEKNITT
CIPFQKEVRKRLIRCIKRHTEIAHLYTLTNGIFGTMILVCAVLGGLLMVSTAFFVLAFEGSFFKKIWRVGTLI

LCAAITSAGSTHSGESLETSGNNIFLYLKEQDWYIWDRENQKIYLIFLTNVKPLRIEFSADVGINYKLAVST
LRTVYSIVSVLSQLIKZ
>TcasOR302
MDFSEPAPFHADALWIVRLLAVDILNKPFVRFLMFTVLLFHVTVLLIQIYFICFVQTFGEFVKYSAIFCAMF
YVNLSMITLLYEKNMVKHILEKCKLWALNSIDDKIYQDIRREALFGTVFVITNLILVFVTTITFIIPAPLDRD
VFFVVYFFESYLDQTWGKILTLVYRATFLLLGFIVVTCAHQLFYTIQQFKFQIYLLKEHVENITNIEFFDED
DSLLLKNPSYQREVKRRVRFCIKRHIQILNGADVGMKLVKKWIPLYSIAGILFFVSIVFSCISFDGSLEDVYL
RVGSLGVVSVTTFCSIIFVGESIATQSDVLAQVNTFIRWCSLNRENLQLFAIMGIMSKEPYYVKFSQNLAIN
YALGIKVVKLVYSFLCFLIQCKGVLYZ
>TcasOR304
MRCYNHDVLWLLRFWCCDIFQYKLLKFVIFVMLVANTVLMVLQGYHFLINFNSLYFISYSPYWFGSFFIIL
SLTTCLTISNIGPEGIKSATLWQIKSTDPKLINRIKFQVKLITAYIIVNTVIALIAGLAHTFPSKNAEEICYVYK
IIEIYVPKWKTELCWMYKASYIVMALALPATCNQVVYGATHIRFQFYLVLDWIRNNIIESGCDDLKLPND
KDFQHKITKNIIVIVKRYTEFHRTFQAVNQRIAIYILLYAVIGGLLGISILMFYFKFSDTLVISDYLHAGTLTV
AAITTFMATVTSGQKLEDIFEELLYTWCSVPWYLFNKWNKQIYLMIMVNIKPINFRFTENSSVNYNLGAAI
AKTIYSMLSLLTQMSDKDVSTLZ
>TcasOR314
MEQLPKNDPLLVLRALPEILMQHKIIKYVVLFIICYMTVTMILCSYVLATVRGLWDLFWSQYSLLAFGSSI
GFSCYFVAFWKGSEFIKLRRRVFANYWPLTSLGEESFQKIKKLSIFANVFMVATILASLATSTAGLPWVGD
EYDIMFPVRVYTDYFGERAVPLLVPFYLAMYCTGFVMISTGFIFVHFALHLKFQFFLLNKRLDGLRTEPLV
NDFLYQNHVKEELTCCIEYHQKLLKVAKEMNDIVYYPIFIVVSCGIMFSVCLVFYMKNFKNSFVRGTTMA
MTGTLTTFGFGFTGQLMENESGRLFDTSVMLPWHLWCLSNRKLYHIFLTKCQYHVSFSSSGIINLNHTLFI
SLYTKITSILSFLLNVSKKNHTKZ
>TcasOR315
MTLVRKLQAAATNAFEIRIKDDILAELFNWPFLVLDSKWSTKFAVFLTVYCVFETLACALVYSTLDVNM
MGTYAIVIARFATTFCSFFSFFTKRKQYFEIINENFPHFWPLQSLGKSTFNRIKMRASSVKFYSFLNVVVMLI
GAVILISFTQDESEVYLSVKIYKDYVNKWTTGFVMFFYVSFIYIGLVVAAISFVLTYTAFHLIFQCFLLNQK
LKQINDSIVENEQKQAKFDEKYQSFIYKELISCVKLHQRLILFGKRINHLVYAPLLVYIFGGIVVGVALIYY
LKSSVQHIFTSLILLLIALINSTTFVINGQMLENEAENIYISLTNLPWYSLNVQNRRVVYVMLMQSQKIIHM
SASGLVSLNYQLTIVFFRCIYTGMTFLVNVGLZ
>TcasOR316
MTLMRKLQTAIRNLFEIQIKDDILAELLDWPTLVLFSKWPKNFAIFSTIYCVFDTLVCTLVYSTLDVEMLG
KYAIFIAKSTIALCSFFSFFAKRKQYHKIINENFPHFWQLQSMGESTFDQMKKIATTVKFYSCLSVVAMLIG
AVILILFTEDESEIYLSVKIYKDYVNKWTTGYIMFFYASFLYIGIVTAAVVFGLTYIVFHLIFQCFLLNQKLK
LINSYIVKNGQKLVKLEERNQNFIYKELISCVKLHQRLIYFSNQINDLLYAPIFMYTFSGIVVGVALIYFLKT
SIQYILTSLVLSIVSLIITTTFVINGQLLEDETENIIISLTNLPWYSLNVQNRRVVYVMLMQSQKIIHMSASGI
VSLNYQLTIVLFRCIYTAMTFLVNMGLZ
>TcasOR324
MNIKFLDKSGGFSMAVVYFVAEGVFHIKFIRLYTYSALISNSLGFLFMLYQFIVGAESLYIIKYGPVLTGCT
YVLVSLWGILFLRKTEEFKQEFHFWSEHNASKEIQNRIKQHINSVTVYVILNIVLAFTAGTSLILPNKDEIHY
HYFIKKLSELDTIPRGINETCYYFYKINFVLMFPIMTVNSNRLLYFSRKFNFQVKLLVERIETMAKDYNVN
DPNLFYNVRYQNDVKQKLKIFIRRQAYIAQYVAKMNKFLAPFIIMFAISATLLGISVLLLLVTATIYYNKY
QLILCGAIYISTLFCAIEATETVEMESVEIYNALLAQPWYSWNNANRKTFIIFLKNCEKPIQITKFSDIFYFNY
DWGISVFKKVYSLGSVFFNLRQYIDKZ

>TcasOR339
MMLFRNTEIRRFQTKAKDPLHFLYASTFGLFRVKLVRLVSIFTLVFHSVISCAFFCEMLYSFDPNLLIEYGP
MIFIFGSGVMAIAVFFYIEQNGRLLLRQSVGRLWNFDSSSNLYKKMKLESKYILQVVHFNNFLGVCMVFF
HLPAGGQSDKVFYGINLFRRILPQHLNFLKQIYYSTFPILTYMVTVNPYLLLYASSHMKFQVCYVNELLVK
MTKDYKDIDYNLLRNEKYQKTVTLGLKNCIHRHAILKFMDKKLNKLIHWPLLILMALSVVAIISLLFIAIV
TRQDVHVPSFGATMIFGIVTNSLAVYSGQEVINESMKSLSCAGTSRWTSWNFFNRKMLVIFMTNAQKPFI
LQSPFFVCEFAFVIKALKFVSSVCGLFFEVARRRDLGELZ
>TcasOR340
MPYVQKGRLNFKPTNPYFNFRRPFLGPEKTVHRHRVSHRNEKTKFRHLLIQFDTSIVIFQALAQTVYEKTV
LELLCEFYSLYWPLDNVNPGKFLKQFRFLYVISFVTGGMFITSVLLVSPVFKNEKDIFLIREMFHNWGQILE
VVFWAGLFFQTVWAILIACVLAYAIFGIKFQLSLLLYQIKGMKGLRHQSMVKEKLHSVIRRHVCLTGFVR
KVVKTYWGLLQVEVCMFLVVNISMLFFFINSFSRSDWQHNLRPLCITVTSFMLTTCLIVLCLQIPDMTGRI
FDTTLELPWHLWNSKNRRTLLIFMTNSVQPIYINILGLGRLNSSSVSEYVKMIYSTTTVLCSLREGKKZ

Sequences of GRs from coleopteran species used to reconstruct phylogenetic tree

>PbreGR1
MKFCWKSKQKDSTESFSYWKTIMQSLEEPANVYDTLIPVHVLMKSVGLSPCILVVENGKLRYRSSTLGSI
YSVLAILLFVGYYIYAVEERETNDETNKVVRSIDMYHLYGSMIVMACCIILNSVHQKTLIEAIDRLNDADV
IMAGYTKKIDWKTSRNNMTVYLLVIMAMLAAGEFMNCTMFLRQVATLNTYCLLMCYIPMLINGFVEAQ
FVCHILLLKQRFAVLNSELRRLITKQRPLPSFKIIKVGPPIVEEEPKTLVPSKLIHNARQLHCQLCEIGVLLNR
YYSKQILLDIGDVFIGFTTLAYYCFDGCMKLYMKDDESNLYNTVTTGVWTLVKLSRLLVLTLSCSIVKNE
AKSSGDIIYKIENKYQPELSPEVYAFGKQIIHWNFKFTAFDFFEVDMSLFYSAVSSATTYLMILLQLDIANK
QIEKSMENSENHSH
>PbreGR2
DYGYHVRNPHQKITENNIRVERLSNAGLETISHRGDEDEPDPELLERFDSFYATTKSLLVLFQIMGVMPIER
ASNGNTTFRWFSATAIYSYILYIAETIFVSIVFKERLLLILQKGKRFDEYIYSIIFLSILIPHFLLPIAAWTNGHE
VAKFKNMWTKFQLKYYRITGTAIIFHNLTVITYSLCIFSWVLGIAIMLAQYYLQPDMQLWHTFGYYHILA
MLNCLCSLWFVNCTAKGRVAKDLAQNLHNALESADPANKLAEYRDLWVDLSHIMQQLGKAYSGMYG
MYCILILLTTIVATYGCLTEILDHGLSFKEAGLFLIAFYCLSLLYVICNEAHHTSSKMGPEFRDRLLNVNLM
AVDYRTRQEVHMFLTAIDKNPPIMNLNQYADIDRRLITATVTSMATYLVMLMQFRSTLMRNAALAARRS
AMNLNRTATNATT
>PbreGR3
MVATFMVKPSRDTNIRYITPLFKLCTLLCIIPPYDFRNYKFTICIKYRIYRALVLVFLLGGAVYCFISKVLYT
FDMIQNTVVVVNVLAYISFFLINVTSVTSANFNNFKSLDKFMNELVSIDRKLLAYHITNKTKTSTWLWLEI
FLNHVVLFFVMLFDGTLWYLASDNKFHFYMLYENLQKYHTHILSHLMVNLIICLKHRYRIANNLLAEAV
KQRDVVNISTKILFKPYKDVYTVKNVIKIFIGLNRMIEYFNKIYGWTLLCIHINIIASLLVSLDFIIEFSSESNIL
RDKYGLEFILLMSMWSLMALCLGIFLASTANSTINEAQNTSNVSYKLLQYVPPSSTNPHDRELREDLLLLS
EQSSLRTPCFTAAGFFNVDYTMLFTLLSSITSYLVVLIQFSN
>PbreGR4
LCSSMKDVNKMSKITFKESLKPLLVLGSFLGVISFNLTELTRSNLRIVTNVLLVIFYIILTGHSLYERSLLDTT
LLLTTTDMMQVSTSTLQIVVSWVMSALIQNKMISFLKRVSEIDKAFRQLGVYIYYDAVHRTVIKRLIVRLT
LGFASIISQLFIYDYALAISQIGFNVTLYFAILINVVIVEQFYTFAKLLRVRFEILNQHIQQVQKCVDVTKENI
SYIKLTGPIGSKLSTLRIICPIHHELTKIAKLLNESFGVMLLMSFQASFVTIIVSLYDCSVYLRFFKMENVKEL
CGSIIMCCTYVLDCLCICYSCHSTVEAASKSGKLLHQIDTEDMDVKDQIEMFSLQIVNEKLEFTAAGFFTV
NYGLLFSIIGGITTYLIILIQFSADPSESKQ
>PbreGR5
MLDRRSIRITVQMKGLTTRPRDDAYKKEVDAREILYTCRKEGSLYTATIFFYLNGFSVILLFLKLATKWPD
FMQYWQDTEVFIRCKIILPNRYSYVLFLIIGAAIVEHTLLITYSMTAEEYKKYQTNNTTRFEHFLARDSHIF
KHISYTWYMGTFVKVISLANTFYWNFMDVFIIAMSIALAAMLKLINYRIRAARQVRAIIWTDLRRNYDRIC
ELSFRVEDIMSPLILTSFASNLYFILVQLHGSVK
>PbreGR6
ASILVIYIYYLVKGFLRRITSINDTLERTFEHVSLDDIQHGTQTINNLEETAADISLLHAKLSNVIRSFNEIFG
WQMLCVLLNYILLFMVTYEMGLRIASRHLQNINWQFTVWFICSMVYAAIAVSAITSICDETTFEARRTTD
LSYSYLYNLNLSKQHKEQASLVRILTTLVRQTRSDASITFSAAGFFYIDYSTLFTLIKLLMSYLIVLIQFE
>PbreGR7
PIYCNINEIRSKPFLMGKCKNMPSVERLEGRIVLINSTKIYTKLCEISRLINRIYNIQIFVSIMSRFVMITTQLIN
TYNTIRDPSGGDIVLYVVLFLYIGLHFSKIFMVTNVSASTSSKAKDIAIСIHDMWGLLPEFNSEIQNFSLQIL

HQNLKFTACGFFTLDQTLINSVIASVTTYFIIVIQLEKTF

>PbreGR8

ARLLNKAFSLQVLIVLAFDFVLFVVNLYICYSKFVDERFKNSSSFKETVTLAFFSVQILLHMISIATVCFYTQ
KQANQSLELCFKIKEAYDVQIAYEINIFVLQIQHWNIQFTAMDLFKIDMTLLYSIIRSATMFLLIVIQLNGTT
I

>PbreGR9

NSFNATFGWSLLFVIFFIIASCLLAVGFIVFDYSNSFGSVHWTGKQYIALSIIWTISFLIAAIVLGKLADDTTE
EFSKTSDICFELAYKLQHSNTTKEHLLRDNLISFAQFTFSTKPVFTAAGFFNIDYTM

>PbreGR10

NLDAIFHALKRLVLNLVELLLDLRACMLVCAKVNHTKNVLFKIKIDPQNEEVRNLIMVAVFKLMHDKLE
FTAYDLFNMDFEFIFSMFASITTYLLIFLQFDIDAAKTRYAVMKANLTQFQGEF

>PbreGR11

IYLMGQRIKHLRKKKFIILGIALVLVEFLIIVIPDCVFFVENETVIYLATSYYPIIPNGLMKVQLITLLYLLSQR
FGAVNMILESLKSKPKHEGKNKKKSFVRSHNNQPFK

>AcorGR1

RSDLDTTLLSMTTDIMQVTTSTLQIVVSWFMSAVSQEKTMSFLKRISDVDKTFRQLGVYIYYDAVHKSVI
QRLLIRMSLVLASTISQLFLYQYQWNVGMISFYVTYYFPILINVLIVEEFYIYTNLLRTRYEILNQHLIEVQK
YNDSCKEKISYIKLTGVIGSKLSTLRIICPIHHELTKIAKLLNEAFGVILLMSFQSSFVTIIVSLYDCSVLLQYF
NIEHIRELCAAIIMCCTYVLDCLYICYSCHSTVESANKSGRLLHQIDTDDVDVKDQIEMFSLQIVNEKLEFT
AAGFFTINYGLLFSIIGGITTYLIILIQFSADPNESKH

>AcorGR2

MLFSRKSKDHLVRCNVLTYWQSTLSGLIQPRNVYDSLIPLHLITKCVGLSPFNYNILNEKPLYQTSLLGSSY
SFAIMILFVGYYIYAVEERDESTDSNKVARSIDMYHLYGSIIVMSACIILNSYHQKTLIQAVKSLNEADLNM
AGYSSKISWKKSRNLIFGYFSITLAVLITCEMLNCTMFLRQVGTLTTYCLLMCYIPMVINSFAEAQFVSYIL
LLKQRFAILNDELRSLITQKKYLPTVKITKVGPVINREENRPKPIAKSLICVRQMHSQLCEIGALLNKSFSLQ
ILLNTGDVFIGFTTLAYYCFDGCMKLYLNEDGSNLYNTVTTGVWTLVKLSRLLTLTLSCSIVKNEAHLAG
HIIYKIDNRYESELSSEIYSFGKQIIHWNLKFTAFNFFDVDMSLFYAVVSSATTYLMILLQLDIANKQIEKSM
EINRL

>AcorGR3

MDYGNNTPIHCARNPHQRKIEGNVKAQRLSDAGLENSAKGDSEPDPELLEQFDSFYQTTKSLLVLFQIMG
VMPIERSAKGITTFRWFSGATIYAYSLFVAETIFVTIIFKERLLLVLQKGKRFDEYIYSIIFLSILIPHFLLPIAA
WTNGHEVAHFKNMWTHFQLKYYQVTGTAIVFHNLTLISYSLCIFSWVLGVAIMLAQYYLQPDMQLWHT
FAYYHILAMLNSLCSLWFINCTAKGRVAEDLAQNLHNALESPDPASRLAEYRDLWVDLSHMMQQFGKA
YSGMYGMYCILILLTTIVAFYGCLTEILDHGLSFKEAGLFLIAFYCMCLLYIICNEAHYTTARMGPEFRERL
LSVNLMAVDSRTRQEVHMFLTAIDKNPPTMNLNQYADINRRLISSTVTSMATYLVMLMQFRSTLMRNAA
IAAKRSAMNLNRTGTNATT

>AcorGR4

MYRPTDVNNLSFLGSNNNYKPKKSVYLEGASVFYQKNKVTQVAPALSNNAQWNNLPYSASEGGVVQEC
LKPIIMLERSMGIFPISVVPGGFSKVTLPWMIYSVFVFLLILSYIGYIKWDKVEIVRSTEGKFEEAVIDYLFTV
YLIPVVIIPIAWYESSRMASVFSEWMAFERIYQTITHKKLPLFMGNKPLLVTLGLPILSCGTMVVTHITMVH
FRIVQVVPYCFINAITYIVGGMWYLHCDLIGRVATVIANDFETALHHIGPSVRVAEYRSLWMMLGKLTRN
VGLGSCYVITFLCLYLFLIITLTIYGLLSQIQDGLGVKDVGLTITAFCAIGILYFVCDEAHYASNCVRVYSIR
MRCLTKAIYYL

>AcorGR5

MNRTIMSGEIFIKYPKAATIFAAICAILFCIVGFLGNFVTILALARCPKLISQATTAFVLSLCISDLIFCSVSLPL
IASRYIYERWILGMTCCKLFPVLLYGNVALSLLNMVAITINRYTIISYYPYYSKLYSKISILVQLVFIWAGSF
LIMLPPLLGIWGQLGLHPPTFSCTILEKNGKSPKKFIFLVGFALPCVVIIISYICIYLKVKKSTKKLRKHQVND
STRNKNSRREREDGRLTKLMLLIFVCFVFCFLPLMCVNVFDDEVRYPTLHVFASILAWASSVVNPFIYAAS
NRQYRSAYSKLFNVFRSSMTATDSRQLSNSHKSRGTDNKNNHQLAKVVPAE
>AcorGR6
PTIVNTTIKLQFHLYNLILITRTEIINANLNDRIASSTPASKFTAFYQMEKDIESTMKIHKKITDTSRLVNRIY
GFQELFSFALCFVVLLSEGYIVLYSLTVGEGEAENFGYTLFSSLRLVIFYLLQLLVDLRACMLLCAKINHTK
NILFKIKVEPENEESRNVVMVAVFKLMHDKLEMTACDLFNMDFSFVFSMFASITTYLLILLQFDIDAAKNR
RANPLVNSSTA
>AcorGR7
NNRIRTAAQTKLILWAEVRRYYNKLYELSFRVEEKISPIILISFTSNLYFILVQLHGSVKKRNSAMESVYFFF
SFGLLCMRTLAVCLFVGNVDEESKTSIKLL
>AcorGR8
SFQFYCNFILSLLIRYFVLLINERFHQLNLMIEHHPEEVINTEGDQKKNVYVANSKDLVKAYHLLLEQVNIS
NNVFGIPMLFLSFCFIVDILNMLLMWIVYSFQDNAQMKGVVFGIDMLILCCFSVVKDLITYLALVSSCDQT
AREAGYTSFACYKLLYELSKSNNLYDSYTSKEKLSGRYDITLLAIQSSNVSVCFSAAGFFVMDFSTFFTLIS
FIMSYLVVLIQFNDRL
>CforGR1
YYSFLYLLIQSNFTSSILYKYEDINRNLLKLRKANVLQSTTALRRIKKCRKMYHEMGKIIGILNDLFGFPLFS
IYGFCGCQILYLCTDVYVIVAIPTHMAAYSWPLNSALVTVLVVLTLWITSIHYDMVSSQSNTLVTTCYAIQ
KNLNIFCERRQELVDFCDQIEMRKVKFSAARFFTINRSVIFTHVSVAASYFIVIV
>CforGR2
MPYLSRTHLEIMTLARISSFAFIVITLGMQFTVMLCCDFVVRESEKLLVTCIDLQRRFPLDSAEYRELQSFA
DTLTKRKISFSAANFFKINRSAMFSMLGTTLTYFITSIQFIEKPNK
>CforGR3
MQFAVMLSCDLVMKESEKVLVLCVELQEKFPEDSAEYHELRNLCDKLTLRKIKFTAAHCFDVDRSTIFSL
VSNTVTYFIAIIQFIEKDSKHGGKN
>CforGR4
ELRRLQQYHNRLCDLIDKFNEVMGSVLLLAILYIVTSLLWDVSAVIEFFVKGCVIEGVRLDDLVLLDFLFW
ILISIGQTMFMARVGNAVVDENKRTLNYCYEFMNKLPSIPDTLVQRIRDNLRELANQVEARRPALTAAGI
VKIDFRILGIVTTNTVTYVVVVLQFL
>CforGR5
MKQSHKNVAVMKSILVVGSVLGVCSPLWATFKPKSMAYFYKIYSIGSAVFWILVTIWMIYAKQKYLSDSI
ISPVRFMDCAVHICLAVFSVSVILVSGYVHSDRLKTLFKHLDLLDERLTHDVMKSRRKIYYHFGTFNVFLL
AFMISDATIWISTLGLKWYSKYYLTRVIQLYQCFLTVFFANVLADQLRDRFETLNQLLVNTIKGSDCGFW
VYSQQFNVRLNNRRGVKEFSQIHTFLCEMVENFNSIFGFLFMFAALVSIVMILNFVVMMLAIFFFEIKIEGIE
YGTNLLVVCTMGIVIIIVQTVILAWIGERLSNEASKTHRISYSILNSISLLPYIETKEGLRDDLLLLSMQSVFR
KPCVRASGFFVVNYRMLGFMTATLTSYVIVAVQFLTQTPPKLKELY
>CforGR6
LLITLCCCGVSALLNFYFAIFGGFGQTSLIAAVHQESRTWADVFWALYFLLRFLDVCGAAEFLCRETDRTK
SIISELLHECRNNRVKEELWTFHTNNAASKVTFTAFGFFSIDFRLVSSAIATGTTYLVILAQFHS
>CforGR7
MLQVLRIAVFFSRYAKHRSVDNYDPMDKKPKIPINRRFPKKTLRTISLLYYKIVKVSNMLNQTFSFQIILML

YKSLITILESIDYCLSNATGITIWTDLFWSCATMLEVLIMFLLTTKLQSQVSPW

>CforGR63a

MFARELTISGHPHVVTNADDERNIYLEGSMPLFQKQSQNLVKVAPAPTDFYKTELRGGKINAFENESYEF

SIVINYLKPVLRTLRILGLLPVANSDRGYFVTIHWIVYSVLLLLFIMGYTAFIQLHKVEEAKAGEGRFEEAV

IDYLFTVYLIPIVTNVIGLYEAKKQAEVLNQIISFEKIYNSTTKKKLDLFLANKPLLLSIALPIIGSGIMVVTH

VTMSNFVVFKVIPYCYINVVTYMLGGAWYIYCNIIGKVATIIAEDFQQALKNIESSIKVADYRSMWMMLS

RIIRNVGNSFGYALLFLCLYLFFVVTLTIYGLLSQIQSGMGTKDIGLTITASSAVMMLYFICDEAHYASACV

RTNFQKKILLVELSWMDDDSQQEINMFLRATEMNPTDMCLCGFIDVNRNLFKSLLATMVTYLVVLLQFQ

ISIPDDSLPDNNNTTTT

>CforGR43a.1

MFPKYLEDLTLSEESYRILEPLVVTSRLLGLCPVTFKKIGTIYKLKWSAAFLIYSYASVVALASLTMVGLIS

DSRQEADAVRMKDRKTTYITACDFSTVIFIDLFSVVSIPYRMKNFWHMVGIWSDIDALAPLNGYYTSRRC

SIIFLMVAMAATTLLFCYDMFSYAVEASCRTCFLTNFTAYYLLYFMIFFQGVFIWHLLYFINIRIVSLNRCL

DIEKKRLKQGTKKIFVIPYKITNGANDDQKSRADSPDVAARIAELLKVYDRIRESVFIFNTSPSFGVVMLILS

CLIHLVVSPYFLLLEIIRRGSTLFTTLYLLWVIAHTGRLLMIVEPCHRCLSEITITKSLICELMRLYVDRDVN

KTLKHFLAHLSLCEITFEGCGFFVISR

>CforGR43a.2

FYILYYNLVFVGIFVWHLALFINIRLDSLHKQLIDERRYLSSKLTFVTSRKHLNSARRMIVWLSMYRKIKDS

VDVFNDSPSFGVLSLTLSCLIHLTVTPYFLLIELAQRGDWLFISVQLVWLAAHTSTLLLLIEPCHRCLNVTQ

LINSSVSSLLMLDIDEETRKAVEKFMTYMAHCSINFKASGLFTINRNLITSVAGVVSTYLVIILQF

>CforGR21a

VQWCLMDLKLNTVDQATAKEVQLFLAAIHMNPPKVSLRGYAVVNRQLLSSGIATIAIYLIVLLQFKISLV

NL

>CbowGR1

MFAGMMAFCTIFVLFADFTEGAPALVKARQFLSDIPGFIPVYIRAGETPLEDINPDLAEAFNYYAQKHGRL

AFGRSIDEKSDNTDFHSGFPEGDKLSDIDTVSLDDESIQEGDNNVTVTVNPKESQHIQKIPKA

>CbowGR2

MTSWCRMDKIMNESYGYPESLDKRLQLFPAIFLVLALTEYFLSVWSRYIRLTLTLGEKYDYEKYYSDTFP

QIFKFIPMNVVTAAYCSIITVHATLMWGLMDVFIIIMSIALALRFKQVSRRIAKHVKRATSETFWAEIREDY

HRLSILCKELDDHISYIILLSYTLNIFFILKQLYESLEIRSGTVGKVYYLFSFLYLLVKVGSVSLYGAWINDES

KEPADMLNSVSSACFNVEIKRLLAQINFDNVALTGCRMFKLTRGIILSIAGAVVTYELVLIQFNSATIDNY

>CbowGR3

MNILFVVTQLYKGLTTRRTTVDQCYFVFSFGMIILRLVGVTLCGSTVHTQSKKPLAHLVSLKSEFYNVEV

KRLILQIHCHDDSLSGNQFFTITRPLIFEMATVIITYVLFMLQASITIYS

>CbowGR4

MIMGLVQLLGVILSVSLISCLGKRLINFISLFGSGICCLIVGSYAYINDINYLDSPIPIVSNTSNATMENTATD

SLQWISVTFLVISAFLTYFGIKVLPWILIGEIYHNKIRATASGLSAGTGYIIGFLANKFFLDMVNNFTLPGVF

WFYGSVGVFGTVLLYFVLPETEGKSLFEITEHFAGGDKLRRSVRRKKLSGNINNAFEPEDMNGNGMNES

>CbowGR5

GSLSDTANSSKKIICNILQNSLNSSIKQELWIYLSLATTNKIQCSACSFFKINKTLITSAVSVGTTYLVILAQF

NDK

>CbowGR6

MLIGSALTFRLKQVSNRLENISRMKVNDPTQWRSVRKDYNRLTELCQTVNKRLSAIVIVCFLTNLYFLLIQ

LFLSLGKMDNFVEKIYFYMSFLLIIMRIVGVCILGGEVYEEWKNLSFFLNCVVTSAYNEEVERMTCHVVS

WELSLSGKNYFKISRGLILKIAGAIVTYELVLIQFYKNVIEQ

>CbowGR7

MTLLFIICNEAHSASHKMGSEFRERLLSVNLGAVDNRTRQEVNMFLTAIDKNPPIMNLNGYANINRKLISS
TVTSIATYLVMLMQFRLSLMRNAAIAARKAAASATTNATLST

>CbowGR9

MSCDGVERCADRIVTTCYMNLDILEKSPIREEILSFTEYVEQLTPVFSAVGFFQVNQKVLSSLFSAVISYFIII
IQFNSGL

>CbowGR10

MSCDRVEKSAQKLLATSHCLQASVQDDNIRTELFHLWEFIEELLPKFSASGFFRINQHIIPAFLSAMTSYIIIII
QFEM

Sequences of IRs from coleopteran species used to reconstruct phylogenetic tree

>PbreIR25a
MLLRIFFLIIYLCNTLHSQTIQNINVLYVNEEDNDVAEKSVDVVLDYIKRNSRLGLKVDIRRVSGNRTDSK
GILDTLCKNYGSMLEDRSPPHIVLDNTMTGLISETVKSFTKALQLPTISGSFGQEGDLRQWRDIDDSQQD
YLIQIMPPADVVPEIVRTIVINQNISNAAILFDNSYVMDHKFKSLLQNVATRHVIRPIQDEASIRDQLQNLR
KLDIVNFFILGSLVNVKRVLDAADSINYFNRKFAWHSITQDKGDLKCACKNATVLFAKPLTDTKYQDRL
GLIKTSYQLSSEPEISAAFYFDLALQAFLAVKNMINDGAWHSNLTYITCDDYDGKNSPIRNGLNLKKYFS
KDQIETPTYGPMYIASNGQSFMEFNMQLSAVSIRSGSSDKSLNLGTWKAGFNNNISLIDPEFMRNYTADI
VYRVATVLQPPFIMVDEQAPKGYSGYCIDLIDEIANILKFDYEIFTAPDGKFGNMDLKGNWNGVVKELM
EKRADIGLGSMSVMAERENVIDFTVPYYDLVGITILMKLPKTPTSLFKFLTVLESDVWLCILAAYFFTSFL
MWIFDRWSPYSYQNNREKYKDDEEKREFNLKECLWFCMTSLTPQGGGEAPKNLSGRLVAATWWLFGFI
IIASYTANLAAFLTVSRLDTPVESLDDLSKQYKIQYAPLNGSSSMTYFERMANIEAKFYDIWKDMSLNDS
LSDVERAQLAVWDYPVSDKYTKMWQAMKEAGLPNTLEEAVERVRASRSSSEGFAYLGDATDIRYLELT
NCDLQMVGEEFSRKPYAIAVQQGSPLKDQFNTAILQLLNRRQLERLKEKWWSNNPKAMVCEKQDDQS
DGISIQNIGGVFIVIFVGIGLASITLAFEYWWYKYRKTSKVITIQETPSQPNHKRKQKPENTEQTLAPRKLY
PRSRF
>PbreIRx
MRTLLVLLTAIVSVKSYLDDCSVGLLQDDDDQIKKIAFMAESEILGFENMIEPVTAFRSYDTLTVTCHMID
SGVIALFGPQSEDNAQIVESVCNNKDIPHMETRWNDQPGKLTNTLNLYPHGPTFSKALADLVEARQWDT
FTVFYEDNESLARVSEILKRERELSISIKQLDAMGTGNYRPILKEAWRSGHTRFVIDCKVDNLIQLLQQA
QQVGMMTSRYYYIITNPDLQTVDLEPYKYSDTNITGVRIIDPEAEATKRLVGMINQKQIDLELDPSHAQII
APHELRVETALTIDGLNLLRMSLDHLPEHTRCGPEIACQDKKGWIHGSTALNYMKMTSFDGITGLVKLD
SEGHRTDFNLDIIELSEDGLVKVATWNLTDGIKFLVGDDDYDLHDLSFVVITALTEPYGMLTDSQVSLVG
NARYEGFAIDLIHELSLLEKFNYTFIIREDKSNGSKNKVTGKWDGMIGDLIDKKADLAITDLTITSEREEA
VDFTTPFMNLGISILYQKPQKAPPNFFSFAEPFAFEVWLWLGGAYFIVSISLFIMGRLCPSEWTNPYPCVEE
PEFLINQFSLRNSFWFTIGSLMQQGTEIAPIAYATRMTAGIWWFFTLIMVSSYTANLAAFLATENPDIPFND
VYELVEKASKSNIKYGAKNKGATMNFFRDSNNDDFKKIYNYMIANEKQVMVGDNKEGVLRAEREPYA
FFMESVSIEYEIQRHCNLSKVGDLLDEKGYGIAMRKDSPYRHKLNTAVLKLQENGKISDLKRKWWEER
KGGGQCSGEVESQEAKPLTLKNVGGVFWVTVGGVAVAVVLVFVEMFLHVMKESIKHKAAFWAELSEEL
KFYMKFKGLVKPVRLKKGDSKSPDDSDKSEKSEQHEMQGEANGRSYGFLPELIKQPLE
>PbreIR93a
MLPVQFLIGLGFVNFAYCNSFPSLLTVNATLAVIIDKEYLGDNYEFIKTIIEDRINIVKQQKLKTAGLNVVY
SYWTNIYIKKEISVILTVASCKDTWRLHKMADAENILHLAICETDCPRLPVDKAFTIPMTVRGEELPQIIFD
LRSRKAYDWKSVILLYDDSLGRDFTTRVLSSLTVDTTELSTGSTSVSLIKLDDYNTGLDKSSIKSTLSTFTS
QISRSSFIVIVSMDLATYILQAAQSLELMDIASQWLYVVSDYKLDNDTTSEILSNLKEGNNVAFLNNASTS
STSCKESLDCHIEELLYSFARALDAATLEELELSSQVSDEEWDAIRPTKSERRTYLLNSIKIDLTKNGICGS
CLTWEIWTADTWGKEFSFPPHDKFKLIKVGFWKLSSGATMNNVLFPHVVHGFMGKSFSLVTLHNPPWQ
IIETNDRGGIKCTGLVFDIINELAASLNFTYTLIVLNGEKNEKKNSSYYGKDISYRMIYSVPDGVVRMIRS
KQVFMAAFAYTITDENKAVVNFTIPITTQPYTLLTATPKELSRALLFISPFTFNTWLCLLAAIGTMGPILYCI
HKISPVNAYHGISARGGLSSISNCTWYIYGALLQQGGLYLPYADSARLLIGSWWLVVLVISTTYCGNLVA
FLTFPNNDKPVTTIDELLNQRDTVTWSIAPSTYYEYEIKISNEPKYQALYHGSLQNVGNMDKMLTNIELG
KHVHIDWKLRLQYIMKRRFSLKGTCSLSLGTQDFFDERLGLVVSPDNPYLKRINREIKRLHQVGLIEKW
LKDYLPKRDKCFKTRSNSNVNNHTVNLDDMQGCFFVLFFGCFISLLLICGERFYHKYQIKRERNIVQPFV

S
>PbreIR8a
MKNYDFFDFAKLILLCVSIIASRVEAENAPVTFLVLHEQDQLSFITWFEDALKFIEQSREERPTLNLFAIET
SDNESVIDKACHGFSNGGEILIDITWTGSDDVNEICKDMNVPYIRIDVSISPFLKLLDKYLDERNCTDVAL
IFDDPRQVDEALYYWIDTQKSRMVITDTLEHASAKRLKRLRPIPNNYAIIGNTEVVLKLFGIARQEGLFRL
ADRWNLVFLDFNYKQILSHLLPYEYISLVTLSEEVCCSLLEIPNCVCPNNFITQKQFVIHTLTLVIDVVLKL
KRNNKAFTSIACNETKNDAEILVSFTEILTSHVDRNKNIEESNNALRLNLEGNIDMIENNVITKIGSYSDAS
GFSVESGKLIKPIRTFYRVGITHAIPWSYKEIDLETNTTTWQGYCVDFLAELAKIMEFDYELVEPERGTFG
ERDDDGQFDGVVGDLQRGETDLAITALIMTADREEVIDFVAPYFEQSGISIVMRKPVRKTSLFKFMTVLK
LEVWLSIVGALIVTGFMIWFLDKYSPYSAQNNKKAYPYPTRDFTLKESFWFALTSFTPQGGGEAPKSLSG
RTLVAAYWLFVVLMLATFTANLAAFLTVEMMQTPVQSLEQLAKQSRINYTVVESSDTHQYFINMKNAE
DTLYRLWKELTLNASTDDIQYRVWDYPIREQYGHILLAINDSIPVANASEGFRKVNERIDADFAFIHDSSEI
KYEISKNCNLTEVGEVFAEKPYAVAIQQGSLLHDEISKKNFRTTKK
>PbreIR21a
KPHTYILLFNVVTLFITGTAGAIQNLFGKSLTDKIYEKFFLKTATNDAVVLSDLLIHIFHNHITKCVPILLYD
EKKYEYFQVIEELVKRLNTSIIHGWVPKSDVKSIGFLKDNQEQTCCNYVLFLNDIYNYKTFIGKQSVNNV
VFVTNSSLWRVNEFLSSEESREFINLLVIAPSTGSKIKRKEPCYVLYTHELYVDGIGSSTTNILASWTNGSL
TKKGVKLFSKKIKNGFSGHRFITSVGHQSPFVIKRGPNEDGEVLWDGIEIRLLKMLAGFYNFTIDIKSIKE
DPHKSAAEQVVDSVKSGIANVGLSGIYLTNERLDKLDVSYPHSYDCAAFISLTSTALPRYRAIMGPFHWT
VWLTLTSVYLLAIFPLTFADRHTLKHLIKQPQNVENIFWYVFGTFTNSFSLGKDSWANSSKITPRLLMGFY
WIFTTIITACYTGSIIAFIMLPNYPNTVDTVKQLLAGRYRIGTLNRGGWEQLFSNSSDILSQKLLKKIELLP
DIESGLLNITKAFFWPYAFLGSRAQLDYIVQTNFTTTNKRSLLHISSECFVPFGVSIVFAKGTIYKDVVDK
GISYIIESGFMKKFEQDIKWDFVRSPTGKLLQANSGTSSKIIYTEDRSLTLDDTQGMFLLLGAGYLFGLLS
LVLENVGGCFGCCGKKRSRENNSTSSNARENSRRCMLKRNNAAMGQADDKEIEIDRITIQKNKLDALF
GEENVHDNNQNY
>PbreIR40a
MKLLIPLISCVYVINCIFSVERTNYLLRKYGGELDSAIADIVIGFPSHTVAIVFDSTLDEDLLSSLCDSLSVR
GISLYIFNISTIAKLEEYFSFLADTVSKYLPIHTLHFTNEKLAEHLLMEITENSFIRRNIYYIFYWGRNDIQR
YFLRNMQEAMKVILITNPRNDAYRIYFNQATSHRKHHLTMVNWWTREKRLFNHPTLPSAIDIFKDFKNR
LIYVPVIHKPPWHFVIYRNDTFQVLGGRDDKLLCLIAENLKFRYEYIDPPERIQGSSFSSNGTFEGVLGLI
WKREVEFFLGDVALTLERSNVVEFSFLTLADSGAFVTHAPDTLNEALALIRPFHWKVWPAIVLTFLISGP
MLYVLITFPNLWQPRFLIKSRSKLLCDCIWFTTSLFLRQSEREPSNSHKSRFFIILLTIAATYVIGDMYSANL
TSLLAKPGREKSINNLIQLEEAIKYEDFQLFVEKHSSTHSLLENGTGIYGRLWELMNTVQSRYLVNSVEE
GVKLVKDFSDVAVIAGRETLFFDIQRFGPINFHLSEKINTAYSAIAFQLGCPYVENVNKILMAIFEAGILTK
MTEDEYEKLGKQQIVEKKLEGGENSPSIAENEHRKIIEAVESRQKLKAINIRMLQGAFYLLFIGHVVAAA
SLLVEIIYDRHFKKIRSCQKEHFRCLRRVGRKALYYFEVIKDRLRRRFQRLLNEAIISTIEYTE
>PbreIR76b
MGLVDYVLISLCANLTCLDEKIEGFRSDHESILHKQAQLLRNYTLKIATLHNPPLSSIEKVDGEWRAGGIA
FDYIEILKEKFGFNYEIIKPPEDSLDPQNNGIVGMLSRNEVDVGAAFLPTFPYISKHIRFSVSLSKAEWVVL
MKRPPVSATGSGLLAPFTVEVWLLILISLFAVGPIIYLLIILQCRLCHDEGNIIYPLPSCIWFVYGALLKQGS
TLSPRTDSSRILFATWWIFITILTAFYTANLTAFLTLSRFTLPIASVSDIGTKKYSWVSHKGSAIEAALENDV
NFKSSLKGSHWEFLEEDAGNILENWVKRHDYMYIGEKPIVDHLMYRDYLSKINTHIAEAERCTFVITTW
TITNNLRSFGYSPNFPFVDLFDNILEHLVESGIVMYSLREGLPDTQICPLDLGSKERQLQNTDLIMTYYIVI
GGFIVSTIAFMCELLYIRCNGRHQSSDQRGPDALFVITKNSKGNKLNDNYLFPPPPPYHAIFKPPFPNSED

TRMRTVNGRDYWVIKTSDGNSKLVPIRTPSAFLFQYAN

>PbreIR75a

MTIISKKNVSDIISYEFWEMYGLNNRRSILTHTGAWSKFIQKKQQGAFYRDRKNVHGSKLKISSEMCNTT
TGKTLQEKYAFLKIEREINCILLNLIQYIHNFSIGIAQGERLDIISGFMEESLVNITDVIAQTYPFRKTFILKN
PVGGNKNILIKPFSPKVWYMFILLIFITTALLKILLSVLHAFEMVDDKSWSLVILILLSTICLEGSPIESHFLS
FRVFVYTCLLSSMMLYNYYSSNVVSILLTKPAPNIKSLADLRKANIKLGVENIPNYYKPESELLTTNDFNA
VLNLSEAIVAIKGGDFAYYTEPATRYKLLQERLTFLEACSLVEIDLVPVAFVTVMARKKMEFKELLKITYR
ILIERGFLNREIRRLLERKPVCLAHVDYTVLTLHDLAVAFIIYSAGAILSVIALLLEHLYVRIVNIYQKKTQ

>AcorIR21a

QADITDSEMKLRSVIITIFTINIAMAKLEKRALQKAHERSRIDKLMDKFLPRQDYDKTTSLAELFIQIFHD
YLSECVPIIIYDDKINQYYPLLDIVFQKVNISFIHSMVVVKNGKNVDRNFTYTPDTHCFNYILLVDDIFSSK
YLIGKQSTPKIVLITNSSQWRVNEFLMGDFARNLVNLLVIAPSTSPSITETDICYILYTHDLFVDGLGSSVP
KVLTSWRDGELKRRHVDLFASKMKKGFSGHRFITSVTHEPPYVIQRGFDENDGIVWDGIEIRLLTLLSQL
YNFTIDIKNFKDENFKSPTDKIIDNINNGIVNVGLAGLYLTVDRLEVGDISYPHSYDCAAFISLTSTALPRY
RAIMGPFNWTVWLCLTIVYLMVIFPLALADKLTIKHLLKNPEEMENMFWYVFGTFTNCFTFGKDTWTK
SRKLTPRVLIGFYWLFTTIITACYTGSIIAFITLPLYPQTVDTVAQLLAGRFRIGTLDKGGWEYWFQNVSDP
QSQKLVKYIEYLPDIESGLKNITRAFFWPYAFLGSRARLNYIVQTNFTTTSKRSLFHISTECFAPFGVGIIFA
KKSIYKNTIDKGISYLQQAGIVSKFESDIRWDYMRSPTGKLLQASGSTLKMLTVDDRSLALDDTQGMFL
LLGAGFLLGFFALISETLGGCFRCLKRKRCDSSISSIPSNPRLYSLPTPRESIDSIQFSNNIQWDFAGEKMKT
NINDNSRIDLHNFEKFFGEHINLIHYRRVSDRVNIAR

>AcorIR41a

MYLQINILANFIINKYFNNNNCLLIITDRNNNFEYNGNLSYVYVKLNGIEIPYNLVFRSYGCQGIVITCQSP
VSIFENLELGMKLGSDRFNYRKYLFLTVTNQLETSLDVLKSKAAEFVADILIVAVDSDESIFDLYTHKFAG
PKEKSTDITWLDRWYSINNSFLFDSNLYPDKLENLEGRPFRIICFTYKPYCIIDPPDGTDMLVALEYARKH
NMTPELVVDEAGEWGNLYDNWTGNGVVGNLAQDMGDIGLGALYTWEREYSFFDYSKPTMRSGITCIA
PAPRLASGLATPFVSFSMELWIMTLSSYFLASVALLIVLSVTISDENDKKRNVNNIMLSLSLAGRIFLLQSF
QKVPNLEQSRITFGLALILSLMLNTIYSSGLSSTMTIPRYYGTIHDADDLAASEIKWGATSTAWIESIDSDS
RKVFVHIVKNFQILTEKELAAFNDEDLAYAVEHLQGGNLALGSYISLDGIQRRRLLDEDLYWEYCVLML
RKNSIFLSSLNDVILAVTESGLLYYWEHQTVYKYMDMNMQKAVKMSLRANNPGGSNSIVKLNLDHVIG
AFTIWGVGILISIIVFICELIKNKYNRGDNKV

>AcorIR75q

FETMISWKYNTLLFTALCITVICSENMEFIEIFKDFLLMQKRPTKVFLYLCWPVKEKVLVARYLNKYSFSF
QFNTQLKLPIFKKSTEQLFLLDIRCTQYLNLLYMANLNKLYQQPYRWFLIVNESITLPHFLEILVDSQLYIV
QNASPAVYVISSLYKISKYSENFVENDVARWSRSLRFSYFHPVSAVRNRTNLLGMPMNISYVITNNDSLN
HLWDYRDKHIDGVSKLNYILSHYIIDAINVSGYFIVRSTWGYKNVTTNLYDGLIGDLQSGLAEFAGTASF
FTPDRLLIVDYIAPTTPTRAKFIFRAPPLSYVRNVFTQPFDKMVWYASFLLLAVISIIVYVIVKWEWSSSKF
QNISTKQNVMKPKYVDVLLMELSAICQQGSETEPRSGAGQISVIVVFLTFMFLYTSYSANIVALLQSTSDS
IQTVDDLLNSRIKLGVEDKPYSYYYFKIQTEKTRKAIYTQKVAPVGQKPNFMNVEEGIRRMKDEFFAFH
VECASGYKIVADIFQESEKCGLREIEYWQIIDPWMAVKKNSSYKELVKVAYRKLHESGIQNREYRRLYAK
KPICQSRGSNFISVGFIDCYFPFLIFGVGNLITIAVLFIEILYKSRKRFQQKIYI

>AcorIR75x

MLLTCLDFGLSITKHNSIINFLSSYAKSKNVMLTLHTCWNKDMFLDKLLDFSKIANYGNDYATFVSTHTH
IFQKYSFNNLLIIIDLNNCPKAWSSLLQMNQTDSFAAPAKYLIFIDNIADIESTIRNFTIYPSSDILIAEKHAS
DYKLHGIYRINAISDLIWENYGYWSCEHGLVEYLKKYPPSQRRHDLKQQNLKVMIRITNNDTWNHLED

FRYTSVDGFTKLSYGASKCLFEYCNANVTYLGTEFFGYKDKTGNYNGIVGALMRGEIDTSGSPMFTRIE
RVPLITYMTMQSPYYIKFILKKPPLSYVKNIFFSAFDYKVWAAVIVSFVVLVIVSLFIYNTEAKQAKINFQS
KLTISDVILVSLEVLSQQGTYMDFQRMSSRILILTFLVAFFFIFIAYSGNIVAMLQAQVELKSTKELVDSRLD
LGAEDINFMQLFLSQDTDAVGKQVYQKIGENGYYPLAVGMEKVRKGFFAYHAELAEAYYYMREKYTN
NEMCCLQEVEGYFQYLRGYSVTRKRSPYKEIFKTGLLKVDEYGLKLRHYNLWYIKPICHTKGSNVGSV
GLIECRMAFFLLIYGTLISMLFLLAERIIHYTQTTL
>AcorIRx
KNHRLPFTIKDAAIRHDDRNGLQLSHKTCNLLARGVHTIIGPNSYPMSRHVGVICSGKDIPQVLTRSYSS
DEDYNNFAINLHPHPPILEKLFVELLNKLQWTKFMIIYQSNQDLVKVHQLLSYKSNIYDIKLSQLILDDR
MSYRLMLNAIKKSGECHFVVVCDLLTLKRFLQQAQQVGLLTEKHHYVIYNFDMSNIDVEPYQYGGCEII
SVRFFDPYSTEIQDAFNAVDEELYSNYGIETDGQTLNLETALIMDAVKLIHTTLKEHMLPEHIDNQPLHCN
DSEAWWHGPSLRNYLNVANVKGYTGLIKFDPKGYRSDFEADIIELKSEGLTKIGTWNTTDGLIIERPEKK
DPLPDDLSDVRGRTLNVVSALTRPYGLMKQATTRLYGNDQYEGYGIDLIHELSKELGFQYKIIPQEDGVN
GSKDNKTGKWDGMIGKVMSGEADLAIGDLTITSERENAVDFTLPFMTLGITILYKKAEPVPPSLFMFTSP
FSPQVWLLLIVAWIFVSLSLFVMGRLSPSEWQNPYPCIEEPEYLINQFTFKNSFWFTVGSLMQQGTELAP
VGISTRMLAGVWWFFTLIMVSSYTANLAAFLTVTTLNTPFSSIDELAKQDEIKYGAKANGATAFFFKDSD
KPVYQKIWKYMNNNPDLMVKDNMLGVNRVLKENYAFLMESTTIEYITERYCTLAKIGELLDEKGYGIA
MKKGSAYRQRFNTAILKLQETGMLTTLRMRWWKEKLNGGACDERSSTATVTALDLQNVGGVFLVLGL
GAFFGVVMAVLELSMDIMRYVKHQKAKYKQQMREEMKFFIEFKRNVKPSRKTGQENEDAVEFPFNINY
MENYINENNQNE
>HoblIR25a
MLNLFYLVLFIASCDRLRTQTIQNINVLYANEEDNDVADKAVDVALDYVKRNSKLGVKVDIRKVVGNRT
DSKGMLDSLCKTYGSMLDNKGPPHLVLDTTMTGLVSETVKSFVKALQLPTISGSFGQEGDLRQWRDID
DAQQNYLIQIMPPADIIPELIRSIVINQNISNAAILFDNSYVMDHKFKSLLQNVATRHVIRPIQDDSAIRDQL
QNLRKLDIVNFFILGSIVNIRKVLDVADSISYFNRKFAWHSITQDKGDLKCMCKNATVLFAKPVMDTKY
QDRLGLIKTSYQLTSDPEISAAFYFDLALQAFLSIKNMINDGGWPSNQSYITCDDYDGKNSPDRKGFNLK
KYFGKDPTETPSYGPMNIARNGESFMQFNMQLTAVSVRAGSSDKAVNLGTWKAGFNNNMSLIDPESMG
NYTADIVYRVATVLQAPFVMVDSEAVKGYSGYCIDLIDEIANILKFDYEIFVVPDGKFGNMDLKGNWNG
IVKELMEKKADIGLGSISVMAERENVIDFTVPYYDLVGITILMKLPKTPTSLFKFLTVLENDVWLCILAAY
FFTSFLMWIFDRYSPYSYQNNRDKYKDDEEKREFNLKECLWFCMTSLTPQGGGEAPKNLSGRLVAATW
WLFGFIIIASYTANLAAFLTVSRLDTPVESLDDLSKQYKIQYAPLNGSASMTYFERMANIEAKFYDIWKD
MSLNDSLSDVERAQLAVWDYPVSDKYTKMWQAMKEAGLPNTLDEAVERVRSSRSSSEGFAFLGDATDI
RYLELTNCDLQMVGEEFSRKPYAIAVQQGSPLKDQFNTAILQLLNRRQLERLKEKWWTNNPKALKCEK
QDDQSDGISIQNIGGVFIVIFVGIGLASITLAFEYWWYKYRKTSKVITIQDNPQQSVQKHKSKPDNSKTM
ALGKLYPRSRF
>HoblIR76b
MALIDIILIGLCANLTCPGDKIPDLSKRTRSLLREQEELIRNYTLKIATVHNPPLSIIETVDGVLRARGIAFE
YIEILQNKLGFKYELVKPPDNSLRPEDNGIIGMLSRNEVDIGAAFLPPFPYLSNYIRFSTNLDKGEWVVLM
RRPPVSATGSGLLAPFTFQVWLLILVSLFAVGPIIYFLILLQSRLCKEDDNIIYPLPSCVWFVYGALLKQGS
TLSPHTDSSRILFATWWIFITILTAFYTANLTAFLTLSRFTLPISSITDIGTKKYSWVSPKGSAIEAALDIDDTF
KQSLDGSHGQFSEEDASIILDNWVTRRDYMYIGERPIVEHLMYRDYLAKIDMNIAEGDRCTFVITKWVV
RDNMRAFGYSPEFPFQKLFDNLLEHLVESGIVMYSLRKGLPDTQICPLDLGSKERQLQNTDLLMTYYIV
AGGFIVSTVAFAGELLSKKCSGRVKSRKATASKDLFTISKKRKKGVLNEDHLFPPPPPYHALFKPPFPHSE
NAKTKTINGRDYWVVKSTDGDTRLIPIRAPSAFLFQYDN

>HoblIR93a
MLPVQLLIGLGFVNFVFSNNFPSLLSANATLAIVIDKEYLGDNYESIKSTIEDYIYITKRDRLKHGGINVFY
YSWTSITIRKEISAILTIASCADTWKLFKRANVENILHMAISESDCPRLPPNEGFTIPTIVRGEELPQILLDLR
SIKAYDWQSVVILYDDSLGRDFVTRVLTSLTIDTKEVTGGGTAVSLIKLDKHNTGLDKTSIKNTLSTIAAR
LSGTNFLVIVSMELVTYVMETATALRLVDTNNQWLYVISNHKLNNKSISGILKNLKEGNNVAFLYNISTSG
AKCKEGMECHIREMLDAFSNALDVAILDEFELAGQVSDEEWDAIRPTKLERRNFLLDTMKAYLIKNSVC
GNCTAWQMQACDTWGKEFKTSSSSEFSLIQVGYWRLGSGPSMTDVLFPHVAHGFRGKSFPLISFHNPPW
QIIKINKTGGANCRGLVFDIINELAKSLNFTYTVIVLDNGQNVSGNNSNYNNEISYGVTYTIPERVIEMVQ
NKFVFLAAAAYTVTEQSKTMVNFTMPVSTQTYSLLAARPKELSRALLFMSPFTYNTWLCLLLSIFVVGP
LLYIVHKYSPANEYNGMNVRGGLSSVYNCIWYIYGALLQQGGLYLPYADSARIVVGAWWLVVLVLSTT
YCGNLVAFLTFPNSDKPITTINELLNRRDTITWSITPASFFEYEIKMSNEPKHQILYQGSTKGIKNFEEMIKN
IELGKHVHIDWKIRLQYIMKKQFLSKSTCDLSMSKEDFFEEKLGIIVAQDNPYLGRINREIKRLHQVGLIE
KWLRDYLPKRDRCFKTKGGSGVNNHTVNLDDMQGCFFVLFLGCSISFLLICFERLFYKYQKKREKKVV
QPFIS
>HoblIR68a
MCINVLLFYFLQIFVQESEDLLSPHNKTQKSLLQIKHDDCQTYIILISNGQQVSRLLRYGDRYRILDTRAK
FIMLHDHRLFHKSLHYLWKRIVNIIFIKEYVGMKKSGDTTIAIPWFEISTVPFPSPIHNVFVPKRLDIWRKS
KFRSGVNLFQDKTSDLRNQTLNIVVMKHTPATTNTLLVQDDTVRAVMGDGSKGFAGLEVEILAAVSKA
MNFKCKLYEAPNADVEFWGKKHPGGQYTGLIGEMIGGKADVALGDLYYTSYILELMDLTVPYNAECLT
FLTPESLSDNSWKTLILPFKLFMWLAVIFCLFFCGIVFHYLAQFHCNINRLKRNAQNSDEIFPKKNQTLTL
PTISIDQMNYNSKYFLMKQQFVTKRETNDPEGLYLFSDFSNSMLYTYSMLVMVSLPKLPTGWSLRMLVG
WYWLYCTLVVVSYRASMTAILANPAPRVTIDTLQELVNSKLSYGSWSEVNKDLFKTSSDPISQTIGNNFQ
IIRNSSMAVEKVSEGKFALYENVYFLKEAFVIRQLQVQSATNSTENQVTEQVSNKPGERNLHIMKNCAIN
MPISIGLQKNSPIKPRADKYIRKVLEGGLIKKWLDDVMQPTLNAERPSTLQTTKALMSMQKFIGAIVALF
IGYLIGFAALCTEIIYFHYIVKRHPNFNKYSRRIN
>HoblIR8a
HRRSIDRNWLYDLVSGQVLSLQCPEQQKGLPISLQASCREFTLKESFWFALTSFTPQGGGEAPKSLSGRTL
VAAYWLFVVLMLATFSSNLSAFLTVEMMQTSVQSLEQLAQQSRINYTVVEASQTHHYFINMKNAEDTL
YRLWKELTLNASTDETQYRVWDYPIREQYGHILIAINDSIPVANAAEGFRKVNEYTDFAFIHDSAEIKYEV
SRNCNLTEVGEVFAEKPYAVAIQQGSQLNDEISKTILDLQKDRFFEYLQAKYWNYSKRGECDNSDNNEGI
TLESLGGVFIATLFGLALAMVTLVGEIIYYKRKRTKKVYPPGKDIQSIRTVSVKPAIPDSITFGTTFKPVLP
DKDNLRVSPFTLYPRSRNRIN
>HoblIR75a
RQKIKNIKFRVQQYGLLLCRSSKSFHFVKEKLKNLNLRIDSNLYVALKSTVGEYEVHEFYNPGYKNGGT
LMSYNSFNYTQRGAFASNYLSGYQMRKDMRSVHLRVAFVVTKKVSAPLEYLKDQRTKQFDTSSKVNF
GIFGHMIDIHRYRWDVVITPAWFGSKRHGVEIGLANVLHQNLADISGSSGLMTPGRVEAFDFGVGTYLFR
TSFLFRNPYTYDDDVGEALVLRPFSRGAWLCAGLFALILGLSLKLVFQFETRVLKRTSQTYSLCSIVVITIA
AFCQQGEFYFRIDLQKLNDETTFSNIGSGLHYNSVCGRVLLLQLLVFAYVLYNYYTSSIVSSVINSKKKTL
DTLEELVKSDLDIGIERIAYARVLNATNNPILFHLNNEANANNTRISFFPQSEGIQKIREGNFAYLAETITAY
NDMDRVFSNEEICETVELDGARPTLIYIVGKKNGQYNELFSISYRQIVHSGLLQRQQKIFTNQKPHCLNAI
QFVQVGFGQLTSAFVLLTAGYGITLMILLIEIVHFRIKSGSYKNIQ
>HoblIR40a
YFLRNIHEAMKVILITNPRYDAYRVYYNQATSHRKHHMTMVNWWTKHKKLFKYPTLPASIDIFKDFKG
KIIYVPVIHKPPWHFVRYKNNSFDILGGRDDKLLRLIAEKLNFRYQYIDPPERIQGSSFSPNGSFEGVLGLI

WKREVEFFLGDVALTWERFNAVEFSFLTLADSDAFVTHAPDTLNEAFALVRPFHWKVWPVIILTFLISGP
ALYALITIPNSWQPRFLIKSRRKLFGECIWFTFSLFLRQSEKEPSSSHKSRFFIILLSIAATYVIDDMYSANLT
SLLAKPGREKSINNLVQLEKAIKLKGFQLYVEKYSSTYGLLENGTSIYGRLW
>HoblIR21a
LGSSAPRILTSWRNGSLTRPQVKLFSKKMHTGFSGHRFITSVAHQPPFVIKRGLDENDDIVWDGIEIRLLK
MLSRMYNFTLDIKAAKDDISKSPADKVIDYVNDGTVNVGLSGIYLTKERLENLDVGYPHSYDCAAFISL
TSTALPRYRAIMGPFQWTVWLALTITYLFAIFPLAFSDKHTLKHLLTKPEEMENMFWYVFGTFTNCFTFG
KDTWTKSPKITPRLLMGFYWIFTTIITACYTGSIIAFITIPIFPTTVDTIQQLLSGRFQIGTLDKGGWEFWFR
NSSDKNSQKLLKNLEFLPNIESGLRNITKAFFWPYAFLGSKAQLDYIVQTNFTTTNKKSLFHISSECFVPF
GVSVVFGKYSIYKDIIDRGISYISQSGLIIKLEHDIRWDFMRSPTGKLLQANSMNTLKLISVEDRSLTLDDT
QGTFLLLFAGFLGGMLSLIFERVGGCFKCYKKRRFSTSSSISSKPRSYDEPTPREKLDSIQYSYDEFQLLN
NVQENNRNKTDVDAEFENMHHINKELQKTSTNLIVRTFNFDKLFGEENLNSHHETVEDH
>CforIR25a
MIRLMIVWHLGVFLVFLGGSQGQTTQNINVLFVNEEGNTIADKALEVALTYTKKTTKLGLSVNLVRVVG
NRTESAGILESLCKRYGEMLESQTYPHLVLDTTKTGIAAETVKSFSSALGLPTVTASYGQEGDLRQWRN
LKENEKDYLVQISPPGDIIPELVRTLVLNQNITNAAILFDDSFVMDHKYKSLLQNVATRHLLDQINEDDKQ
IGLQLRDLTKLDLKNFFVLGSLGSIKRVLEAAMLENLFNRKFAWHVITQDKGDLKCNIKNATILFARPQID
AVYQDRLGTIKTSFQLNAEPEIEAAFYFDLALRSFLAVKEMLTDGSWKRNNVTNFVTCDDYDEKNSPKR
YGLELKKYLEKESTEPPTYGPFKIVSNGLSYMEFQMALAAVYYVRSGSSDKSLNLGVWQAGFDNNLTLN
NPKVMANYTADVVYKVVTVEQKPFIIRDEKAPKGFTGYCIDLIDEIADILHFDYEIEAVSDGLFGNMDEN
GKWNGIVKDLMDKKADIGLGSMSVMAERENVIDFTVPYYDLVGITILMKVPETPTSLFKFLTVLETEVW
LCILAAYFFTSFLMWVFDRWSPYSYQNNREKYKDDEEKREFNLKECLWFCMTSLTPQGGGEAPKNLSG
RLVAATWWLFGFIIIASYTANLAAFLTVSRLDTPIESLDDLSKQYKIQYAPVEGGSTMTYFQRMADIEARF
YEIWKDMSLNDSLSDVERAKLAVWDYPVSDKYTKMWQAMKEAGLPKTLEEAVSRVKKSMSTSEGFAF
LGDATDIKYLEMTNCDLTIVGEEFSRKPYAIAVQQGSPLKDQFNTAILQLLNRRELERLKEKWWNKNPE
KRTCEKADDQSDGISIQNIGGVFIVILVGIGLACVTLAFEYWWYKYRKNTKITNIASEMPRHHHTHKQIR
VKGSDNVLDQGIILKTNKLYPKGRF
>CforIR8a
MFSVRNKPLLLAISLLTFVACQEIKIVVLKQHGQEDVVKVLEETLNYTLNDLRVTVSHAAMEGNDEDYQ
TVCQSISGGVALILDLTWTGNDPAFHLSNNMSIPYIQSDVSIGPFLELLDAYLDARKATDVLVIFDHDRYID
QALYYWLDSTRLRMVISNLDKKAIQRINKIRPTPNSFAIIAATSNVSSIFNQALKQDLLKLPERWNLVFTD
FRYKNFDRDVIGDLPITVVTPDEDFCCDMLLREECQCPSDFDMQSQFIYWLSELVSRVVKTMLDDNLQF
PKVLKCNSSRFPEQTKQRFDEIVENVINESSTVIKRKGNSLTMNVKGIFEKNVNGTLEMSATYRNGEFNT
VKGGALNPIKAFYRIGITHALPWSYKEQDPQTGERYWTGYCVDFANKLSEVMGFDFEFVEPKSGTFGEK
VNGSWDGVVGDLARGDTDIAITAIIMTADREEVIDFVAPYFEQTGITIVMRKPVRKTSLFKFMTVLKLEV
WLSIVAALIVTGFMIWFLDKYSPYSARNNKKAYPYPCREFTLKESFWFALTSFTPQGGGEAPKALSGRTL
VAAYWLFVVLMLATFTANLAAFLTVERMQAPVQSLEQLARQSRINYTVVKNSETHKYFINMKYAEDTL
YRMWKELTLNASTDDTRYRVWDYPIREQYGHILLAINDSNPVPVASEGFRLVDEHLDADFAFIHDSSEIK
YEISRNCNFTEVGEVFAEKPYAVAIQQGSHLQDDLSKVILDLQKDRFFENLQSKYWNHSSKGNCPSTDD
NEGITLESLGGVFIATLFGLALAMITLAGEVLYYKRKGQREESKKKLQVAKVPKFDGLILPPKYTVAANK
VSKIGPAGAKHADIEGIKIAHISLYPKARNRITQVS
>CforIR76b
MGLMELIVSALCLNATCTDDNLEKYSIRKDRYFLLADQLKHENLRITTLMNGHLSGYEIINGSIVGKGVA
FDIIEILQNEYKFNYTVVVPDDNVFYENSHEKGIKNMLLNGEADVAATFLPMTYNETIRYSRSLDTAEWV

VLMLRPKESATGSGLLAPFTASVWTLIIISLLIVGPILYLVVFIRVKMCEDDRDAEFSLPACMWFVYGALL
KQGSTLNPKSDSSRLLFSTWWIFITILTAFYTANLTAFLTLSKFTLPINKPEDIVKKRYQWVTNRGNGIITF
MNDQQGILQELIGPPKFQIDSTDKDILHSYVSKNFMYIREKTVLDYIMYDDYKEKTKEGVQETQRCTYV
VTTFPITTLPRAFAFSPTFKYFDLFDITIQYLIESGIIEFKEKEFLPDTVICPLNLGNKERRLRNSDLTMTYNI
VGGGLIVATITFALEFAVHSYKRNGCCCRDPKDGHQRPEKITGHFPKGNGLETKWVSPPPSYHTLFNDNY
ANNYWYENNGVNKKQAFSMTPGIIGSKGSLVPYKTSIFLFQFTK
>CforIR40a
ARFCIALLSISATYVITDMYSANLTSLLAKPGREKSINNLYQLKSAMTSKGYRLYVEKHSPNLDLLENGT
GIYETLWTLMESQQKEYVVESVEAGVKMVKYCRNVAVMAGRETLFFDVQRFGPKNFHLSEKLNTAYS
AIALQIGCPFIEEINKLLMTIFEAGIITKMTENEYEKLGKQKNIVDVEAQENDLPNTKTETKRAAKATEDN
EKLKPISLKMLQGTFYLLFIGNVCAGIILASEILLFKNRRAILRNRNKRSLALNNIKRKVIFNIRELQLKIVR
AYEEFMRDEFTQILEYLE
>CforIR41a
MGSEMMTMHSFAMFYNMTVVPVVSNVEQWGEIYSNWTGNGVMGNLVEDRADIGAAALYTWENIYY
YLDLSKPTVRTGITCLVPAPKPSNRWLTPLKAFAGATWFAVILVYVTGTITGYLSELVTSLTSRSLKGFATV
FTTQKVITTVIKPFINQNVTRTEMLVGTLGKYVMGMVFVATTILVASYSSGLATIMTVPR
>CforIR64a
MANLKNALVFNFCVFCLSSVVAFVDANLISSFFRFKHLDRNIVLTCFTKTDRADLRRKLTNSTDVMVSV
LDLREFNFINSVVVKYKLGVVLDGDCRSSRSLLLKCKRYKCFDSTHYWFILRTSNNYADLLRNVYLNID
CDVKVAYPKESNNFPRGYVIDDVYNPASDRGGELKTRAVGSFDTSHGYHVTELGNKYFVRRNLTGVTF
KTVIVLPEAFKGTLEDYLLSDRDIQINTFNRFHSRLLHYCSDYHNFSMDIVVDKSWGYLQSDGTMDGLV
GDLERRKIDFGLSPLFVKVDRAKYVFYGRKTWNLRAAFIFRNPRSRKSYQIFIKPLTYQVWAGIIVCSILS
VATQKLSYKFDRHNRCVEYSWSFPILCAFGAFCQQGTQSNPNRFCGRIVIIFTLTLGYMVYQFYSASLVSF
LLNVPTTLITTLEGLMEHNFKLGCDDVLYNKDYLKYSTDNLTREIHRRILLRHGNGTGFLNPEDGLALV
KRGQYAFHVDVVSGYPIIERTYDETTICELREVQMFRVKEMHANYQKHSPFKDLIDTCLHRLAENGVLN
RELIFWHPRKPQCLRSKSTIYINTGLEDFYPALFILLIGMIMSLQVLAIELLWGHRNAVINVESVRRVFPYL
D
>CforIR68a
MLKTLVPYQCVTIITDKIYDDVFQPTWYERFHGLISFFKIQFYDEDNEFLFQNGTEQSLTMAKNDGCQM
YVILVADGEQVAELLKFGDRGRLFDTKSKYVLLYNTQLFEKHLFYIWGRLINVVFIRRNKPKKPGNGSK
KSPWYELSTVPFPFYFKDVLVPIRLDIWAQSKFRKAAELFKDKTFDLKNQTLKVTALAHIPSTLKISGDDI
GMKAANRATFRTSSPSDTNASFRGTEIEIVEAISNAMNFRCALYEPSEMATDGDVGGNFTGLLGEMTSAV
ADIALGDLYYIPYVLDYMDLSVPYYTQCLTFLTPESSTDISWKTLVLPFSGVMWATVLVCLLLISYVFHAL
AGFHVHINKVKEKCRKNGKVPNNVRKTPINFALEPQIFKFGIDTKYTLMMEQYQPVKEENEPEGLYQFS
EPVNSVLYTYSMLLLVSLPKLPTGWSLRMLTGWYWLYCLLLVVAYRASMTAILSRPAPRVTIDTLDQLTT
NKLRYGCWSEINRIFFKSSSDSVLRVIGEQFELVASSDEAVDKVSHGNFAFYENIYFLKEALAKRQQRYV
HFYLYYANKTNI
>CforIR75c
MIRMLGTDRIDFYDYFMPYYRFRSGFYFRNPGVVRPNFYEVLKPLAARVWYAVLVTGLVCCACMEVV
WLMEQHRTDYEHSFLHSVLILIGIYAQQGVYVTPKRMAGRIILLSLLILSLLLYNYYTSSLVSSLISTEPET
LKTIRELYESKLRVGMELQPYTVTFMLDRSKYDRYLDLLNRTKIYADGKPNFPSVDEGVRCIRDSDFAYH
IESVSAYPLIAKTFDQASICDLTEITLIDSDT
>CforIR75q.1
MLGDLYTGRADIAGTVTFTPADRLKHFRYLVSTTKELPIKFVFRAPPLAYSTNLFALPFDKNVWYSCGFV

CALGAVFIGVIMIWENKRKLFDDCEENKPNIWDVVMMQIAIACQTHSFYQPKSISGRIATLTILSTFTYVY
VAFSARIVDLLPTTSDNIKNIRGLYESNMDMGVNDNDYNRFYFTHRQDRTDEYWRKLIYEKKISSKNSD
RQNFINATRGMKLVQTSYFAFHVELSTANNFIEKTFTAQETCSVRMTDTIFRGDIPHLSTPKNSTYVEIFLV
GFRRLFETGIHSREHRHYF
>CforIR75s
MRWEIIFPVFLVFPAYGEPTFLDFLVEFIDADGGNLAVNLFECGKSDSVKMAKKFMYHGFLTDAVFIDSV
QETFPLRPGRQFFVINLSCNNSINLLNQMVLEHVFALPNRWLAYYKSEDYVLSYVLKALKIPVMVDSDF
SLIEIRSDGAARVKKIYKKHKTSDSFEVEEHGAGTNAIAVDSTFKRRHLDVTLNACIVLTHNNSVNHLTD
KREKHIDSIAKVNYVLVLALASIYNITLNFSVQPTWGYKNNQSEWNGMMGELVRKEADIGGTSLFFTKD
RVDLIDYIAMVTPTKSKFVFRQPKLSYVSNVYTMPFDRRVWLCIAFTIILIIATMYVLAKWEWKKKKYV
EFHDTSNSAELTDSLTEIVLVAFGALCQQGASALPYSAPGRIATICSMITLMFIYVSYSANIVALLQTSSNSI
RTLEDLLKSRLQVGVDDTVFNHYYFSTADEPIRKAIYERKVAPAGKNPNFLTIEEGVKKVRDGLFAFHME
TGAGYKLVGEIFMEHEKCGLQEISYLQVIDPWLAIQKNSSYKEMLKIGLRLLQETGIQERENILIYTKKPA
CTSKSSTFFSVGIVDCYFVATVLAVGLIASSVILIFEILVHVWIQSHKKYSQEFVN
>CforIR93a
MLFGVMFVICLVFNYAQADSFPSLLATNATLAVVLDREYLAEEYENVRLKIEEYLVYAKREILRHGGVN
VIYYSWTAINTKRDITAILSIASCYDTWRLFHNARSENVFHMAISEADCPRLPSDEAITVPILEAGQETPQL
LLDLRTSNIYKWKEIVLIYDNTINSDLLTRVIKSLTKPANKMSASGISLMELIDFATMDEIRLNIKYKLSTIS
STRVGGNFLVIVSYKLVDLIMEYAKQLNLVDIKNQWLYVISDTNSKISNMKKFKRLLKEGDNVGFVFNSS
VSSHMCEGGMVCHVEQILGDFILALDDAIVDEFEMAAQVSEEEWEAIRPTKLERSNFLLQKVKKYLVDL
GACDNCTKWTLQTSETWGREYQTQDYQSDSIAEILPVGTWRPSDGPSMKDELFPHIAHGFRGKNLPFVS
FHNPPWQILKTNQTGDVVEYGGLVFDIIKELAKNLNFTYTVEIVKTTNINSNLSKFTNETATSAETSSITTF
NIPRGILEKVHNKTVALGACAFTITEDNKKLINFTVPISIQLYTFLVARPKELTRALLFTSPFKGDTWLCLA
ATIISMGPILFYINKMSPVYEYKGAKIKGGLSTVQNCIWYMYGALLQQGGMHLPYADSARILVGSWWLV
VLIIATTYSGNLVAFLTFPRIDTPITTLEELIRYRETVTWSIAKNSFLDDQLKTSTDETYKLLYDEQIEIDDQ
KLLLDKIKSGKHVYIDWKIKLQYYMKQEFLISDTCLLTLGSDEFLDEKIALIVAPDTPYLSRINEEITKLHQ
VGLIQKWLEDYLPKRDKCWKRKSSVEVNNHTVNMDDMQGSFFVLALGFFIACLIIASEKLWFKQVTKQ
RENVHEFIS
>CbowIR6
MNLRTKMGLYKNILLFQLLLGYCEGQTTQNINVLFVNEEGNEVAEKALDVALTYLKKNNKLGISVDIRK
VVGNRTDSNAFLESLCSTYSSMLDAQAYPHLVLDTTMTGLGSETVKTFTQALALPTISASFGQEGDLRQ
WRNIDDNEKDFLIQISPPADIIPEIVRTLVLNQNITNAAILFDKSFVMDHKYKSLLQNVATRHIITAIKDGNQ
VVDQLSQLRKLDLVNFFVLASLKNIKRVLDAADSVGFFNRKFAWHVITQDDGEIKCVCRNATIMFVKPL
PNAAYQDRLGTMKRTYQLNVEPIISSAFYFDLTLHSFLAIKEMISDGVWKSVTNYITCDDYNTENVPKRN
GLNLKKYFNKESTESPTYGPITVLSNGLSYMEFQMQLTSVGVRDGASDKSTILGTWSAGFYNNLTIVEQ
QVMVNLTADVVYRVVTVEQKPFMFRDESSPRGYSGYCIDLIEKIADILKFDYEIATVDCFGTMDENGKW
NGVVKELMEKRADIGLGSMSVMAERENVIDFTVPYYDLVGITVLMKLPETQSSLFKFLTVLENEVWLCI
LAAYFFTSFLMWVFDRWSPYSYQNNREKYKDDEEKREFNLKECLWFCMTSLTPQGGGEAPKNLSGRLV
AATWWLFGFIIIASYTANLAAFLTVSRLDTPIESLDDLSKQYKIQYAPLNGSSTQTYFERMANIEERFYQI
WKDMSLNDSLSEVERAKLAVWDYPVSDKYTKMWQAMKEAGLPNTMDEAVAKVRASKSSTEGFAFLG
DATDIKYMELTNCDLTVVGEEFSRKPYAIAVQQGSPLKDQFNTAILQLLNRRELERLKEKWWNKNPEKK
DCEKADDQSDGISIQNIGGVFIVIFVGIGLACVTLAIEYWWYKYRKGSKIIDVREVAHNPTKPPTFPKQKF
SEHNPDNTKPKLPKRSKF
>CbowIR75q

MLNSLTLFMILFLNFSSAMKNYTEIINIVDELLTKQNIPSEVTAYLCWSKVLKANLFKRLSASNILTKIIATD
DIVDLFPSEYQIYLVDLDCEGSNEILKKAQRKKLFSRPFRWVFCGNIEQPLFNDLYFGVDSRIFFIDNAGSE
YHIKMPYKREKNSKKFTVNDLAEWNSLQGFTRFDEFAAARNRTDLFGMNINISCVYTDSDTLNHLEDY
RNIHIDPLTKLSWILVHHLMSILNASATVIFRNTWGYRDSNTSLFSGMIGDLQTGEAELGGTASFFTIDRID
VVEFYASSAPTYMKFIFRAPPLSYVTNVFTLPFHTYVWYCSFLLLVLIFFAIYVIVKWEWKDVVFREKLE
RMHDGSISPLRPTFFSVLLMEIGAITQQGTDSEPKSNAGRIATIFTFIALMFMYTSYSANIVALLQSTTESIR
TLEDLLNYRISLGVQDIVYAHHYFEVRIQLSIDSYPNQTPGLKWLNTQDLGKLWR
>CbowIR8a
MRNVKIILLENEGQDTILTWYRSIVQAFKSPIKFEEFLISVDGEEFDRERICQAFSNGAMMILDLTWTGND
LARTVSMEMDVPYLRIDVSLSPFFDLLHEYLNFRNSTDVALIFDDPSRIDQAIYYWIDNVQIAMSISESLD
AMAAKKLRDFRPTPNSFAIFAETKNMEKMFKIALEENLVTLPERWNLVFLDFHHKSFDRGLLKKMPVNL
LTLDAGLCCQLLNLNSYCECPSRFNTSKMFLKIALNMLVTAIEELFKDDFKFHDNIDCDSNFTKDNEESV
RKTFEEVLNKAVGNDNLIRLDNSSNLRLKTTGSIEIGTDVGTEVFAKYENEAITALRNKIVKPIKAFYRVG
ITHALPWSYQIKDPVTKKLVWTGYCVDFTAKLAEKMNFDYELVEPKKGTFGKKHNGVWDGVVGDLAS
GQTDLAITALIMTADKEEVIDFVAPYFEQTGITIVMRKPVRKTSLFKFMTVLKLEVWLSIVAALIVTGFMV
WFLDKYSPYSARNNKKAYPYPCRKFTLKESFWFALTSFTPQGGGEAPKALSGRTLVAAYWLFVVLMLAT
FTANLAAFLTVERMQAPVQSLEQLARQSRINYTVVSNSQTHKYFINMKFAEDTLYRMWKELTLNASTD
DSRYRVWDYPIREQYGHILLAINDSNPVANAEEGFKNVNEHLDADYAFIHDSSEIKYEISRNCNLTEVGE
VFAEKPYAVAVQQGSHLQDGISKMILLLQKDRFFEGLQAKYWNNSVKGDCPNTDDNEGITLESLGGVFI
ATLFGLALAMITLAGEVLYYRRKRKTKELNIKQSKVFPEKPLDVFPPKPLLLGNNQITIGNTFKPVNLKE
KIRKEREAMKISHISLYPRARKPINPFEIK
>CbowIR41a
LFLPGDNLLENLSHIYSMQELKYIADIVIVNREYKDEDSQGLYMSDNVFSLWTHSYRGMDENAKRMFL
DLWFSKNQSFMLDENLYPDKLVNQMGRKLEMATFQYEPYSIIGSSETESKGSEMVTCLTFARHYNMTPV
LVVNDEGYWGDIFDNWTGYGLLGNLVEDKADIGFSALYTWESDYYFLDLSKPLVRTGITCLVPAPSLAA
GWTTPLYSFSTTMWAAVGSMFFVCIFVQFFMHYFHAKIYDDTNQSLKLLDRSILCVLKLFVQQVVTTRE
TPPGRSGKYFMGLLFTFSLFLSSSYSSGLSSIMTIPRYGRPINTVEEFAESKISWGATQDAWTMSLKGVED
P
>CbowIR5
MGLIEFVVASLCLNATCEPEDAVVPGVSTHLLKLNELAEELKEETLTVTTFENGQLSGYISQNGSFLGTGV
AFDIFHILQEKFGFNYTIVLPDADIFMDGFNKKGAKSLLEAKQADIAVSFLPVIESFRNDVVYSRVFDIAE
WNVLMNRPKESATGSGLLAPFTTAVWILIIFSVLVVGPIMYLMILIRAKMCKDDNNKIFSLPSCMWFVYG
ALLKQGSTLNPKSDSSRILFSTWWLFILILTAFYTANLTAFLTLSKFTLPITDPTDISRKNYHWVTNKANGL
RDYIEYEKHDRLSNGRTLVQDIGKDRYYADMKDLDILEEYVKKRNMMFIREKTLIKNVMYRDYQEKTK
RGVDEEKRCTFVMADFPITMFSRGFAYTHDFKYAELFDRTFQYLIEAGIIQFKLRENLPDAEICPLNLGSIE
RKLRNTDLMLTYVIVASGLGIAASVFLLEILWRMSKAKYKRTRKRKATTWLEKNNNLMKAKCLHLHTN
SSPPPPYQALFRPPFYYSDRDGGQKKTINGRDYWVIDKSDGLREIIPIRTPSALLFQISN
>CbowIR2
MVTEFSCDKLGSFEFLKKLITQGIPTRILLIDKDELSYLFPPNCQIFIVNLQCENSTNILKKANSLKLFSFPF
RWIIYHHEPINETIFEESFLSLDILVDSDVTLLEENKNKSVSATKIYKRHRNHPLVIEKMGYWTKTAGLRD
DREEKIMVRRRKNLQQIPLNTCIVITHNDSLKHLTDKRDKHIDSIAKVNYVLVEHLSDIVNVTLNYSIQNT
WGYKNNKSEWSGMIGELTKNEADIGGTPLFFIIDRVDIIDYIAMTTPTRSKFVFREPKLSYVTNVFTLPFD
DYVWASTIALVCIISMVLFIILKWEWKKKDLPSEKDSSNVPELKDSLTDVILFSFGAFCQQGAPSIPFSVPG
RITTIILFVSLMFLYTSYSANIVALLQSSSTSIQTLEDLLKSRLQVGVDDTVFNRFYFPNASEAVRRAIYLQ

KVAPPGKKENFMSIEEGVKRMRQGLFAFHMETGPGYKLVGEMFHESEKCGLKEIQYLQVIDPWLAIQK
NSSYKELLKIGLRQIQESGLQTREVSLIYTKKPICTSRGSSFISVGLVDCYPAAVVSAGGAILALIVWILEL
GLYYRPYMWISVKKVFAKSHEKIPGSIEQWPEWPYLK
>CbowIR64a
MDPPRTIFTLKDLAESQLRIGIEDILIDRNYFVQTTDPDAITLYEKKIKGQSNSSGFYSPSEGIALVRNGGFA
FHVETSTAYPIIEEIFTNQEICELDEIQMYRTQPMHTNLQKNSPFREMMNFCMLKLVENGNMDRLRKHW
DARRPNCIESAKKQEIHVSLSEFCCSPIALTLGVCFSLIFLLVECSINYKERLKKVWTFKNHSKSQYPFME
>CbowIR68a
LYTYGMLLLVSIPKLPTGWSLRMLTGWHWLYCLLVVTSYRASMTAILAKPAPKVKIDTLQELVSSQLTC
GGWGEMNSEFFKSSDDPLVTTISQNF
>TcasIR40a
MRRDHGGDLVSASFDIVAGFLFEEICICFDKNTNINFLQHLLVRFVSNNIAIKLFNITTVEVQDKYFAFLNY
QVTNHLGANTIFFSSHKFYEHVLLEINERDFIRRNLIYIFNWGRRPFSRYFVRNIINVMKVFVITNPRNDTF
RIFYNQAVPYKKHHLEMVNWWQHGVGLFNHPTLPAKYNNVFKDFKENVFKIPVIHKPPWHFVQYGND
SIKVTGGRDDRILSLLSKKLNFRYDYFDPPERIQGSSASENGTFKGVLGLIWKRQAEFFIGDVALSHERAN
YVEFSFITLADSGAFITHAPSKLNEALALLRPFQWQVWPAIGVTFVVVGPVLYAIIALPNAWRPRFRVRSH
ARLFFDCTWFTTTVLLKQTGKEPSSSHKARFFIIILSISSTYVINDMYSANLTSLLAKPGREKAINNLNQLE
KAMATRGYDLYVERHSSSYSLFENGTGIYSRLWQMMNRRQTHFLLESVEEGVQLVRDSTNKAVIAGRE
TLFFDIQRFGASNFHLSEKLNTAYSAIALQLGCPYIEEINKILMAIFEAGIITKMTENEYEQLGKKKQTTSE
TEKELIPGVKKENRRVAKVSEDNEKLQPISIKMLQGTFYLLCIGNIFSGFILLAEILVYKHRKTYKHKKRR
HRFVYLRKIRHSVASKFGAVVDAVRRVYRRAMHDAFVATLEYLE
>TcasIR21a
MQRGLIVLKLCLTALALKSLDKRALQKSHEKSQLEKWEDKFLNRDPSFDQTASLVNLISKVALDELSGCS
ATILYDKFTETSSDLLLEKLFRTFPIPYLHGQITDKYHMKVPKLQTSQDTCTGYILFLKDVMRSKDVVGP
QTNNKVVLVSRSSQWRVYEFLASEQSQSFMNLLVIAKSEKIVSSSIARLICLALHLKFGTALAIYAPNGGK
SAVYPSVIANVPKLGFRSAESVTSVITQNGANLGIGGLYITDTRLKATDMSHIHSQDCAAFISLASTALPR
YRAIMGPFHWTVWLSLTLVYLFAIFPLAFSDKHTLRHLLDKPEEVENMFWYVFGTFTNAFSFFGKDSWS
KTDKFATRLLIGFYWIFTIIVTACYTGSIIAFVTLPVFPATVDTPEQLVRGKYTVGTLDKGGWQYWFENST
DPITQKLLTRIDFVPDIESGLKNTTKAFFWPYAFLGSRAQLDYIVRTNFTTINKRSLLHISSECFVPFGVSII
YNKNALYSKIIDQGVLQAVQSGIVDKIKNDVEWETMRSASGKLLAANSYGKSLKALTVDDRALTLDDT
QGMFLLLGIGFLLGGASLLSEWMGGCLHLCKGNRNQSATSIQSNYRSHEVPTPREKLDSMQFNSFENHK
IEEEIVEERNCIIHRQDDDDIEEHINRLFDFEGVFGEANPDSRTGPEEELSFKNTTKAFFSLYAFLDSRAQL
DYIVRTYFTSMNKRSLLHISSECFVPFGVSIIYNKNALYSKIIDQGVLQAVQSGIVDKIKNDVEWETMRSA
SGKLLAANSYGKSLKALTVDDRALTLDDTQGMFLLLGIGFLLGGASLLSEWMGGCLHLCKGKRNQSAT
SIQSNYRSHEVPTPREKLDSMQFNSFENHKIEEEIVEERNCIIHRQDDDDIEEHINRLFDFEGVFGEANPDS
RTGPEEELSEENGKK
>TcasIR93a
MLLELVLSSAFVCVIRGDSFPSLLTTNATLAVIIDREFLSNEYEVIKHAIESYLVFAKREILKHGGVNVQYY
SWTTINIKKDVTAIFSIASCPDTWRLFRQARDANLLHMAISESDCPRLPPDEAITVPLITRGEELPQLLLDL
RTRQTYNWNSAFILYDDTLSRDQVTRVVKSITAQYSNLRVNAAAISFVKLETRLPMDEIRRQVKEILSSV
SIKTVGGNFLAIIGYELVELLMEYAKMFGLVNTRTQWLYIISNTHFRHKDINRFRQLLSEGDNIAFLYNNT
VNNDTCTGGIQCHCEEILSGFTRALDEAILFEWETSSQVSDEEWEAIRPSKLDRRNSLLQGIKTFLLQRGQ
CDNCTSWLMKTGDTWGREYQQNGTDSGGLISVGNWRPSDGPSMSDELFPHIVHGFRKRNLPIVTFHNP
PWQIIRSNESGAVSEYAGVIFELIKELSKNLNFTYTVELAKIGQEFSANLTKNEAQVVTNFIPDSILDMIRN

KSVAFGACAFTVTEESKRLINFTSPISTQTYTFLVSRPRELSRALLFMSPFTGDTWLCLSASIVSMGPILYYI
HKYSPVYEYKGLSKRGLSSVQNCIWYMYGALLQQGGMHLPQADSARIIVGAWWLVVLVLATTYCGNL
VAFLTFPKIDIPITTIDELLAHSGTVTWSMPKGSYLERTLKYTTEPRFRYLFDKKVEVGNFKNMIEDIENG
KHVHIDWKIKLQYIMKQQYLDSDRCDLALGLDEFLNEQLAMVVSQDTPYLEIINDEIKKLHQVGLIQK
WLTDYLPKKDRCWKNNRHIVEVNNHTVNMDDMQGSFFVLFLGFLLSFFITIGEKLWHKYVTKKKMKII
QPFTT
>TcasIR64a.1
NKISLILVILSKTETYIIKSCLSNAIVDFAILANVAFSLRISCYKLFMHIKLIANVFYNQLDQVLNRNHYHLA
VIIDSGCIDYADFAIQDKKYFYETYHWLVPTTPQNLNNSLNFLQKSPLNINSDVNVAILNGEGTKWSILDV
YNPASSHHGQFTVTKLGLCDETNGYQAKIAGNKYWSRKNMTGVQFKSAVVVPDPSIKLNDYLTSDKNR
QLHSMHRFQSVTVNYCREMYNFSLEIQRTNSWGYLTPNGHFDGLVGLLERRLVDFGSSPLIYKLDRMPV
IDYSYGNWVLRSTFIYRRPKIIEASYKIFLRPLSRTVWICIVLMMVLLMLFLKVVFSREKRLLQKRNLVDS
SWSFLFLFTLGAFCQQGATCHPQLLSSRTLSIFVFLFCILTYQFYSASIVSYLLIDPPRKINNLKDLSDSNLR
AGIEDILIDRNYFVQTTDPVAIELFNKKIKFSNNNSGFYEPWDGLDLVKQGGFAFHVETSTAYPIIEETFTN
EEICELEEVQMYRTQPMHTNLQKNSPFREMMNYCMLHLVENGLMYRLRKYWDARKPMCIESAKKFTF
NVGLKEFSSGLIVLSYGILISLGLLLREVIVHKK
>TcasIR64a.2
MSPPLPFMILLSVLTQTHALLDINLIENYFTEKSIKSATVFGCFRKTEQLNLVKIFSRGSSPISVLNLNQAGV
YQSIKSNHQQIGVVLDGDCPESESFLITVSPGFTHIAPNVVFISVRSTETXFDVKHHWLILSKSIQFLEKIK
NAVVNINADIHVAVQSGTNWTIFDVYNPASEHGGSLKYTRVGFYSRGRGYNAQTNEAKYWRRKDMTG
VTFKTMVVLLVPFEGPLEDYLHNDDNRNINTFNRFQNKLLRFCRDYYNYSMIVELGSSWGYPFPNGSFD
GMVGAMEKKLIDFGSSPIFVREDRARVIDYGRNTWSWKAGFLFRSPKSRTSIEIFLKPLSTSIWLITGVLA
TASIVILKMVTTFERNRYHSTSETSWSLSFLFTLGALCQQGSPWVPKMACGRITAISIFLLSLIIYQFYSASI
VSHLLMKPTNKIRNLKDLTDSSLKVGCEDIIYNKDLFAHTTDKVLKDLYAKKIYGKGNTSHFFPPEKGLD
LVRQGGYAFHIEVARAYPIIETTFPDNAICELREVKLFKNTDLYNTMQKGTPFRDMLESCFQRLAEQGILD
REKKHWHPRKPECIQSSQAFVTFHVGLDEFYPALLVLLIGIVISLTVLVVEKQIHIAREKMEREKGVVF
>TcasIR64a.3
FQLRVLMERLFFLSVLAVIIYTTNCTDNHDIITSYIKEKSVKYATVFGCFTKKEKINLVKIISHICPISVFDIN
RLNIENRMESRHFHTGIILDGDCPSAEKFLINCGRSYLFDVKHHWLIVASSEKIREKFNNVILNINADINVII
PEKPSNWSIIDVYNPASQHGGVLNFTRVGFYNKHDGYKIKYTGVKYWNRKNLTGVTFKSMVVVTYSKT
XKNSAYTIFQLPVPFEGTLQHYLDSDDNRDVNTFNRFHSRLISFCRDYYNFSLDIEVSKSWGYTNEDGTF
DGMVGALERKIIDFGSSPLFLREDRARVIDYGRNTWILRSLIKQQFRIISNWGFSAAFIFRNPKVRTSLEIF
LRPLPSSVWLITGLLAIVSIIILKLATSFERRRYVYDVETSWSISVIFTLGAFCQQGSPSTPKMACGRIATFFI
FLLSVLIYQFYSASLVSHLLNKPLTKIKNVRDLLLSPLKAGCEDILYDRDYFLHTTDKVAKELYAKKILGK
SNSSNFHTPEAGLKLVAEGGYAFHVETATAYPIIESTFQDQAVCELREVPLFRTQPMHANFQKKSPFRDMF
DTCFQRLAEHGLLVRERKHWHPRKPECIQSSKSIRFNVGLDDFYPALVILLVGIVASLLILVIEKEFRILTEN
PA
>TcasIR75q.1
SFLGTILTVYKQLAEKKIVLNVLTNHWKINQTKLSQHTFLVGDTLCPQFNSLLSHVSKFFCYQNSQQTLG
QIITSSXKWLVFDQNSTVNTNDLLLDSNFAVASQISNGRFHLKLCYKRAPNETIKFNEIGVFSNGFEYYNH
FIPTRNRSDLSGVNITVSYVVTKPDYPFDVEDYRFRHLEAFSKLSYAMVYPMLEMLNCTKKFIQRSSWG
YKGANETQFVGGMFGDIQNGTAEIGGTVSFYTVDRMSVVDYLSVTTPSDLKFILRAPPLSYVNNLFTLP
FDTKVWYCLYFIVGVTVLILYVIVRCESTYENALERRNNIDNIKPKFFDVVMLQIEAITQQGSENEPKTMS
GRIAVFIVFLVLMFLYTSYSANIVVLLQSTSANINTLQDLLNSKITLGVEDVVYSHHYFETQTEFTRKSIYE

KKVAPKNQKSNFMTTEMGIEKMKDEFFAFHVETTAGYKQIMDTFQEHEKCGLIEIDYLNVLYPSITIRKN
SPYKEIVKVNFRKIYESGIRHRQLNRIYYKKPHCVGKGGSFKSVGIVDIYFSVEIFAIGCFMALWLLLLEV
LFKKKIKFLVQ
>TcasIR75q.2
MKILIVFICLLINETTQNNFTDNLIVNTFNFIKILNVPVKISAHICWTRGKFDSLLMKLYXTVLANTIHFIKS
ISDKYNTNLIKNVSPKYANPEHQLFIIDLKCNDSLSVLQQAEKFKLFKSPFKWLLLGNSESLPNLYFGTDS
QIFVTEPRSQLDDIKTIYKYSPMVPRFVQHSFDRFYTNTKRTNLMGTTIKISYVITNLDSLNHLWDYRLQE
LKKKLYHFLICRNSHIDAINKLNYILVHNLMDFLNASRQFTMQPTWGYKNSTTGLYSGMAGDLQKGLA
DLGGTPLFFTPDRIDIIDYIAATTPTYMKFIFRAPPLSYVTNVFTLPFDSAVWHYCFVMVAVVVVCIYVIV
VWEWKETKFEEKDTHSHIDTLRPNIFDVVMFEIGAITQQGTNAEPKSNSGRIITIFSFLTLMFLYTSYSANI
VALLQSTSDSIKNLEDLLNSRIKLGVEDIVYAHYYFENAQEPVRKAIYQQKVAPKGQKPNFMTAEEGIRK
VQQGFFAFHVELSTGYKIIGEVFQEGEKCGLKEIEYVNLIEPWLATQKKSPYKEVMKIGMRKMHETGVQ
NREIRKIYTRKPQCHSGGSNFGSVGLIDCYSAFLTFGVGIAFAFLLFVMELIVRRYFIRREKERLK
>TcasIR75s
IVLPMINDLIEHFNKTQIILAYLCDKNGTNLLLIRNNNNTNFRRLSGSEPLFXKKLYQVNVLSPNSRDMPY
PTPPAFLTYVLDAGCSNTKQLLLLVPVITHXLIFGNNILKASEQKQFATPFKWIVYYNNPVELSFFIDEYFT
KTNILVDSDVTLATINPTSGTFDLNKIYKRKINGSIIIENIGIWGRGLGVTDTGYEKITYKRRRNLTKTVLK
SCIVITNNDSLNHLTDKRDIHIDSIAKVNYVLVQHLSDTINASLEYSVRGTWGYKDNKSQWSGMIGELTR
NEADIGGTALFLTSDRIRVIDYIAMTTPTRSKFIFRQPKLSYVANVFTLPFDASVWASVCGLLVIIAGLLYV
VVRWEWKKKDYVQVVVFFAFWVDFPSSVFCRTNRTSRKFTILGSXVFITFGALCQQGSSSVPFSIPGRIT
LIFLLVSLMFLYTSYSANIVALLQSSSSSIQTLQDILNSRLDVGVDNTVFNHFYFPNATEPIRRAIYQQKVAP
PGQKPKFYPIEEGIRKMRQGLFAFHVETGPGYKFVSEIFREDEKCGLQEIQYLQVPDPWLAIQKNSSYKK
MLKVGLRLLQENGIQEREVGLIYTKKPQCLARGSSFISVGLVDCYPAAVVLAGGIGAALAVLILEIYVHQ
RFVGFLL
>TcasIR8a
MVISENLDKTTANRLKAIRPIPNNFAIVATSSNMEELLQTALDENLVTLPERWNLVFLDFQYQQFDKKRL
KNMPINLLHMDEEICCRFLQSEKCECPHDFNLQENFLSLATNTLAKILKTLTMENLLRADLNCDDSRYSE
ATRTRFYELLQQEVDSNDLVFKENFGLHVNINGVIETGDEKVAEYNYKTGVTVLDGKKVEPITPFFRIGIT
HALPWSYKETDSSGNTYWTGYCVDFTEELSKLMGFGYEFVEPKSGTFGKKRDGVWDGVVGDLATGET
DLAITALIMTADREEVIDYVAPYFEQTGITIVMRKPVRKTSLFKFMTVLKLEVWLSIVGALIVTGFMVWF
LDKYSPYSARNNKKAYPYPTREFTLKESFWFALTSFTPQGGGEAPKALSGRTLVAAYWLFVVLMLATFT
ANLAAFLTVERMQTPVQSLEQLAKQSRINYTVVKDSDTHKYFINMKHAEDTLYRMWKELTLNASTDDT
QYRVWDYPIREQYGHILLAINDSNPVANASEGFRIVNEHTDADFAFIHDSSEIKYEISKNCNLTEVGEVFA
ERPYAVAVQQGSHLQDEISKTILNLQKDRFFEQLQAKYWNHSGKGSCPTTDDNEGITLESLGGVFIATLF
GLALAMITLVGEVLYYRRKSKIQNSETKKPKTVQTSENWKTDTLMPVSLINKDKQSVTIGTEFKPVNRN
RDLSEFGHITLYPRARNRITQTSNE
>TcasIR25a
MASSSAIIYRIAIYSRIATAHLNYSDFLNNVLTETHKMLKLVAFILYCTNLANGQTTQNINVLFVNEEGNLV
AEKAVDVATNYIKKNNKLGVNADPVKVVGNRTDASGLLDSLCSSYNEMIANSMNPHLVLDTTMTGLAS
ETVKSFTAALGLPTISASFGQEGDLRQWRNIDENEKEYLVQISPPADVIPEIIRSLVLSKNVTNAAILFDDSF
VMDHKYKSLLQNVATRHVIAPIKEADKIGDQLRQLRKLDIVNFFILGSFENIKRVLDAADSVGFFNRKFS
WHAITQDKGELKCNCRNATITLAKPLIDAQYQDRLGLIKTSYQLNAEPEIAAAFYFDLALYSFLAVKEMI
ADGVWKRNNATNYITCDDFDGKNTPRRAGLNLKKYFSKEVSETPTYGPISIVSNGYSFMEFTMQISAVG
VRESSSDKSVPLGSWKAGYDNNLTLVDPQIMKNYTADVVYRVVTVEQKPFIIKDETAPKGYKGYCIDLI

QRISEILNFDYEITPVGDQKFGNMDENGKWNGVVRELMEKRADIGLGSMSVMAERENVIDFTVPYYDL
VGITILMKLPKTPTSLFKFLTVLENEVWLCILAAYFFTSFLMWVFDRWSPYSYQNNREKYKDDEEKREF
NLKECLWFCMTSLTPQGGGEAPKNLSGRLVAATWWLFGFIIIASYTANLAAFLTVSRLDTPIESLDDLSKQ
YKIQYAPLNGSSTMTYFERMANIEAKFYEIWKDMSLNDSLSEVERAKLAVWDYPVSDKYTKMWQAMK
EAGLPNTLDEAVKRVKDSRSSSEGFAYLGDATDIRYLEITSCDLQMVGEEFSRKPYAIAVQQGSPLKDQF
NTAILQLLNRRELERLKEKWWSKNPEAKKCDKQEDQSDGISIQNIGGVFIVIFVGIGLACITLAFEYWWY
KYRKGGKVVDVQAKHSDVATKINDGFHAKINKLYPRSRF
>TcasIR41a.1
TKMLFNNFCINILVNFIINNYHKNSRCLLIFTDGDFDYKGEIPTVRIKATNGSFNSYLIFNYHGCQSVIIYTS
NVTALLIKFETEIRLKMERFNERKFLIVPQNPSEDFDKFFNLKQLYFISDLLLVLPTHNDTIFDLKTHKYVG
VIDNNEPVLLDRWFSQNQSFLFGKNLYPNKLQNQLGRPLKMATFTYEPYSIIGNVFEQFFENDFILQGKS
VGEHHGSELMSAVQFALKYNMTPVPVINEKDYWGDIFPNWSGNGLLGNLVDDKADVGFSALYTWEFC
YHFLELSKPLVRTGITCLVPAPKLSERWLTPLFSYSSYLWFCIILTLVIAIFVLSLVLFCYNHNKTLNLNYPL
KRKTTYIHFLESAVTIVLKPVFQQSLTLRELPIEIASKLLMGLVLLLALFLTSSYGSGLATVMTIPTYENAIN
TVEDFANSGLDWGATQDAWIMSIQNAEEQRYVKIVSKFHPISEEELFQFSKSGKFGFSIERLPFEDYAIGD
YIKEDVIDNFHLMKEDLYWEQCVIMLRKNSVLLPALDLFILKIFEAGLISHWQNEAVDLYMNPKVQRAV
KFYRQGQEHTVVKLQWSHVKGPFALLLIGLCISFIIFILELTLKKKRNQF
>TcasIR41a.2
TLGCLTMTNLNVLLQILLKTYFLNTRCIFLFTDSTIDLQVETPIVYFKVSNTLNPSLIFQHHGCQNILIHHE
NASDIFVQFENLIRLNNERFNERKYIVTGHNSLKILLTKQLEYVSDLLLVVPKQTGHYELITHVYRHQNR
SKINEPVLLDVWYSQNHSFRQENDLFPNKLTNQNQRVLKIGTLSYEPYSVIGKLTVNXSPYYLNLGKDD
YSFDGTETSLVYEFVHKYNLTPSFTIMGDDLWGDVYANWTGIGLFGSVLNDEIDIGYAAVYTWEEYYKF
MDYTKTLIRSGVTCLVPAPQLAAGWVTPLRSFSLGMWIALVIVLLSNTIVLNLLFYRNQKYHXNQLFQIL
LFNAFSKRFFIDSLTTAIKLYVQQPLTLTLKRGLLKYFIVTNMIMVLFISSSYSSGLSSVMTVPRYGKSIQTV
KDLASSHLNWTGTTDAWIFSLRQVEEANYENIKNRFVVKTQNDLVTASKQYNFGFSVERLPYGHYAVG
PYIQRDVICNYRIMQEDLYWGQCTFLLRKNSVLLPLLDKLILRVFEAGLEAYWENQVKCFGRKNMNLR
DFLGCLPIHGHVCPKRHYVLYTTYXEHDTIKLTWEHVEGAFAVLVLGYAASIFTFVIELILDKVRS
>TcasIR68a
MIKNLLPYKCVVLISDDIYGGTFTKSWYRRFGPFITFVVIRVDEYEDLLSPFEETQACLDTAKNEGCQMY
LILLSNALQVSRLLRFGDKYRVINTRAKFVLLYDNRLFDKPLFYLWKRIINVIFIRRYSGQKSDTKKNMP
WYEITTVPFPTQITSILIPRRLDIWTKSKFRKGIDLFRDKTSDLRNQTLKVAAFSHIPGTTKSLQEKTARTVI
GNFSGTEVEILQTVSAAMNFHCELYEPVNVDVDLWGGKQSSGKYTGLVGEMVSTNADIALGDLYYTPY
ILDLMDLSIPYNTECLTFLTPESLTDNSWKTLILPFKYFRPAMWAAVLVCLLICGAVFHALARFHETISQNK
SQVLEIHTKRKKIIILSICPEIEKLDSNLKYTKMREQYKPPRFEGQSIGLYQFSEPFNSVLYTYSMLLLVSLP
KLPTGWSLRMLTGWYWLYCLLLVVAYRASMTAILARPTPRVTIDTLQELVNSRLKCGGWGEINRQFFKS
SLDPITKLIGENFELVNDSNEAVDRVAQGVFAFYENSYYLKEALVKRQLRFQIARTTQNQSEREMRDIAR
EDRNLHIMTDCVIKMPISIGLQKNSPIKPRVDKYIRRVLEAGLIKKWLQDVMASILNAEVQSTQEEMKAI
MNMKKFFGAIVALFIGYFISVVVLIVENVYFHFFVKRNPHYNKYTRSIHHVKKAE
